# Metalation of Tellurophene: Reactivity of 21,23-Ditelluraporphodimethene toward Palladium(II), Platinum(II), and Rhodium(I)

**DOI:** 10.1021/acs.inorgchem.2c03777

**Published:** 2023-01-26

**Authors:** Grzegorz Vetter, Agata Białońska, Ewa Pacholska-Dudziak

**Affiliations:** Department of Chemistry, University of Wroclaw, ul. Joliot-Curie 14, 50-383 Wroclaw, Poland

## Abstract

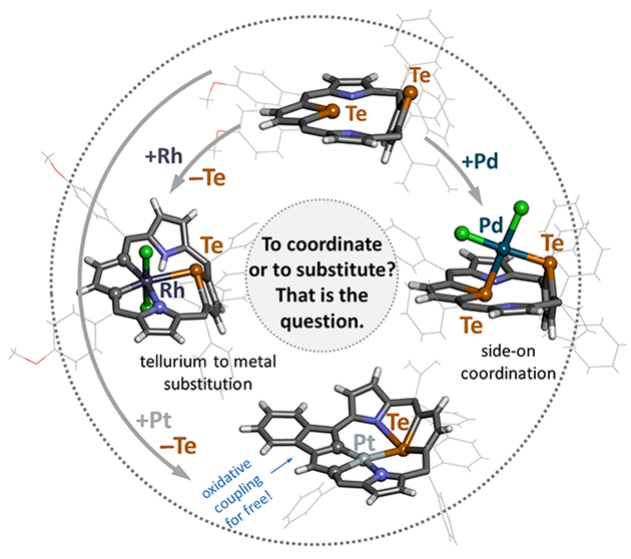

Ditelluraporphodimethene, a nonaromatic porphyrinoid containing two tellurophene rings, reacted with palladium(II), platinum(II), and rhodium(I) following two different paths. Palladium(II) formed bonds to two tellurium donors of the macrocycle, yielding a side-on coordination compound, with a square planar (Te_2_Cl_2_) metal ion environment. An alternative reaction path has been observed for ditelluraporphodimethene with platinum(II) or rhodium(I) in high boiling solvents. These conditions led to the profound transformation, that is, one tellurium atom to a metal atom exchange, resulting in the formation of organometallic species containing metallacyclopentadiene rings, that is, 21-platina-23-telluraporphodimethene and 21-rhoda-23-telluraporphodimethene. The substitution reaction proceeded selectively at the tellurophene ring within the conjugated part of the molecule, that is, the tellurophene ring bound to two sp^2^*meso*-carbon atoms. In the case of platinum, the exchange was accompanied by one *meso*-aryl ring fusion with the formed platinacyclopentadiene ring, and the platinum(II) macrocycle underwent reversible oxidation with chlorine. The products are stable and represent first nonaromatic examples of metalloporphyrinoids, with a metallacyclopentadiene ring incorporated into a porphodimethene skeleton.

## Abstract 2

Hexaaryl-21,23-ditelluraporphodimethene undergoes metalation along two different reaction paths, leading to side-on coordination compounds or tellurium-to-metal substitution products. The substitution products reveal organometallic character, embedding metallacyclopentadiene units within the conjugated moiety of the macrocycle. The flexible tellurophene ring linked to two sp^3^-carbon atoms adjusts the flip angle to the central metal ion preferences.

## Introduction

The tellurophene ring, embedded in an aromatic porphyrin environment, proved to constitute a relatively reactive unit, allowing for various postsynthetic modifications of telluraporphyrins. On the other hand, the reactivity of the tellurophene ring has been altered by its incorporation in the porphyrin frame. The diverse family of telluraporphyrins^1^ includes (1) aromatic species, like 21-telluraporphyrins^2,3^ and its 21-oxo- and 21,21-dihaloderivatives, 21-hetero-23-telluraporphyrins, for example, 21-oxa-23-telluraporphyrin,^4^ 21-carba-23-telluraporphyrin,^5^ dicationic ditelluradiazuliporphyrin;^6^ (2) porphyrins with restrained aromaticity, represented by 21,23-ditelluraporphyrin^7^ and tellura-*para*-benziporphyrin;^8^ and finally (3) nonaromatic tellura-*meta*-benziporphyrin,^9^ tellura-1,5-naphthiporphyrin,^10^ 28-tellura-2,7-naphthiporphyrin,^11^ ditelluraporphodimethenes,^12^ and ditelluraporphyrinogens.^13^ Several aromatic telluraporphyrinoids exhibit tellurophene-centered reactivity, attributed to a relatively weak Te–C bond (ca. 200 kJ·mol^–1^), as compared to other heteroatom–carbon bonds, C–Se (234 kJ·mol^–1^), C–S (272 kJ·mol^–1^), and C–N (305 kJ·mol^–1^). Thus, under oxidative conditions, the tellurophene ring embedded in 21-telluraporphyrin was capable of transforming into the furan ring, to form a 21-oxaporphyrin.^2,3^ A similar transformation, promoted by the presence of a rhodium salt, has been documented in low yield for 21,23-ditelluraporphyrin.^4^ The transformation of a series of aromatic telluraporphyrins, including expanded ditelluraporphyrins, has led to annulene-porphyrin hybrids, in an acid-promoted extrusion of tellurium atom(s), resulting in the formation of the butadiene unit from the tellurophene ring, while the macrocyclic integrity has been conserved.^14−16^

The reactivity of telluraporphyrins toward metal ions does not reproduce the standard scheme known for N_4_-porphyrins, and at the same time, telluraporphyrinoids rarely constitute doubly charged ligands like a regular porphyrin. The canonical in-core metal ion coordination is not the most common in the telluraporphyrin family, limited to date to the palladium(II) complex of benzocarbatelluraporphyrin.^5^ An antiaromatic ditelluraporphyrin analogue, the carbazole-based 21,23-ditelluraisophlorin, allows for palladium(II) incorporation in the very center of the TeNTeN cavity, due to strong tellurophene ring tilt in opposite directions.^17^ Importantly, both the above ligands are doubly negatively charged; thus, the palladium(II) center does not require additional anionic ligands. The large size of the tellurium atom, together with the preference of organotellurium ligands for side-on coordination, promotes strong tellurophene unit inclination in these complexes. For the same reason, a typical metal ion binding mode observed for telluraporphyrins has been a side-on complex formation; however, their collection is still limited.^4,8,9,18,19^ Thus, in crystallographically characterized palladium(II) complexes with 21-tellura-23-vacataporphyrin^18^ and tellura-*m*-benziporphyrin,^9^ both acting as neutral ligands, the metal ion binds to two porphyrin donors (Te and N), and the palladium(II) square planar coordination sphere is practically perpendicular to the porphyrin plane defined by four *meso*-carbons, keeping the metal ion far from the cavity.

An entirely different reactivity toward metal ions, observed uniquely for porphyrins containing the tellurophene unit, follows a substitution path, that is, a heteroatom-to-metal exchange. In such a transformation, the tellurophene ring converted into a metallacyclopentadiene building block embedded in the porphyrin skeleton. Such a metalation has been successfully carried out with palladium(II), platinum(II), and rhodium(I) for 21,23-ditelluraporphyrin, serving as a tellurium-containing precursor, and yielded a new class of organometallic porphyrinoids–metallaporphyrins, exemplified to date by several 21-metalla-23-telluraporphyrins and one 21,23-dimetallaporphyrin (M = Rh).^4,18,19^ Similarly, 21-oxa-23-telluraporphyrin proved reactive toward rhodium(I) and yielded 21-oxa-23-rhodaporphyrin.^4^ In light of the interesting reactivity of aromatic telluraporphyrins with electron-rich metals, we decided to study the possibility of metalation of non-aromatic telluraporphyrinoids, and therefore, a class of hetero-calixphyrins has attracted our attention. In calixphyrins, porphyrin–calixpyrrole hybrids, the macrocyclic π-conjugation pathway is interrupted by one, two, or three sp^3^-carbon *meso*-links, while the remaining bridges are sp^2^ hybridized.^20^ They exhibit significant skeleton flexibility, leading to interesting anion and cation recognition properties. The use of heteroles other than pyrrole rings gives an additional degree of freedom to these molecules, introducing novel coordination environments with different donors, charges, oxidation stages, and architectures, resulting in interesting structural and electronic features.^21^ A series of tellurophene-containing calixphyrins, namely, porphomethenes, porphodimethenes, and porphotrimethenes, with one or two tellurophene rings, compiled with pyrrole or non-pyrrolic building blocks, like selenophene, benzene, or azulene, have been reported recently, widening the library of tellurophene macrocycles.^6,12,13^ Tellurophene and selenophene-containing calixphyrins and porphyrinogenes showed the ability to bind mercury(II) ions, with affinity dependent on the number of sp^3^ bridges.^12,13^

## Results and Discussion

**Scheme 1:**
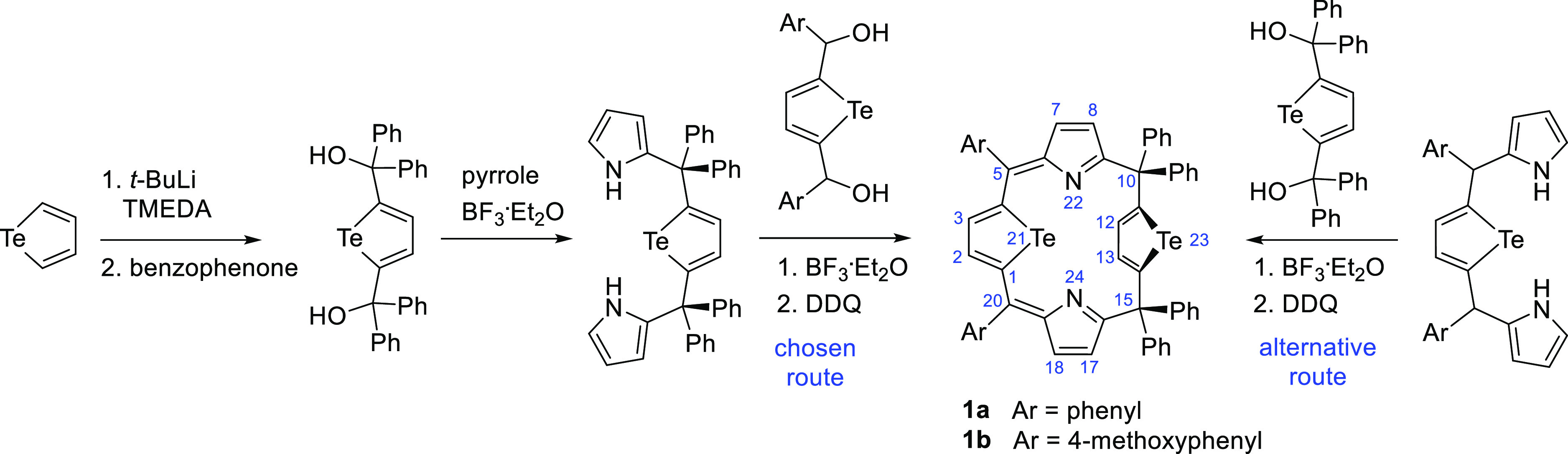
Synthesis of 5,10,10,15,15,20-Hexaaryl-21,23-ditelluraporphodimethenes, **1a** and **1b**; Atom Numbering

Here, we report the reactivity of 21,23-ditelluraporphodimethene, containing two tellurophene units in different environments, toward palladium(II), platinum(II), and rhodium(I). The 5,10,10,15,15,20-hexaaryl-21,23-ditelluraporphodimethene, **1**, has been obtained with a satisfactory yield (35–40%) in a typical synthesis applied previously for differently meso-substituted analogues,^12,13^ that is, according to a [3 + 1] scheme, in an acid-catalyzed condensation of a heterotripyrrane with an appropriate tellurophene-containing diol followed by oxidation (Scheme 1).

The telluratripyrrane precursor incorporated diphenyl-substituted carbon bridges, while the shorter synthon (2,5-bis(phenylhydroxymethyl)tellurophene) was the source of sp^2^*meso*-carbon atoms in the target molecule. An alternative protocol, assembling the macrocycle from the telluratripyrrane with oxidizable monophenyl carbon links and, complementarily, with the tetraphenyl tellurophene diol, gave comparable or even slightly higher yields of the target ditelluraporphodimethene (44% for hexaphenyl derivative); however, the former protocol was preferred due to easier tetraphenyl-substituted telluratripyrrane purification, as compared to the diaryl analogue. Two meso-substitution patterns of ditelluraporphodimethene were employed, differing by sp^2^-carbon link substituents, phenyl rings in **1a** or 4-methoxyphenyl rings in **1b**. Both ligands, **1a** and **1b**, were employed in preliminary studies, and as the differences were not significant for the conclusions, only one series has been fully characterized, decided based on practical reasons like stability or ease of purification. The advantage of the methoxyphenyl series was better crystallization ability; however, the all-phenyl line proved in some cases more stable and gave better yields.

**Scheme 2:**
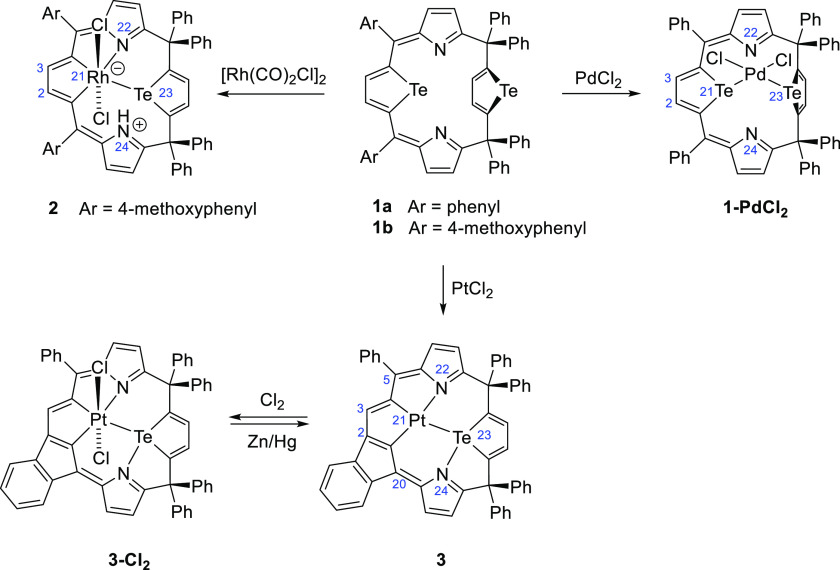
Reactivity of 21,23-Ditelluraporphodimethene **1a** (**1b**) toward Palladium(II), Platinum(II), and Rhodium(I) Yielding a Side-On Complex **1-PdCl**_**2**_ and Tellurium-to-Metal Substitution Products, **2**, **3**, and **3-Cl**_**2**_

Ditelluraporphodimethene, **1**, has been used as a ligand in further studies. After initial metalation tests, the syntheses were optimized to obtain two definitely different types of coordination compounds: (1) the side-on complex, represented by the stable palladium(II) complex **1-PdCl**_**2**_, and (2) organometallic metallaporphodimethenes, represented by rhodium(III) compound (**2**), and a pair of platinum species (**3** and **3-Cl**_**2**_) with a slightly different macrocyclic skeleton (Scheme 2).

**Figure 1:**
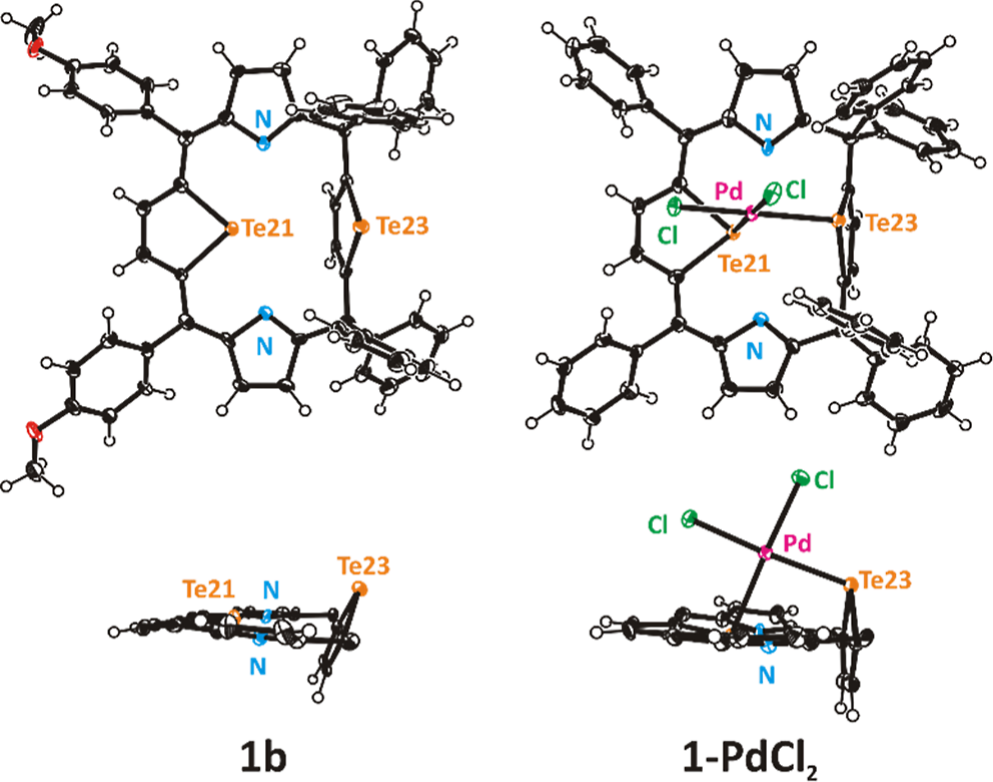
Molecular structures of **1b** and **1-PdCl**_**2**_. Aryl rings are omitted for clarity in side projections. Displacement ellipsoids represent 50% probability.

The first product, the side-on complex **1-PdCl**_**2**_, has been obtained in high yield (85%) from **1a** and Pd(PhCN)_2_Cl_2_ at room temperature. An analogous platinum(II) compound **1-PtCl**_**2**_, with almost identical ^1^H NMR spectral characteristics, has also been detected as a product of the reaction of **1a** and Pt(PhCN)_2_Cl_2_ at relatively low temperatures, in boiling dichloromethane (40 °C). The low stability of **1-PtCl**_**2**_ impeded its full characterization; however, considering spectral similarities, we assume a structure analogous to **1-PdCl**_**2**_. The molecular structure of **1-PdCl**_**2**_ (Figure 1), unambiguously determined by X-ray crystallography, shows that the palladium(II) ion coordinates the porphyrinoid by two tellurium donors, preserving the reflection symmetry of the free ligand (*C*_s_). The palladium(II) square planar coordination sphere is completed by two chloride ligands, with the PdL_4_ plane approximately perpendicular (89°) to the macrocycle plane, defined as a four-*meso*-carbon atoms mean plane. The angles around palladium(II) are close to 90° (85–92°), and the bond lengths Pd–L are typical (Table 1).

**Table 1:** Selected Bond Lengths (in Å) from Crystal Structures

distance	**1b**	**1-PdCl**_**2**_	**2**	**3**	**3-Cl**_**2**_
M–Te		in-plane ring 2.5488(11)	2.8594(9)	2.5892(9)	2.6875(10)
		perpendicular ring 2.5719(7)			
M–N (the closer nitrogen)			2.167(4)	2.060(9)	2.115(8)
Te–N (the closer nitrogen)	Te21–N22 2.760(5)	Te21–N22 2.777(5)	3.119(4)	2.568(9)	2.656(8)
	Te21–N24 2.766(5)	Te21–N24 2.733(5)			
M–Cl		2.3306(18)	2.3457(14)		2.320(3)
		2.3373(15)	2.3465(14)		2.336(3)
Te···N (the distant nitrogen)	Te23···N22 4.202(5)	Te23···N22 3.886(5)	3.397(4)	3.372(9)	3.485(7)
	Te23···N24 4.176(5)	Te23···N22 3.858(5)			
sp^2^-linked metallacycle/tellurophene	tellurophene	tellurophene	rhodacycle	platinacycle	platinacycle
M21/Te21–C1	2.080(6)	2.126(6)	2.050(5),	2.000(11)	2.030(10)
M21/Te21–C4	2.097(6)	2.129(6)	1.945(5)	2.020(11)	2.071(9)
C1–C2 (C_α_C_β_)	1.392(8)	1.364(8)	1.396(7)	1.399(16)	1.361(12)
C2–C3 (C_β_C_β_)	1.406(8)	1.398(9)	1.409(7)	1.414(16)	1.411(13)
C3–C4 (C_α_C_β_)	1.379(8)	1.364(8)	1.377(7)	1.361(15)	1.374(13)
sp^3^-linked tellurophene					
Te23–C11	2.068(6)	2.104(6)	2.101(5)	2.113(11)	2.131(9)
Te23–C14	2.061(6)	2.122(6)	2.098(5)	2.130(11)	2.103(10)
C11–C12 (C_α_C_β_)	1.367(8)	1.334(9)	1.334(7)	1.323(14)	1.330(14)
C12–C13 (C_β_C_β_)	1.386(9)	1.428(9)	1.423(7)	1.452(14)	1.447(14)
C13–C14 (C_α_C_β_)	1.368(8)	1.345(8)	1.353(7)	1.311(14)	1.326(13)

The side-on coordination mode of two tellurophene units accords with the typical behavior of divalent organotellurium ligands, reflected in angles between the tellurophene ring mean plane and the Te–Pd bond, equal to 107° for Te21 and 109° for Te23. In unstrained coordination compounds where divalent tellurium acts as a donor, such an angle typically falls within the range 105–115°.^22^ Palladium(II) binding by **1** to form **1-PdCl**_**2**_ changed the macrocyclic ligand geometry somewhat, rendering two tellurophene rings perpendicular to each other, adjusting Te23 donor orientation to fit the central ion preferences. The tellurophene unit hold by two sp^3^ carbon atoms was able to rotate around two single bonds and change the tilt angle. In the free ligand **1b** (Figure 1), this tellurophene points tellurium-23 outward the macrocyclic core to avoid the steric hindrance between two large tellurium atoms and forming an angle of 111° with the C_4-*meso*_ reference plane. In **1-PdCl**_**2**_**,** the tellurophene is oriented almost perpendicularly to the four-*meso* carbon plane, with slight (2°) inclination inward the macrocyclic core. In both the free ligand and the palladium coordination compound, the telluratripyrrin (Te21) moiety is approximately planar, stabilized by the conjugated π bonds system. Thus, the tellurophene ring (Te23) flanked by two sp^3^ carbon atoms served as a flexible donor, adjusting the tilt angle to the central metal preferences.

Apart from this difference, molecular structures of **1b** and **1-PdCl**_**2**_ exhibit only minor differences. A similar macrocycle geometry and analogous coordination mode as detected in **1-PdCl**_**2**_ have been also observed in our previous studies for a fully π-conjugated ditelluraporphyrinoid, namely, tetraaryl-21,23-ditelluraporphyrin, in a platinum(II) coordination compound;^19^ however, the molecular structure has been elucidated from spectroscopic data and density functional theory (DFT) calculations, since a solid-state crystal structure was lacking. A side-on complex of lower symmetry has been formed by palladium(II) with 21,23-ditelluraporphyrin, where palladium(II) coordinated the macrocycle through one tellurium and one nitrogen.^18^ The observed carbon–carbon bond lengths (Table 1) at the planar moieties of **1b** and in **1-PdCl**_**2**_ are in accordance with the π-conjugation, while the bond alternation is in good accordance with the canonical structures of the macrocycle, as illustrated in Scheme 2.

**Figure 2:**
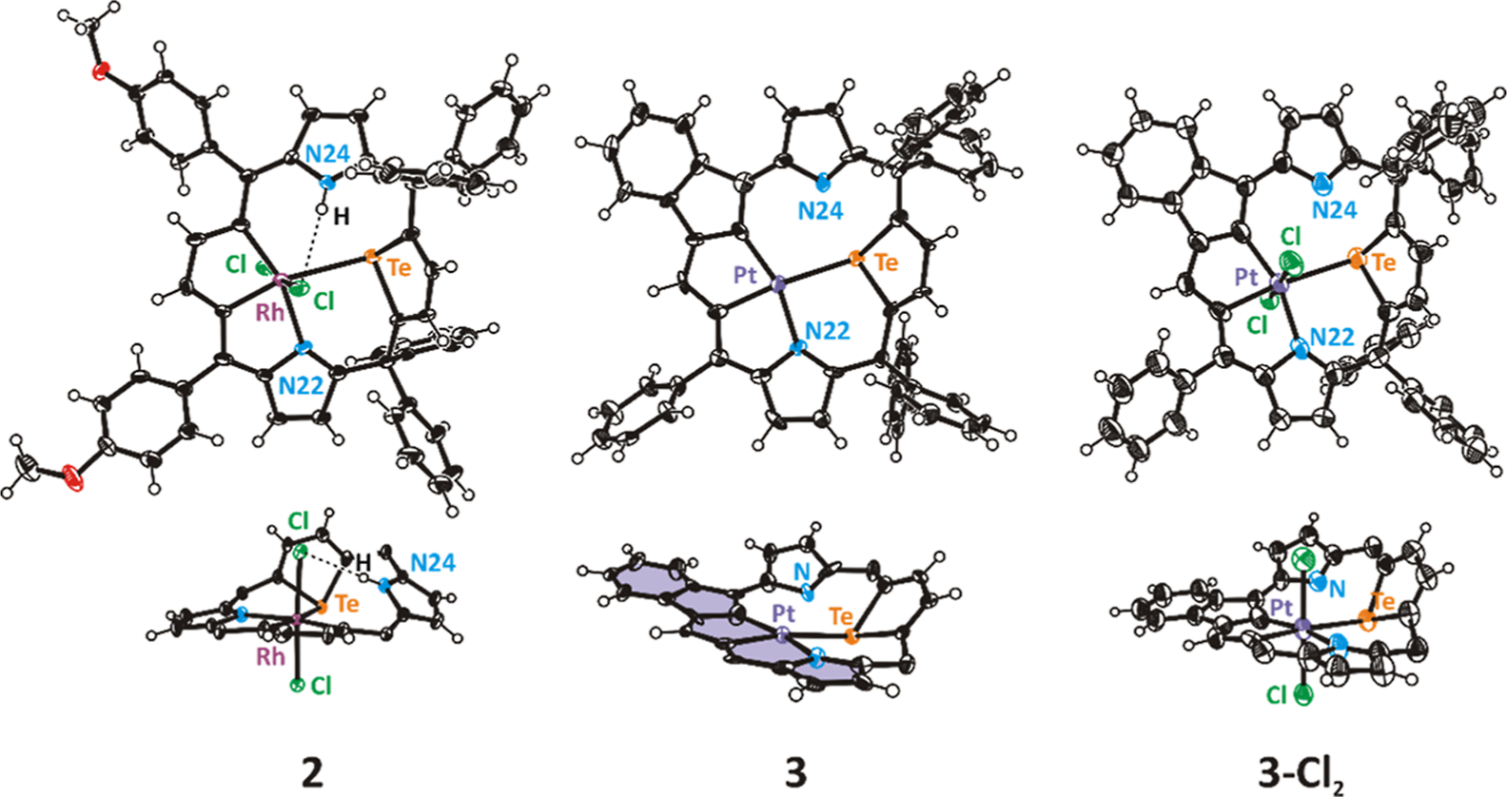
Molecular structures of **2**, **3,** and **3-Cl**_**2**_. Aryl rings are omitted for clarity in side projections. Displacement ellipsoids represent 50% probability.

Entirely different reactivity of ditelluraporphodimethene has been observed toward rhodium(I). The reaction of **1b** with rhodium(I), [Rh(CO)_2_Cl]_2_, performed in boiling toluene gave 21-rhoda-23-telluraporphodimethene, **2**, that is, the organometallic product resulting from the tellurium-to-metal exchange, incorporating the rhodacyclopentadiene unit within the macrocyclic platform. The tellurium-to-rhodium substitution occurred selectively at Te21 of the tellurophene ring between two sp^2^ carbon atoms, that is, in the π-conjugated planar moiety of the molecule. Any products resulting from the other tellurophene ring (Te23) transformation has never been observed. The metalation was accompanied by the oxidation of rhodium(I) to rhodium(III) and by the protonation of one pyrrole nitrogen atom (N24), finally giving a structure with charge separation (Scheme 2). The molecular structure of **2** (Figure 2) displays a strongly folded macrocyclic skeleton with the central rhodium(III) ion in a distorted octahedral environment, surrounded asymmetrically by CCTeN donors of the macrocycle, completed with two axial chlorides. Four *meso*-carbon atoms no longer remain in plane, and molecule **2** no more features a mirror plane, as does the substrate **1**. The rhodacyclopentadiene unit and the pyrrole ring coordinated by N22 to the metal ion are coplanar, while the protonated pyrrole ring (N24) strongly tilts out of the rhodacycle plane. The N24–H vector points toward one axial chloride, stabilizing the structure by an intramolecular hydrogen bond (N···Cl 3.317(4) Å in **2** versus 3.181(6), the mean literature value^23^). The tellurophene ring attached to two sp^3^ carbon atoms is relatively free to rotate and adjusts the tilt angle to the optimal coordination geometry. The tellurophene ring binds to the rhodium ion by the tellurium donor in a typical side-on fashion, forming an angle of 111° with the equatorial RhL_4_ plane (RhC1C4N22Te), while the rhodium–tellurium distance of 2.8594(9) Å is slightly longer than a typical Te–Rh bond, 2.52–2.72 Å.^22^ Thus, the asymmetric molecular shape of **2** is a result of several factors, as follows: the coordination requirements of the rhodium(III) central ion and the tellurium donor, the large size of these atoms (Te and Rh), NH interactions with an axial ligand, and finally, the macrocyclic constraints. In fact, several structural features of **2** resemble those detected in its aromatic analogues, tetraaryl-21-metalla-23-telluraporphyrins,^4,18,19^ in particular the 21-rhoda-23-telluraporphyrin with an *N*-protonated pyrrole ring;^4^ however, the distortion mode was significantly smaller in the latter, where the aromatic stabilization did not allow for such a severe folding of the macrocycle.

**Figure 3:**
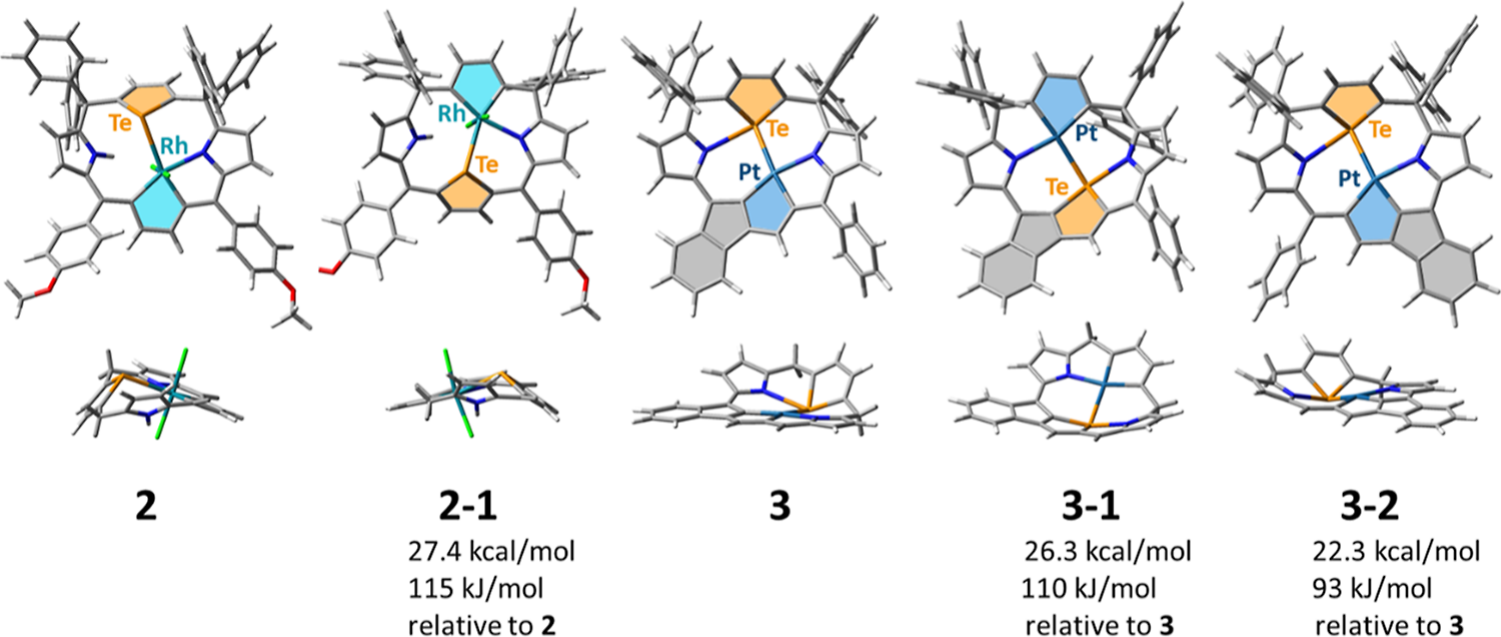
DFT-optimized [B3PW91/6-31G** (for C, N, O, H) and SDD (for Pt, Rh, Te)] structures of **2** and **3**, their isomers with tellurium-metal atoms swapped (**2-1** and **3-1**, respectively), and with fusion of an alternative phenyl ring (**3-2**). The energies include ZPE corrections.

Attempts to perform an analogous tellurium-to-metal exchange in ditelluraporphodimethene with palladium(II) were not successful, neither directly from **1a** (**1b**) in highly boiling solvents nor starting from **1-PdCl**_**2**_. The complex **1-PdCl**_**2**_ was exposed to triethylamine or silver(I) acetate, reagents which promoted transformations of side-on complexes of 21,23-ditelluraporphyrin with palladium(II) and platinum(II), respectively, into 21-metalla-23-telluraporphyrins.^18,19^ In the case of the reaction of **1-PdCl**_**2**_ with triethylamine, demetalation occurred, whereas with silver(I) acetate, no reaction took place; thus, the aromatic 21-pallada-23-telluraporphyrin formation^18^ has no analogy in the porphodimethene chemistry. The tellurium-to-metal substitution has been, however, observed for platinum(II) in the reaction of PtCl_2_ with ditelluraporphodimethene **1a** performed in boiling benzonitrile, which yielded organometallic 21-platina-23-telluraporphodimethene, **3**. The product incorporates the platinacyclopentadiene ring with platinum(II) in place of the substrate’s tellurophene ring of the π-conjugated moiety, and the macrocyclic skeleton is asymmetric, similar to the rhodium(III) congener and 21-metalla-23-telluraporphyrins.^4,18,19^ Moreover, the tellurium-to-platinum substitution is accompanied by an activation of CH groups at the β-position of the metallacyclic ring and at the ortho position of an adjacent *meso*-aryl ring. As a consequence, the aryl ring underwent a fusion reaction with the platinacyclopentadiene unit, yielding a planar π-conjugated tricyclic unit (platina-cyclopenta[*a*]indene structure). Together with the pyrrole ring coordinated to the platinum(II) ion through the N22 donor, a large part of the molecular skeleton forms a relatively planar conjugated block, including five penta- and hexacyclic rings, as marked in violet in Figure 2. The intramolecular coupling occurred selectively with the C20-aryl ring, being energetically favored over C5-aryl fusion, as shown by the DFT calculations. Both isomers, **3** with C20-aryl fused and hypothetical **3-2** with fused C5-aryl, were subjected to DFT geometry optimization at the B3PW91/6-31G** (for C, N, O, H) and SDD (for Pt, Rh, Te) levels of theory. The hypothetical product **3-2** (Figure 3) suffers from angle strain, reflected in higher energy [22.3 kcal/mol (93 kJ/mol)] as compared to **3** and the nonplanar geometry of the pentacyclic unit of **3-2**. The distinct asymmetry of the molecular skeleton of the metallaporphyrinoid differentiates the angles around the *meso*-carbon atoms, resulting in close proximity of *ortho*-C20-aryl and C2, facilitating the intramolecular reaction. Analogous transformations occurring via oxidative intramolecular coupling under the influence of metal salts or organic oxidants, like Fe(III) or DDQ, were employed to extend the porphyrin chromophore.^24,25^

The platinum(II) containing macrocycle **3** has been quantitatively oxidized to a platinum(IV) species by gaseous chlorine. The product, **3-Cl**_**2**_, with two axial chlorides, has generally structural features similar to that of **3** (Figure 2). The zinc amalgam reduces the oxidized product to **3** with a very good yield. The platinum(IV) species was also obtained as a sole macrocyclic product directly in the 21,23-ditelluraporphodimethene metalation with platinum(II) chloride in boiling benzonitrile, provided the bis(methoxyphenyl)-substituted ligand (**1b**) is used. The very low yield, however, impeded its characterization. Thus, in the case of electron-donating substituents on two aryl rings attached to sp^2^ carbons, the air oxidation of platinum(II) compound occurred, contrary to the all-phenyl analogue, which required a stronger oxidant, like chlorine. Easier oxidation of platinum(II) to platinum(IV) coordination compound of a porphyrinoid with electron-donating substituents on aryl rings as compared to the phenyl-substituted ones follows the trend observed for *meso*-tetraarylporphyrins.^26,27^

The platinum(II) central ion in **3** is surrounded by a CCTeN distorted square planar coordination sphere. The analogous environment constitutes the equatorial plane around the platinum(IV) center in **3-Cl**_**2**_, complemented by two axial chlorides to form an irregular octahedron. Contrary to the rhodium(III) analogue, **2**, the platinum compounds **3** and **3-Cl**_**2**_ contain a non-protonated pyrrole ring, with the N24 pointing toward the tellurium atom. The N24–Te separations of 2.568(9) Å in **3** and 2.656(8) Å in **3-Cl**_**2**_ are similar to N–Te distances (2.45–2.59 Å) reported for telluracarbaporphyrins^5^ and indicate binding interactions that control the conformation of the macrocycle. The generally similar skeletons of the two platinum complexes differ in the degree of nonplanarity, which is correlated with the central ion–donor bond lengths. Thus, the shortest metal–donor bonds, exemplified by a Pt^II^–Te distance of 2.5892(9) Å, detected for platinum(II) species, **3**, correlated with a relatively planar structure, that is, the weakest tilt of the tellurophene ring from the PtCCNTe mean plane (130°). Following typical trends, the platinum(IV) macrocycle, **3-Cl**_**2**_, shows longer bonds, with Pt^IV^–Te bond equal to 2.6875(10) Å, and a more pronounced tellurophene tilt (analogous angle 117°). The rhodium(III) species, **2**, follows this correlation, with an even longer Rh–Te bond [2.8594(9) Å] and a very upright tellurophene unit (111°).

In the tellurium-to-platinum substitution reaction, solely the product of the transformation of the π-conjugated tellurophene between two sp^2^ carbon atoms has been detected, similar to that in the case of the reaction with rhodium(I). The DFT studies complement the experimental findings and rationalize the selectivity observed in the tellurium-to-metal exchange. Along with **2** and **3**, their isomers, **2-1** and **3-1**, were taken into consideration, respectively, with a metal atom situated between two tetrahedral carbon atoms, while the π-conjugated moiety contained tellurophene. The two pairs of isomers were optimized, and the calculations revealed large energy differences between **2** and **2-1** (27.4 kcal/mol, 115 kJ/mol) and between **3** and **3-1** (26.3 kcal/mol, 110 kJ/mol), which account for the syntheses selectivity. The optimized geometries of hypothetical structures **2-1** and **3-1** show a distinct tilt of the tellurophene ring from the plane of the conjugated moiety, which must be less favorable than the rotation of a tellurophene ring around single bonds with sp^3^ carbon atoms, observed in **2** and **3**. Moreover, in **3-1**, the conjugated pentacyclic unit shows distinct nonplanarity, in contrast to **3**.

Taking into account several analogies between 21,23-ditelluraporphodimethene and 21,23-ditelluraporphyrin metalation products observed for palladium, platinum, and rhodium, as depicted above and reported previously,^4,18,19^ we postulate that the reaction path leading from **1** to 21-metalla-23-porphodimethenes is similar to that recognized in our former studies for 21,23-ditelluraporphyrin. Thus, the tellurium-to-metal exchange commences with a side-on complex formation, then follows an insertion of a metal ion into the C–Te bond, with the formation of a reactive species including a six-membered TeMC_4_ ring, and finally an extrusion of tellurium occurs to obtain a metallaporphyrinoid. Although for porphodimethene we did not observe several species of a reaction sequence for one metal, we isolated a stable complex **1-PdCl**_**2**_ and observed less stable **1-PtCl**_**2**_, representing the postulated first metalation step. The binding of palladium(II) ion to **1** was accompanied by a slight elongation of Te–C bond lengths, as compared to the free ligand [Te–C1 2.080(6) Å in **1b** vs 2.126(6) in **1-PdCl**_**2**_; Te–C11 2.068(6) in **1b** vs 2.104(6) in **1-PdCl**_**2**_; Table 1], which may be interpreted as bond activation. Moreover, in both **1b** and **1-PdCl**_**2**_, the Te–C bonds are longer in the conjugated tellurophene, where the metal insertion occurs (Te–C1 2.080(6) Å vs Te–C11 2.068(6) in **1b**; Te–C1 2.126(6) vs Te–C11 2.104(6) in **1-PdCl**_**2**_), compared to the isolated ring.

## Spectroscopic Studies

**Figure 4:**
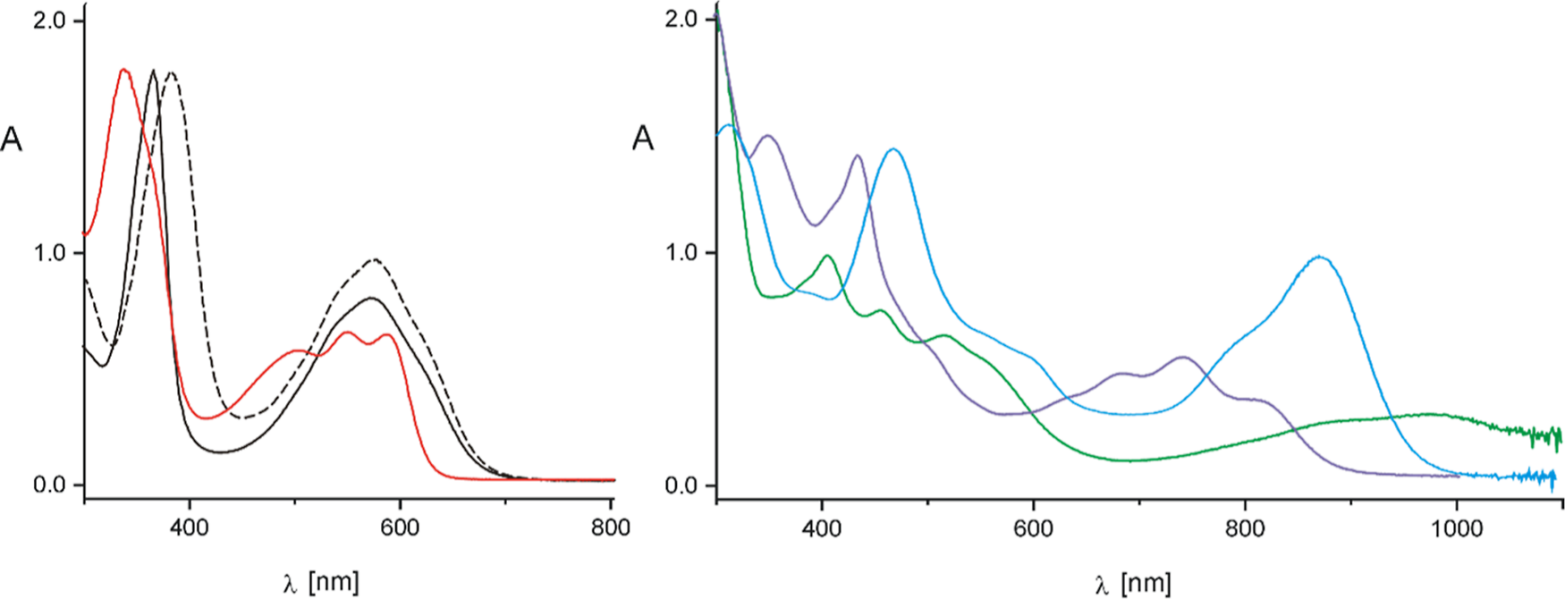
UV–vis spectra (CH_2_Cl_2_) of **1a** (black solid line), **1b** (black dashed line), **1-PdCl**_**2**_ (red), **2** (blue), **3** (green), and **3-Cl**_**2**_ (purple).

The electronic spectra of the reported macrocycles are shown in Figure 4. Free ligand spectra are typical for hetero-5,10-porphodimethenes^12,28,29^ and show two absorption bands of comparable intensity, one narrower below 400 nm and a broader shouldered band in the range 500–600 nm. For **1a,** the more intense short-wave band has a maximum at 368 nm, and the change of the *meso*-aryl substituents at sp^2^-carbon links from phenyl to *p*-methoxyphenyl causes a red shift to 383 nm for **1b**. The broad longer wave bands centered at 574 nm for **1a** and 576 nm for **1b** have shoulders at both sides and do not shift much on the substituents change.

The change in the electronic structure accompanying palladium(II) ion binding and macrocyclic skeleton conformation alteration is reflected in a blue shift of all the bands (short wave band: 342 nm **1-PdCl**_**2**_ vs 368 nm **1a**). The long wave band with two shoulders in the ligand changed to three resolved bands in the palladium(II) coordination compound. The formation of 21-rhoda-23-telluraporphodimethene **2** from **1b**, associated with profound macrocyclic skeleton changes, shifts significantly the absorption to longer waves, showing an intense band as far as at 879 nm, while two other bands are visible at 471 and 315 nm. The platinum(II) and platinum(IV) species spectra do not resemble patterns found for the rhodium(III) compound **2** nor show any mutual similarity. The prominent feature of the spectrum of **3** is a broad far red-shifted band at 978 nm.

**Figure 5:**
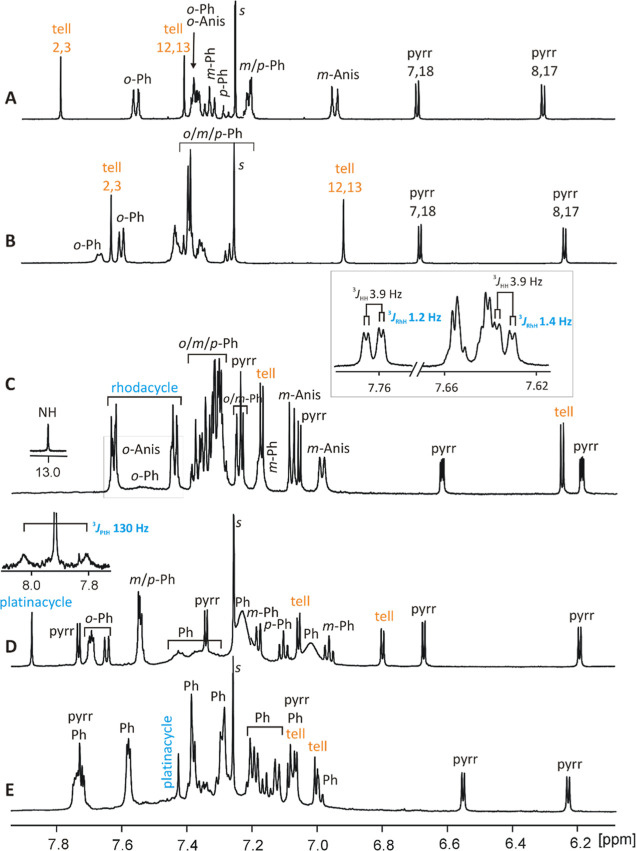
^1^H NMR (600 MHz, 300 K) spectra of (A) **1b** (CDCl_3_), (B) **1-PdCl**_**2**_ (CDCl_3_), (C) **2** (CD_2_Cl_2_; inset: C_6_D_6_, 350 K), (D) **3** (CDCl_3_; inset: C_6_D_6_, 350 K), and (E) **3-Cl**_**2**_ (CDCl_3_).

^1^H NMR characteristics of the described porphyrinoids **1**, **1-PdCl**_**2**_, **2**, **3** and **3-Cl**_**2**_ are in accordance with their molecular structures detected in the solid state and reflect molecules symmetry (Figure 5). For all the species, the chemical shift span for β-pyrrolic protons, 6.0–6.7 ppm, is characteristic of porphyrinoids lacking macrocyclic aromaticity, as expected for porphodimethenes having sp^3^*meso*-bridges. The free ligand **1** (spectrum A) and the side-on complex **1-PdCl**_**2**_ (B) show very clear spectra with two β-tellurophene singlets diagnostic of the twofold symmetry of these molecules (*C*_s_). The more downfield shifted singlet of each spectrum (7.8–7.6 ppm) is assigned to the π-conjugated moiety, while the flexible tellurophene unit is more upfield and undergoes a distinct shift change on palladium coordination (from 7.4 ppm in **1** to 6.9 ppm in **1-PdCl**_**2**_). The formation of 21-metalla-23-telluraporphodimethenes lowers the molecular symmetry to *C*_1_, thus doubling the number of signals. For **2** and **3** (spectra C and D), the β-protons of metallacyclopentadiene rings, involved in the π-conjugation, have relatively large chemical shifts (7.9–7.4 ppm in CDCl_3_) and due to the presence of spin active nuclei, ^103^Rh (*I* = 1/2, 100%) and ^195^Pt (*I* = 1/2, 33.8%), show patterns which are diagnostic for these structures. The rhodacyclopentadiene protons in **2** give characteristic doublets of doublets (^3^*J*_HH_ = 3.9 Hz, ^3^*J*_RhH_ = 1.2–1.4 Hz), best resolved in the spectrum measured in deuterated benzene at 350 K. These relatively small coupling constants are similar to those observed for aromatic rhodaporphyrins (^3^*J*_HH_ 4.6–5.2 Hz; ^3^*J*_RhH_ 0.7–2.3 Hz).^4^

The protonated pyrrole ring in **2** gives rise to a relatively narrow and a downfield-shifted NH signal at 13.1 ppm, deshielded due to interactions with an axial chloride. In the spectrum of **3** (D), the most downfield-shifted singlet has been assigned to the platinacyclic unit on the basis of platinum satellite presence, severely broadened due to the chemical shift anisotropy relaxation, which, however, becomes narrower at elevated temperatures. The ^3^*J*_PtH_ = 130 Hz coupling constant (C_6_D_6_, 350 K) is slightly larger than that in aromatic platinaporphyrinoids, containing a platinum(II) center (98–116 Hz).^19^ The overlapping of signals in the spectrum of platinum(IV) compound, **3-Cl**_**2**_ (E), did not allow for the ^195^Pt satellite observation. In the case of rhodaporphodimethene, **2**, the ^13^C NMR spectrum was of interest likewise, as very large chemical shifts (215 and 182 ppm) were observed for α-rhodacyclopentadiene carbons. Similar values, however, were already observed for other rhodaporphyrins and are also recognized by characteristic ^13^C–H one-bond coupling constants, ^1^*J*_RhC_ of 27 and 31 Hz, characteristic of ^103^Rh–^13^C constants for sp^2^ carbons, similar to those observed for other porphyrinoids (23–26 Hz), and are much smaller than those in the case of C≡O axial ligands (*J* ∼ 70 Hz), which give similar chemical shifts.^4,30^

## Conclusions

21,23-ditelluraporphodimethene may serve as a neutral bidendate ligand, binding a metal ion to two tellurium donors in a side-on fashion. Alternatively, the metalation reaction may follow the substitution path, leading to the transformation of the tellurophene ring into a metallacyclopentadiene unit. Thus, a series of organometallic 21-metalla-23-telluraporphodimethenes, formally belonging to a class of heteroporphyrinoids, incorporating platinacyclopentadiene or rhodacyclopentadiene unit, have been obtained in a straightforward postsynthetic modification of 21,23-ditelluraporphodimethenes. The tellurium-to-metal exchange proved effective for a porphyrinoid devoid of macrocyclic aromaticity; however, the π-delocalization played a role in the reaction regioselectivity. The products are stable organometallic species, which can be placed at a crossroad of carbaporphyrinoids and porphodimethenes.

## Methods

### X-ray Diffraction Data

Single-crystal X-ray diffraction data were collected at 100 K, on KUMA Xcalibur (Sapphire2 CCD detector) (**1b**, **1-PdCl**_**2**_, **2**, **3**) or Rigaku XtaLAB Synergy R, DW system (HyPix-Arc 150) (**3-Cl**_**2**_) κ-geometry diffractometers using Mo Kα or Cu Kα radiation. Data reduction and analysis were carried out with the CrysAlis Pro programs (CrysAlis PRO. CrysAlisPro: Rigaku Oxford Diffraction *1.171.33.52, 1.171.33.66, 1.171.36.28, 1.171.41.80a*). The structures were solved by direct methods and refined with the full-matrix least-squares technique using the *SHELXS*(31) and *Shelxl-2018/3*(32) programs. Hydrogen atoms were placed at calculated positions or were found on the Δρ map. Before the last cycle of refinement, all H atoms were fixed and were allowed to ride on their parent atoms. Anisotropic displacement parameters were refined for all non-hydrogen atoms. However, SIMU or ISOR restraints were applied in **1b**, **3**, and **3-Cl**_**2**_. The geometry of the disordered hexane molecule in **3-Cl**_**2**_ was restrained by applying the SADI command. The occupancy factor for the disordered components was refined.

### DFT Calculations

DFT calculations were performed using the Gaussian 16 program.^33^ DFT geometry optimizations were carried out in the unconstrained *C*_1_ symmetry in vacuo, using the X-ray structures or molecular mechanics models as starting geometries. The existence of a local energy minimum was verified by a normal mode frequency calculation. All DFT calculations were performed using the hybrid functional B3PW91 and a combined basis set consisting of the SDD pseudopotential for Rh, Pt, and Te atoms and 6-31G(d,p) for remaining atoms. All relative energies include the zero-point correction. Electronic spectra were obtained from TD-DFT calculations (see the Supporting Information) with the number of states equal to 60. The electronic transitions and UV–vis simulated spectra were analyzed by means of the GaussSum program.^34^

#### Synthesis of **1a**

BF_3_·Et_2_O (20 μL, 0.18 mmol) was added to 2,5-bis(phenylhydroxymethyl)-tellurophene (0.136 g, 0.35 mmol) and 2,5-bis(2-pyrrolo(diphenyl)methyl)tellurophene (0.224 g, 0.35 mmol) in 120 mL of degassed CH_2_Cl_2_, and the reaction mixture was stirred for 1 h in the dark at ambient temperature. Note: tellurium-containing precursors are malodorous and must be handled in a well-ventilated fume hood. DDQ (0.158 g, 0.70 mmol) was added, and the resulting mixture was stirred for another hour at ambient temperature. The reaction mixture was filtered through deactivated Al_2_O_3_ III. The solvent was evaporated, the mixture was purified by column chromatography on SiO_2_, and **1a** was eluted as a second navy-blue band with a 1:2 mixture of CH_2_Cl_2_ and hexanes (40% yield). ^1^H NMR (CDCl_3_, 300 K, 500 MHz): δ 7.74 (s, 2H, tell), 7.58 (m, 4H, *o*-Ph), 7.44 (s, 2H, tell), 7.43 (m, 14H, *o*/*m*/*p*-Ph), 7.35 (m, 4H, *m*-Ph), 7.29 (m, 2H, *p*-Ph), 7.24 (m, 6H, *m*/*p*-Ph), 6.68 (d, ^3^*J*_HH_ = 4.5 Hz, 2H, pyrr), 6.33 (d, ^3^*J*_HH_ = 4.5 Hz, 2H, pyrr); ^1^H NMR (C_6_D_6_, 300 K, 600 MHz): δ 7.84 (s, 2H, tell), 7.71 (s, 2H, tell), 7.68 (m, 4H, *o*-Ph), 7.63 (m, 4H, *o*-Ph), 7.25 (m, 4H, *o*-Ph), 7.08 (m, 12H, *m*-Ph), 6.92 (m, 6H, *p*-Ph), 6.65 (d, ^3^*J*_HH_ = 4.5 Hz, 2H, pyrr), 6.31 (d, ^3^*J*_HH_ = 4.5 Hz, 2H, pyrr); ^13^C NMR (C_6_D_6_, 300 K, 150 MHz): δ 178.3 (α-pyrr), 162.2 (α-tell), 159.7 (α-tell), 152.7 (α-pyrr), 151.6 (meso), 147.9 (ipso), 144.1 (β-tell), 143.4 (ipso), 142.2 (β-tell), 136.9 (ipso), 133.3 (β-pyrr), 131.0 (*o*-Ph), 130.3 (*o*-Ph), 130.2 (β-pyrr), 128.5 (*o*-Ph), 128.3 (*p*-Ph), 128.1 (*m*-Ph), 127.9 (*p*-Ph), 126.6 (*m*-Ph), 64.4 (meso); UV–vis (nm, log ε) = 574 (4.3), 368 (4.6); HRMS (ESI) *m*/*z*: 999.1196, calcd for C_56_H_38_N_2_^130^Te_2_, [M + H]^+^: 999.1248.

#### Synthesis of **1b**

BF_3_·Et_2_O (20 μL, 0.13 mmol) was added to 2,5-bis(4-methoxyphenylhydroxymethyl)tellurophene (0.115 g, 0.26 mmol) and 2,5-bis(2-pyrrolo(diphenyl)methyl)tellurophene (0.164 g, 0.26 mmol) in 120 mL of degassed CH_2_Cl_2_, and the reaction mixture was stirred for 1 h in the dark at ambient temperature. DDQ (0.118 g, 0.52 mmol) was added, and the resulting mixture was stirred for another hour at ambient temperature. The reaction mixture was filtered through deactivated Al_2_O_3_ III. The solvent was evaporated, the mixture was purified by column chromatography on SiO_2_, and **1b** was eluted as a third navy-blue band with a 1:2 mixture of CH_2_Cl_2_ and hexanes (35% yield). ^1^H NMR (CDCl_3_, 300 K, 500 MHz): δ 7.80 (s, 2H, tell), 7.56 (m, 4H, *o*-Ph), 7.41 (s, 2H, tell), 7.38 (m, 8H, *o*-Anis, *o*-Ph), 7.34 (m, 4H, *m*-Ph), 7.28 (m, 2H, *p*-Ph), 7.21 (m, 6H, *m*/*p*-Ph), 6.95 (m, 4H, *m*-Anis), 6.70 (d, ^3^*J*_HH_ = 4.5 Hz, 2H, pyrr), 6.31 (d, ^3^*J*_HH_ = 4.5 Hz, 2H, pyrr), 3.86 (s, 6H, OMe); ^13^C NMR (CDCl_3_, 300 K, 125 MHz): δ 177.6 (α-pyrr), 161.7 (α-tell), 160.0 (para), 159.3 (α-tell), 152.5 (α-pyrr), 151.1 (meso), 147.8 (ipso), 143.9 (β-tell), 143.4 (ipso), 141.9 (β-tell), 133.4 (β-pyrr), 132.0 (*o*-Ph), 130.9 (*o*-Ph), 130.0 (β-pyrr), 129.1 (ipso), 128.4 (*o*-Anis, *m*-Ph), 127.8 (*m*-Ph), 127.4 (*p*-Ph), 126.8 (*p*-Ph), 113.2 (*m*-Anis), 64.0 (meso), 55.4 (OMe); UV–vis (nm, log ε) = 576 (4.2), 383 (4.4); HRMS (ESI) *m*/*z*: 1059.1127, calcd for C_58_H_42_N_2_O_2_^130^Te_2_, [M + H]^+^: 1059.1458. Crystal data for compound **1b**: C_58_H_42_N_2_O_2_Te_2_, CHCl_3_, *M* = 1173.50, triclinic, *P*1̅, *a* = 11.519(3) Å, *b* = 14.756(3) Å, *c* = 16.463(3) Å, α = 65.20(4)°, β = 73.04(3)°, γ = 81.44(3)°, *V* = 2428.6(12) Å^3^, *Z* = 2, *D*_c_ = 1.605 Mg m^–3^, *T* = 100(2) K, *R* = 0.0606, *wR* = 0.1279 [6535 reflections with *I* > 2σ(*I*)] for 650 variables, CCDC 2214345.

#### Synthesis of **1-PdCl**_**2**_

4 mg (4.0 × 10^–6^ mol) of **1a** and 7.7 mg (2.0 × 10^–5^ mol) of Pd(PhCN)_2_Cl_2_ were dissolved in 10 mL chloroform. The nitrogen was bubbled through the mixture for 15 min, and the solution was stirred for another day. The solvent was evaporated, the product was purified by column chromatography on SiO_2_, and **1-PdCl**_**2**_ was eluted as a pink band with 0.5% ethyl acetate in CH_2_Cl_2_ (85% yield). ^1^H NMR (CDCl_3_, 300 K, 600 MHz): δ 7.66 (m, 2H, *o*-Ph), 7.63 (s, 2H, tell), 7.60 (m, 4H, *o*-Ph), 7.43–7.27 (m, 24H, *o*/*m*/*p*-Ph), 6.92 (s, 2H, tell), 6.69 (d, ^3^*J*_HH_ = 4.5 Hz, 2H, pyrr), 6.25 (d, ^3^*J*_HH_ = 4.5 Hz, 2H, pyrr); ^13^C NMR (CDCl_3_, 300 K, 150 MHz): δ 177.4 (α-pyrr), 161.2 (α-tell), 155.9 (α-tell), 152.9 (α-pyrr), 148.8 (β-tell), 144.8 (meso), 142.8 (β-tell), 139.8 (ipso), 135.5 (ipso), 133.6 (β-pyrr), 132.2 (β-pyrr), 131.9 (*o*-Ph), 131.0 (Ph), 130.3 (*o*-Ph), 129.4 (Ph), 129.2 (Ph), 128.9 (*m*-Ph), 128.6 (*o*-Ph), 128.5 (Ph), 128.1 (Ph), 128.0 (Ph), 127.8 (Ph), 64.1 (meso); UV–vis (nm, log ε) = 590 (4.0), 553 (4.0), 505 (4.0), 342 (4.5); HRMS (ESI) *m*/*z*: 1140.9929, calcd for C_56_H_38_ClN_2_^106^Pd^130^Te_2_, [M – Cl]^+^: 1140.9980. Crystal data for compound **1-PdCl**_**2**_: C_56_H_38_Cl_2_N_2_PdTe_2_, 3(CHCl_3_), *M* = 1529.49, monoclinic, *P*2_1_/*n*, *a* = 13.186(3) Å, *b* = 26.999(4) Å, *c* = 16.744(7) Å, β = 106.85(3)°, *V* = 5705(3) Å^3^, *Z* = 4, *D*_c_ = 1.781 Mg m^–3^, *T* = 100(2) K, *R* = 0.0649, *wR* = 0.1213 [9292 reflections with *I* > 2σ(*I*)] for 676 variables, CCDC 2214346.

#### Synthesis of **1-PtCl**_**2**_

5 mg (5.0 × 10^–6^ mol) of **1a** and 4.7 mg (1.0 × 10^–5^ mol) of Pt(PhCN)_2_Cl_2_ were dissolved in 10 mL dichloromethane. The nitrogen was bubbled through the mixture for 15 min, and the solution was refluxed for another hour. The solvent was evaporated, the product was purified by column chromatography on SiO_2_, and **1-PtCl**_**2**_ was eluted as a pink band with 1% ethanol in CH_2_Cl_2_ (80% yield). ^1^H NMR (CDCl_3_, 300 K, 500 MHz): δ 7.69 (m, 4H, *o*-Ph), 7.66 (m, 2H, *o*-Ph), 7.58 (s, 2H, tell), 7.44–7.36 (m, 24H, *o*/*m*/*p*-Ph), 6.79 (s, 2H, tell), 6.70 (d, ^3^*J*_HH_ = 4.6 Hz, 2H, pyrr), 6.23 (d, ^3^*J*_HH_ = 4.6 Hz, 2H, pyrr). UV–vis (nm) = 579, 545, 389, 353; HRMS (ESI) *m*/*z*: 1228.0545, calcd for C_56_H_38_ClN_2_^195^Pt^130^Te_2_, [M – Cl]^+^: 1228.0493.

#### Synthesis of **2**

Ligand **1b** (23 mg, 2.2 × 10^–5^ mol) and [Rh(CO)_2_Cl]_2_ (8.5 mg, 2.2 × 10^–5^ mol) were dissolved in 15 mL of toluene. Nitrogen was bubbled through the mixture for 15 min, and the solution was refluxed for another 30 min. The solvent was evaporated and the products were purified by column chromatography on SiO_2_ with CH_2_Cl_2_; **2** was eluted as the second brown band (16% yield). ^1^H NMR (CD_2_Cl_2_, 300 K, 600 MHz): δ 13.09 (br s, 1H, NH), 7.63 (m, 3H, rhodacycle*, *o*-Anis), 7.54 (m, 2H, *o*-Ph), 7.45 (m, 3H, rhodacycle*, *o*-Anis), 7.39–7.29 (m, 13H, *o*/*m*/*p*-Ph), 7.24 (m, 4H, pyrr, *o*/*m*-Ph), 7.18 (m, 3H, tell, *m*-Ph), 7.09 (m, 2H, *m*-Anis), 7.06 (d, ^3^*J*_HH_ = 4.6 Hz, 1H, pyrr), 7.00 (m, 2H, *m*-Anis), 6.63 (dd, ^3^*J*_HH_ = 4.3 Hz, ^4^*J*_HH_ = 2.5 Hz, 1H, pyrr), 6.26 (d, ^3^*J*_HH_ = 4.9 Hz, 1H, tell), 6.20 (dd, ^3^*J*_HH_ = 4.3 Hz, ^4^*J*_HH_ = 2.5 Hz, 1H, pyrr), 3.90 (s, 3H, OMe), 3.89 (s, 3H, OMe); ^13^C NMR (CD_2_Cl_2_, 300 K, 150 MHz): δ 215.3 (d, ^1^*J*_RhC_ = 27 Hz, α-rhodacycle), 182.7 (d, ^1^*J*_RhC_ = 31 Hz, α-rhodacycle), 178.3 (meso), 177.9 (α-tell), 176.7 (β-rhodacycle), 164.7 (para), 161.5 (para), 160.6 (α-tell), 159.3 (α-pyrr), 157.4 (α-pyrr), 155.7 (α-pyrr), 155.3 (meso), 149.1 (β-rhodacycle), 146.0 (ipso), 145.9 (ipso), 143.2 (ipso), 141.6 (ipso), 141.3 (β-pyrr), 138.1 (β-pyrr), 137.8 (α-pyrr), 135.5 (β-tell), 133.6 (ipso), 132.7 (*o*-Anis), 131.3 (*m*-Ph) 130.8 (*o*/*m*-Ph), 130.2 (*o*-Anis), 130.0 (Ph), 129.5 (β-pyrr), 129.0 (β-tell), 128.9 (Ph), 128.8 (Ph), 128.7 (Ph), 128.6 (Ph), 128.3 (Ph), 128.2 (Ph), 128.0 (Ph), 127.9 (Ph), 126.5 (ipso), 119.1 (β-pyrr), 114.6 (*m*-Anis), 114.4 (*m*-Anis), 64.3 (meso), 62.3 (meso), 56.3 (OMe), 56.0 (OMe); UV–vis (nm, log ε) = 879 (3.9), 471 (4.1), 315 (4.1); HRMS (ESI) *m*/*z*: 1125.0716, calcd for C_58_H_43_Cl_2_N_2_O_2_Rh^130^Te, [M + Na]^+^: 1125.0712. *^103^Rh splitting of the rhodacyclopentadiene signals was detected in following conditions: C_6_D_6_, 350 K, 600 MHz: 7.76 (dd, ^3^*J*_RhH_ = 1.2 Hz, ^3^*J*_HH_ = 3.9 Hz), 7.63 (dd, ^3^*J*_RhH_ = 1.4 Hz, ^3^*J*_HH_ = 3.9 Hz). Crystal data for compound **2**: C_58_H_43_Cl_2_N_2_O_2_RhTe, 2(CHCl_3_), *M* = 1340.09, monoclinic, *P*2_1_/*c*, *a* = 11.759(3) Å, *b* = 20.403(3) Å, *c* = 23.040(4) Å, β = 98.85(2)°, *V* = 5461.9(19) Å^3^, *Z* = 4, *D*_c_ = 1.630 Mg m^–3^, *T* = 100(2) K, *R* = 0.0475, *wR* = 0.1158 [6762 reflections with *I* > 2σ(*I*)] for 667 variables, CCDC 2214347.

#### Synthesis of **3**

10.7 mg (4.0 × 10^–5^ mol) of PtCl_2_ was dissolved in 10 mL benzonitrile. The nitrogen was bubbled through the mixture for 15 min, and the solution was heated until the solid was dissolved completely. At that point, 10 mg (1.0 × 10^–5^ mol) of **1a** in a little amount of benzonitrile was added, and the solution was refluxed for about 30 min (violet-to-brown color change). The solvent was evaporated, the product was purified by column chromatography on SiO_2_, and **3** was eluted as a red band with a 1:1 mixture of CH_2_Cl_2_ and hexanes (14% yield). ^1^H NMR (CDCl_3_, 300 K, 600 MHz): δ 7.88 (s, 1H, platinacycle; ^3^*J*_PtH_ = 130 Hz detected in C_6_D_6_, 350 K), 7.74 (d, ^3^*J*_HH_ = 4.6 Hz, 1H, pyrr), 7.70 (m, 2H, *o*-Ph), 7.65 (m, 1H, *o*-Ph), 7.55 (m, 3H, *m*/*p*-Ph), 7.43 (br, 6H, Ph), 7.35 (d, ^3^*J*_HH_ = 4.6 Hz, 1H, pyrr), 7.23 (br, 10H, Ph) 7.18 (m, 1H, *m*-Ph), 7.09 (m, 1H, *p*-Ph), 7.06 (d, ^3^*J*_HH_ = 4.7 Hz, 1H, tell), 7.02 (br, 4H, Ph), 6.97 (m, 1H, *m*-Ph), 6.80 (d, ^3^*J*_HH_ = 4.7 Hz, 1H, tell), 6.67 (d, ^3^*J*_HH_ = 4.6 Hz, 1H, pyrr), 6.19 (d, ^3^*J*_HH_ = 4.6 Hz, 1H, pyrr); ^13^C NMR (CDCl_3_, 300 K, 150 MHz): δ 178.8 (α-platinacycle), 177.9 (β-platinacycle), 170.9 (α-pyrr), 165.4 (α-pyrr), 164.1 (α-pyrr), 162.5 (α-platinacycle), 159.2 (α-tell), 154.0 (meso), 153.3 (meso), 149.2 (α-tell), 145.4 (α-pyrr), 143.7 (*o*-Ph), 140.7 (ipso), 138.4 (β-platinacycle), 136.9 (β-tell), 135.8 (β-pyrr), 135.4 (ipso), 135.0 (β-tell), 133.9 (β-pyrr), 130.2 (*o*-Ph), 130.1 (*p*-Ph), 129.4 (br, Ph), 129.0 (ipso), 128.7 (*m*/*p*-Ph), 128.6 (br, β-pyrr, Ph), 128.1 (br, Ph), 127.5 (Ph), 127.3 (β-pyrr, Ph), 126.6 (*m*-Ph), 123.8 (*o*-Ph), 120.7 (Ph), 120.6 (*m*-Ph), 64.6 (meso), 62.8 (meso); UV–vis (nm, log ε) = 978 (3.6), 515 (3.9), 455 (4.0), 404 (4.2); HRMS (ESI) *m*/*z*: 1062.1753, calcd for C_56_H_36_N_2_^195^Pt^130^Te, [M + H]^+^: 1062.1669. Crystal data for compound **3**: C_56_H_36_N_2_PtTe, 0.5(C_6_H_6_), *M* = 1098.61, trigonal, *R*3̅, *a* = 41.235(3) Å, *c* = 12.904(2) Å, *V* = 19001(4) Å^3^, *Z* = 18, *D*_c_ = 1.728 Mg m^–3^, *T* = 100(2) K, *R* = 0.0745, *wR* = 0.1135 [4200 reflections with *I* > 2σ(*I*)] for 568 variables, CCDC 2226013.

#### Synthesis of **3-Cl**_**2**_

To the solution of **3** (5 mg, 4.7 × 10^–6^ mol) in chloroform (10 mL), chlorine gas (4.7 × 10^–6^ mol) was added, and the solution was stirred at room temperature for 5 min. Caution: chlorine gas is toxic and corrosive and must be handled according to appropriate safety rules. After evaporation of the solvent, the product was purified by column chromatography on SiO_2_ and **3-Cl**_**2**_ was eluted with CH_2_Cl_2_ as a brown band (95% yield). ^1^H NMR (CDCl_3_, 300 K, 600 MHz): δ 7.75–7.72 (m, 4H, pyrr, Ph), 7.58 (m, 3H, Ph), 7.43 (s, 1H, platinacycle), 7.38 (m, 5H, Ph), 7.29 (m, 5H, Ph), 7.19 (m, 5H, Ph), 7.15 (m, 2H, Ph), 7.12 (m, 3H, Ph), 7.09–7.06 (m, 4H, pyrr, tell, Ph), 7.00 (m, 2H, tell, Ph), 6.55 (d, ^3^*J*_HH_ = 4.6 Hz, 1H, pyrr), 6.23 (d, ^3^*J*_HH_ = 4.6 Hz, 1H, pyrr); ^13^C NMR (CDCl_3_, 300 K, 150 MHz): δ 178.7 (α-platinacycle), 173.7 (α-pyrr), 171.5 (α-pyrr), 170.4 (β-platinacycle), 158.5 (α-pyrr), 158.1 (α-tell), 153.9 (meso), 151.8 (α-tell), 147.2 (α-pyrr), 147.1 (α-platinacycle), 144.0 (*o*-Ph), 143.3 (ipso), 142.6 (ipso), 142.1 (ipso), 141.9 (ipso), 138.6 (β-platinacycle), 138.5 (β-tell), 137.2 (β-tell), 134.4 (β-pyrr), 133.8 (β-pyrr), 133.6 (ipso), 130.5 (Ph), 130.4 (br, Ph), 130.1 (Ph), 129.8 (Ph), 129.7 (Ph), 129.1 (Ph), 128.9 (β-pyrr, Ph), 128.8 (Ph), 128.6 (Ph), 128.5 (Ph), 128.0 (β-pyrr), 127.9 (Ph), 127.8 (Ph), 127.7 (Ph), 127.6 (Ph), 126.5 (Ph), 124.1 (Ph), 120.4 (Ph), 64.0 (meso), 62.7 (meso); UV–vis (nm, log ε) = 825 (3.6), 739 (3.8), 685 (3.8), 435 (4.3), 350 (4.3); HRMS (ESI) *m*/*z*: 1133.1139, calcd for C_56_H_36_N_2_Cl_2_^195^Pt^130^Te ^+^, [M + H]^+^: 1133.1053. Crystal data for compound **3-Cl**_**2**_: C_56_H_36_Cl_2_N_2_PtTe, C_6_H_14_, *M* = 1216.63, triclinic, *P*1̅, *a* = 11.396(3) Å, *b* = 13.037(3) Å, *c* = 17.490(3) Å, α = 103.78(3), β = 90.38(2)°, γ = 96.21(3),*V* = 2507.5(10) Å^3^, *Z* = 2, *D*_c_ = 1.611 Mg m^–3^, *T* = 100(2) K, *R* = 0.0671, *wR* = 0.1566 [7731 reflections with *I* > 2σ(*I*)] for 658 variables, CCDC 2214349.

#### Reduction of **3-Cl**_**2**_ to **3**

The reaction was performed in an inert atmosphere of a glovebox. 10 mg of **3-Cl**_**2**_ was dissolved in CH_2_Cl_2_ (1 mL) and 50 mg of Zn/Hg was added. The mixture was stirred for 24 h. The resulting product, **3**, was filtered through basic Al_2_O_3_ (95% yield).

## Supporting Information Available

Detailed spectral characterization, DFT-calculated structures coordinates, and simulated UV−vis spectra (PDF)
