# Supplementary material for: Metalation of Tellurophene: Reactivity of 21,23-Ditelluraporphodimethene toward Palladium(II), Platinum(II), and Rhodium(I)
Source: Inorg Chem. 2023 Jan 26;62(7):3056–66. doi: 10.1021/acs.inorgchem.2c03777 (PMC9945301; doi:10.1021/acs.inorgchem.2c03777)
Supplement: Supplementary file 1 — ic2c03777_si_001.pdf [file ic2c03777_si_001.pdf]

Supporting Information

Metalation of Tellurophene: Reactivity of 21,23-Ditelluraporphodimethene toward Palladium(II), Platinum(II), and Rhodium(I)

Grzegorz Vetter, Agata Białońska, and Ewa Pacholska-Dudziak*

Department of Chemistry, University of Wroclaw, 50-383 Wrocław, Poland

E-mail: ewa.dudziak@uwr.edu.pl

Table of Contents

Figure S1. ^{1}H NMR spectrum of **1a**; 500 MHz, $CDCl_3$, 300 K 4

Figure S2. ^{1}H NMR spectrum of **1a**; 600 MHz, C_6D_6, 300 K 4

Figure S3. ^{13}C NMR spectrum of **1a**; 150 MHz, C_6D_6, 300 K 5

Figure S4. HRMS ESI (+MS) spectra of **1a**: measured (top) and simulated (bottom) calc. for $C_{56}H_{38}N_2Te_2$, $[M+H]^+$ 6

Figure S5. ^{13}C NMR spectrum of **1b**; 125 MHz, $CDCl_3$, 300 K 7

Figure S6. HRMS ESI (+MS) spectra of **1b**: measured (top) and simulated (bottom) calc. for $C_{58}H_{42}N_2O_2Te_2$, $[M+H]^+$ 8

Figure S7. ^{13}C NMR spectrum of **1-PdCl₂**; 150 MHz, $CDCl_3$, 300 K 9

Figure S8. HRMS ESI (+MS) spectra of **1-PdCl₂**: measured (top) and simulated (bottom) calc. for $C_{56}H_{38}ClN_2PdTe_2$, $[M-Cl]^+$ 10

Figure S9. ^{1}H NMR spectrum of **1-PtCl₂**; 500 MHz, $CDCl_3$, 300 K 10

Figure S10. ^{13}C NMR spectrum of **2**; 150 MHz, $CDCl_3$, 300 K 11

Figure S11. HRMS ESI (+MS) spectra of **2**: measured (top) and simulated (bottom) calc. for $C_{58}H_{43}Cl_2N_2O_2RhTe$, $[M+H]^+$ 12

Figure S12. ^{13}C NMR spectrum of **3**; 150 MHz, $CDCl_3$, 300 K 13

Figure S13. HRMS ESI (+MS) spectra of **3**: measured (top) and simulated (bottom) calc. for $C_{56}H_{36}N_2PtTe$, $[M+H]^+$ 14

Figure S14. ^{13}C NMR spectrum of **3-Cl₂**; 150 MHz, $CDCl_3$, 300 K 15

Figure S15. HRMS ESI (+MS) spectra of **3-Cl₂**: measured (top) and simulated (bottom) calc. for $C_{56}H_{36}Cl_2N_2PtTe$, $[M+H]^+$ 16

Table S1. Selected interplanar angles for X-ray structures of **1b**, **1-PdCl₂**, **2**, **3**, and **3-Cl₂** ... 16

Table S2. Energies of DFT calculated molecular orbitals (eV) for **1a**, **1b**, **1-PdCl₂**, **2**, **3**, and **3-Cl₂** 17

Figure S16. Energy graph of DFT calculated molecular orbitals (HOMO-10 to LUMO+4) for **1a**, **1b**, **1-PdCl₂**, **2**, **3**, and **3-Cl₂**. 17

Figure S17. UV-Vis electronic spectrum of **1a** (CH_2Cl_2, 298 K). 18

Figure S18. Simulated electronic spectrum (blue trace) and a histogram of electronic transitions (green sticks) for **1a**. 18

Table S3. UV-Vis transitions (oscillator strength > 0.01) calculated with TD-DFT for **1a** ... 18

Figure S19. UV-Vis electronic spectrum of **1b** (CH_2Cl_2, 298 K). 20

Figure S20. Simulated electronic spectrum (blue trace) and a histogram of electronic transitions (green sticks) for **1b** 20

Table S4. UV-Vis transitions (oscillator strength > 0.01) calculated with TD-DFT for **1b** .. 20

Figure S21. UV-Vis electronic spectrum of **1-PdCl₂** (CH_2Cl_2, 298 K). 22

Figure S22. Simulated electronic spectrum (blue trace) and a histogram of electronic transitions (green sticks) for **1-PdCl₂**.. 22

Table S5. UV-Vis transitions (oscillator strength > 0.01) calculated with TD-DFT for **1-PdCl2** 22

Figure S23. UV-Vis electronic spectrum of **2** (CH_2Cl_2, 298 K). 24

Figure S24. Simulated electronic spectrum (blue trace) and a histogram of electronic transitions (green sticks) for **2**.. 24

Table S6. UV-Vis transitions (oscillator strength > 0.01) calculated with TD-DFT for **2** 24

Figure S25. UV-Vis electronic spectrum of **3** (CH_2Cl_2, 298 K). 26

Figure S26. Simulated electronic spectrum (blue trace) and a histogram of electronic transitions (green sticks) for **3**.. 26

Table S7. UV-Vis transitions calculated with TD-DFT for **3** 26

Figure S27. UV-Vis electronic spectrum of **3-Cl₂** (CH_2Cl_2, 298 K)...................................... 28

Figure S28. Simulated electronic spectrum (blue trace) and a histogram of electronic transitions (green sticks) for **3-Cl₂** 28

Table S8. UV-Vis transitions (oscillator strength > 0.01) calculated with TD-DFT for **3-Cl₂** 28

Table S9. Cartesian coordinates for **1a**, **1b**, **1-PdCl₂**, **2**, **2-1**, **3**, **3-1**, **3-2**, and **3-Cl₂** (B3PW91/SDD for Rh, Pt and Te, and 6-31G(d,p) for the rest of atoms)................................30

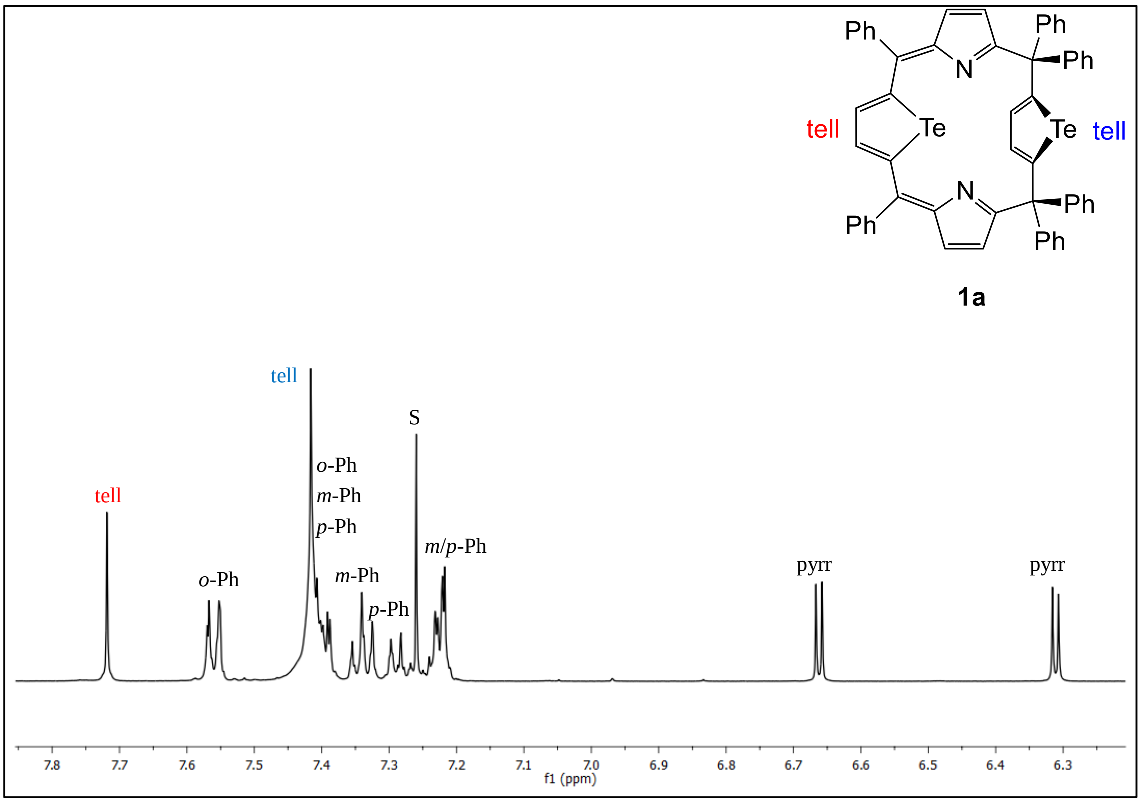

Figure S1. ^{1}H NMR spectrum of **1a**; 500 MHz, $CDCl_3$, 300 K.

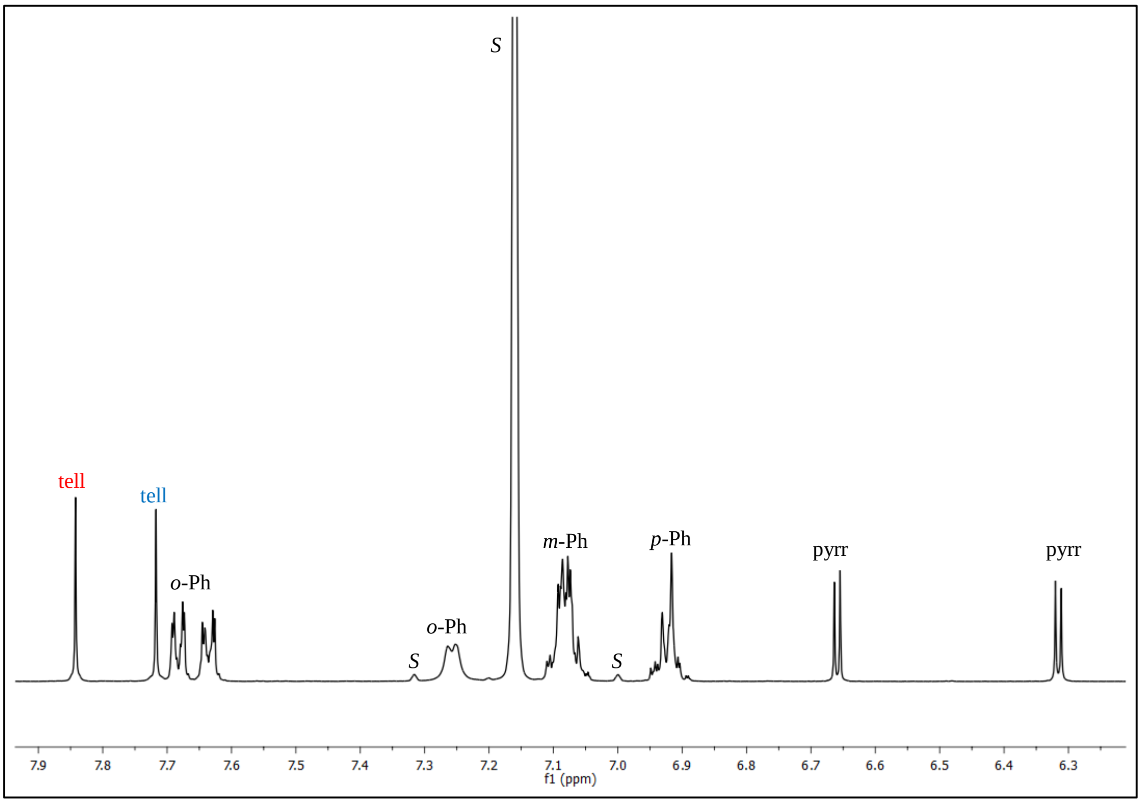

Figure S2. ^{1}H NMR spectrum of **1a**; 600 MHz, C_6D_6, 300 K.

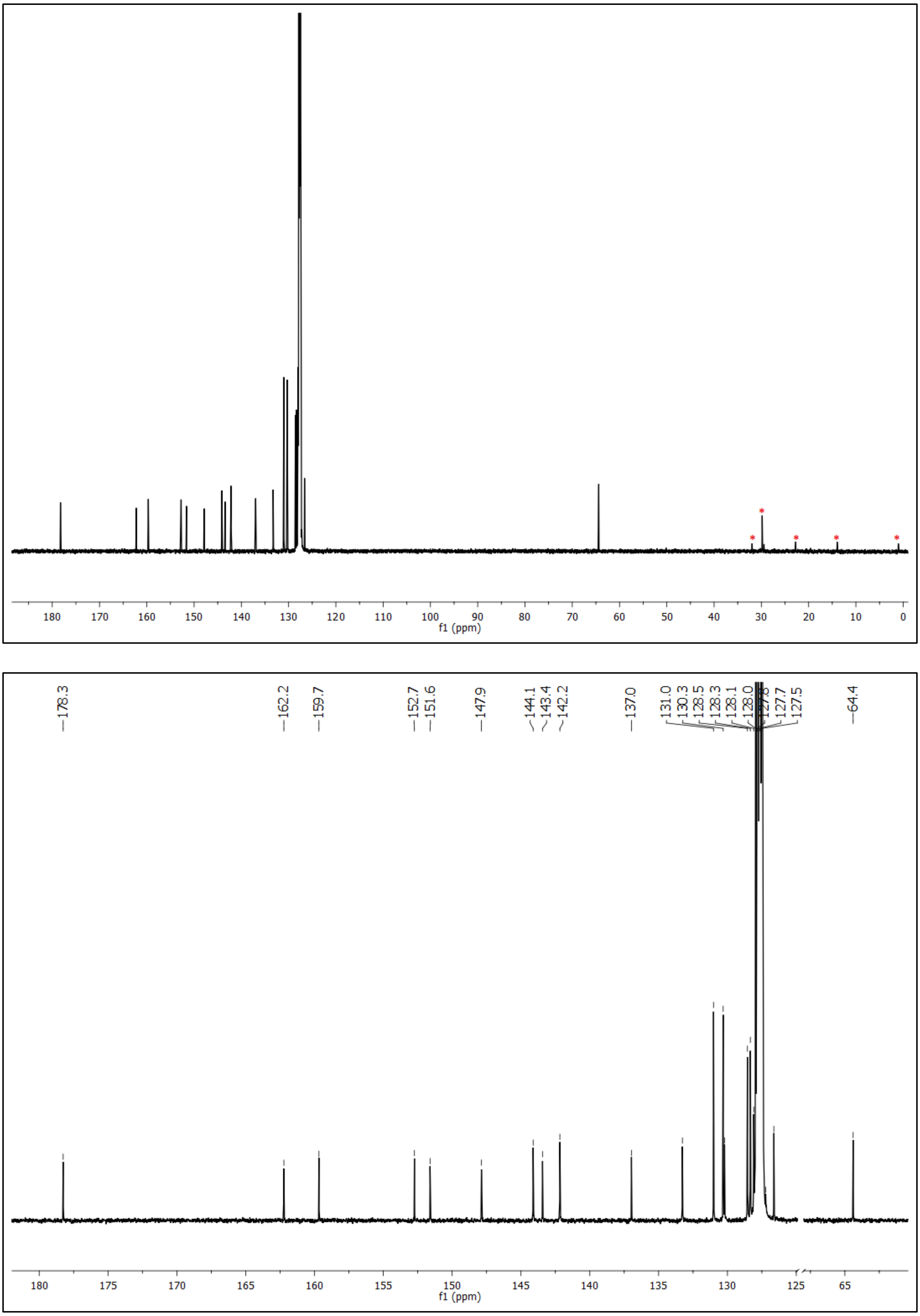

Figure S3. ^{13}C NMR spectrum of **1a**; 150 MHz, C_6D_6, 300 K (top: the whole spectral range, bottom: the most informative region; * = impurities).

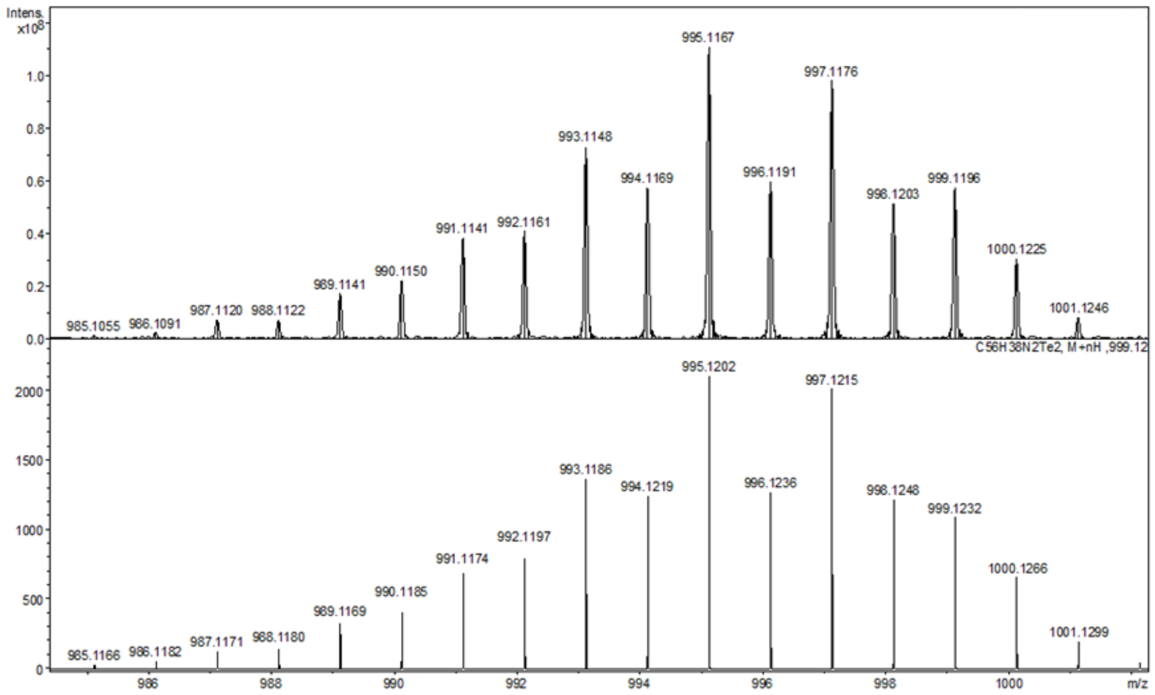

Figure S4. HRMS ESI (+MS) spectra of **1a**: measured (top) and simulated (bottom) calc. for $C_{56}H_{38}N_2Te_2$, $[M+H]^+$.

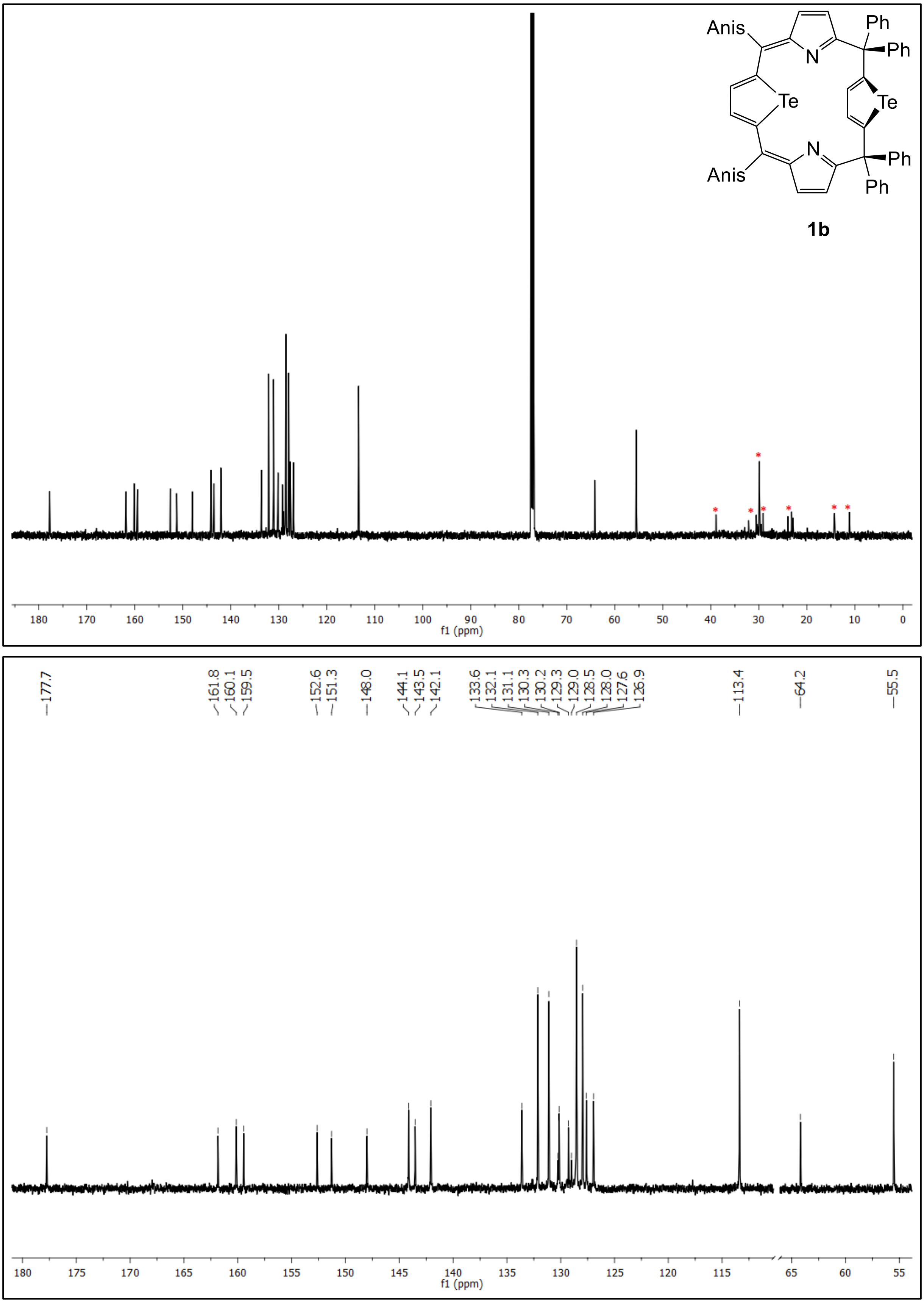

Figure S5. ^{13}C NMR spectrum of **1b**; 125 MHz, $CDCl_3$, 300 K (top: the whole spectral range, bottom: the most informative region; * = impurities). Anis = 4-methoxyphenyl.

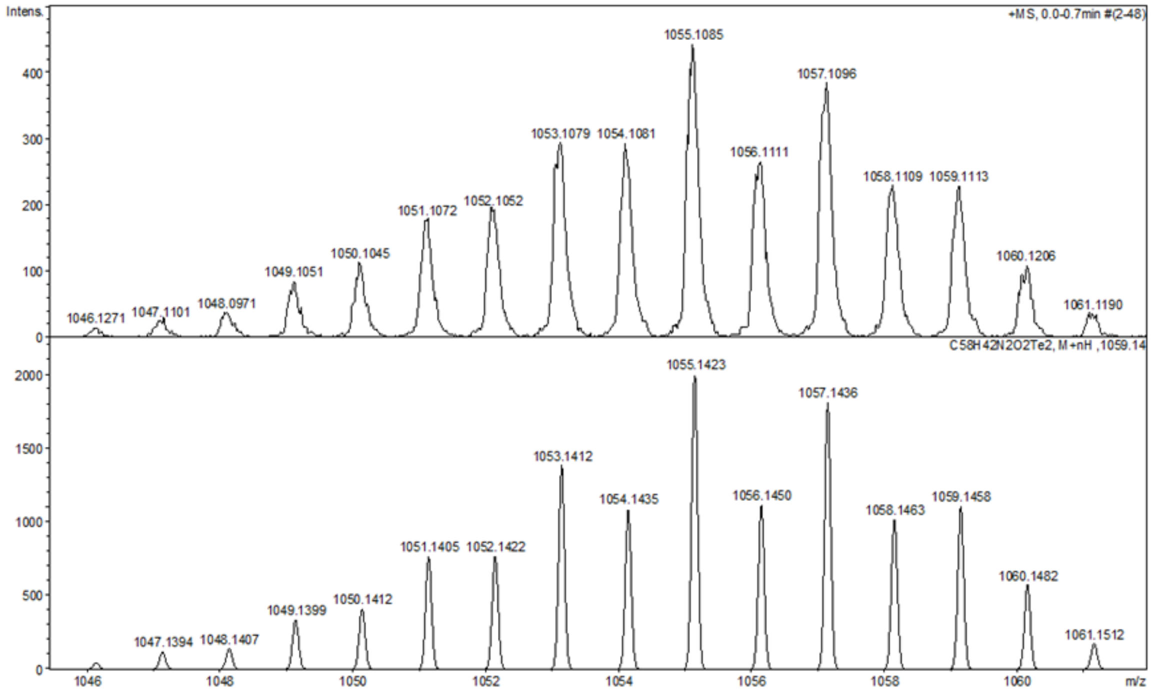

Figure S6. HRMS ESI (+MS) spectra of **1b**: measured (top) and simulated (bottom) calc. for $C_{58}H_{42}N_2O_2Te_2$, $[M+H]^+$.

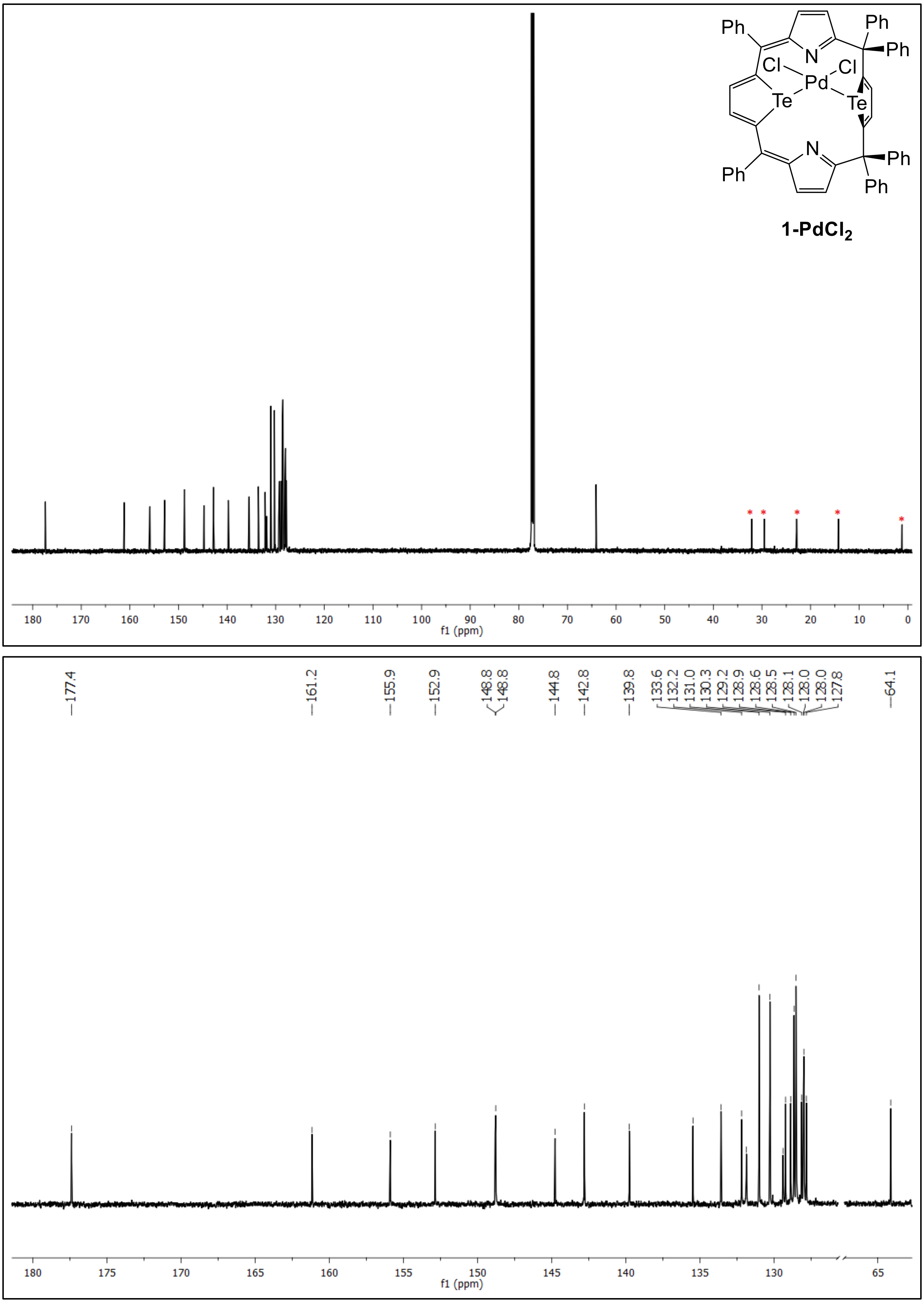

Figure S7. ^{13}C NMR spectrum of **1-PdCl$_2$**; 150 MHz, $CDCl_3$, 300 K (top: the whole spectral range, bottom: the most informative region; * = impurities).

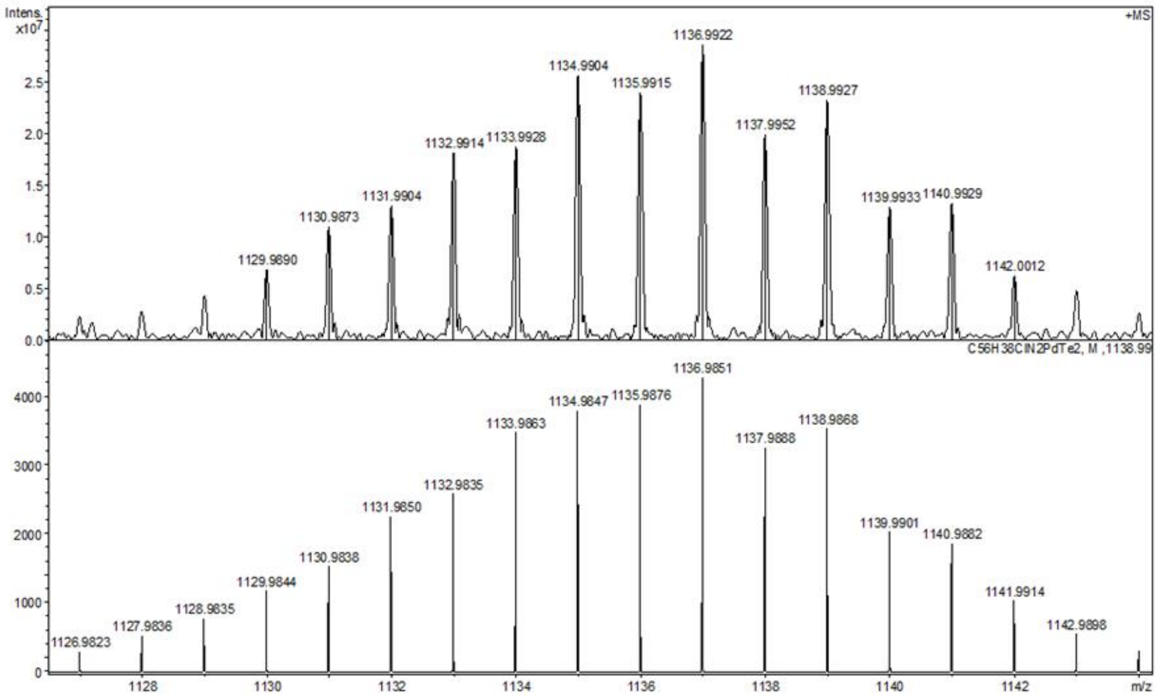

Figure S8. HRMS ESI (+MS) spectra of **1-PdCl₂**: measured (top) and simulated (bottom) calc. for $C_{56}H_{38}ClN_2PdTe_2$, $[M-Cl]^+$.

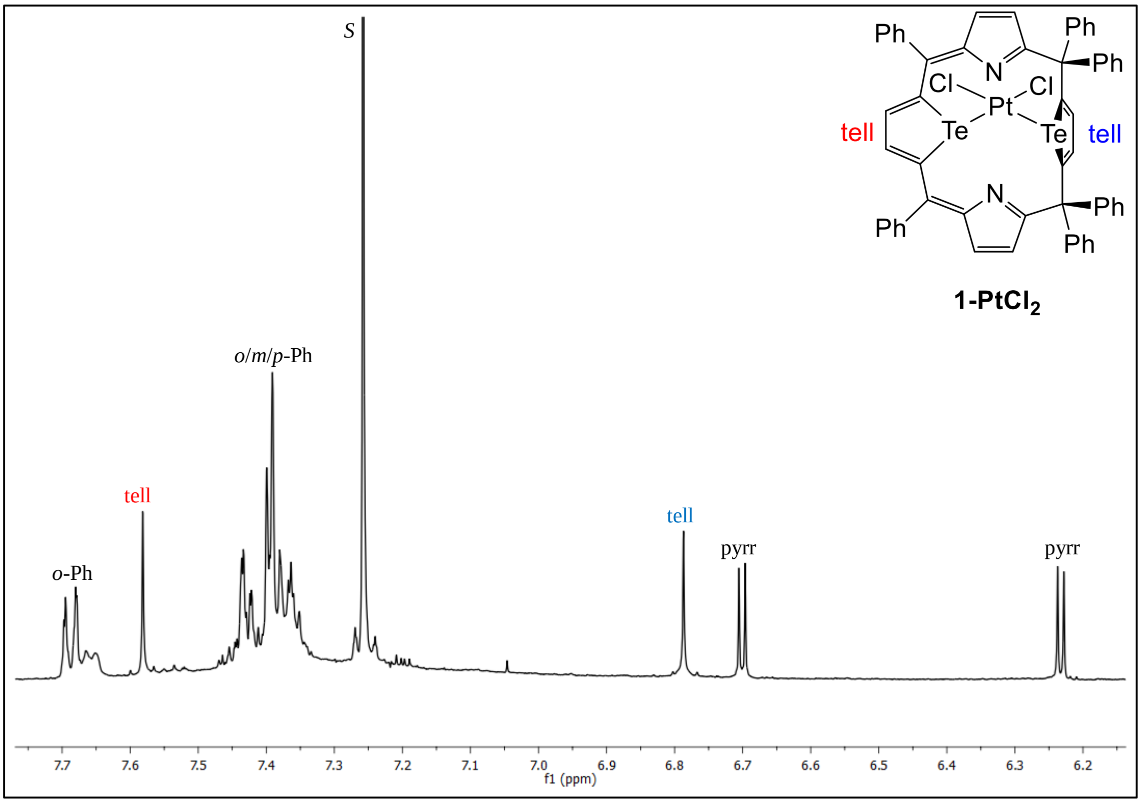

Figure S9. ^{1}H NMR spectrum of **1-PtCl₂**; 500 MHz, $CDCl_3$, 300 K.

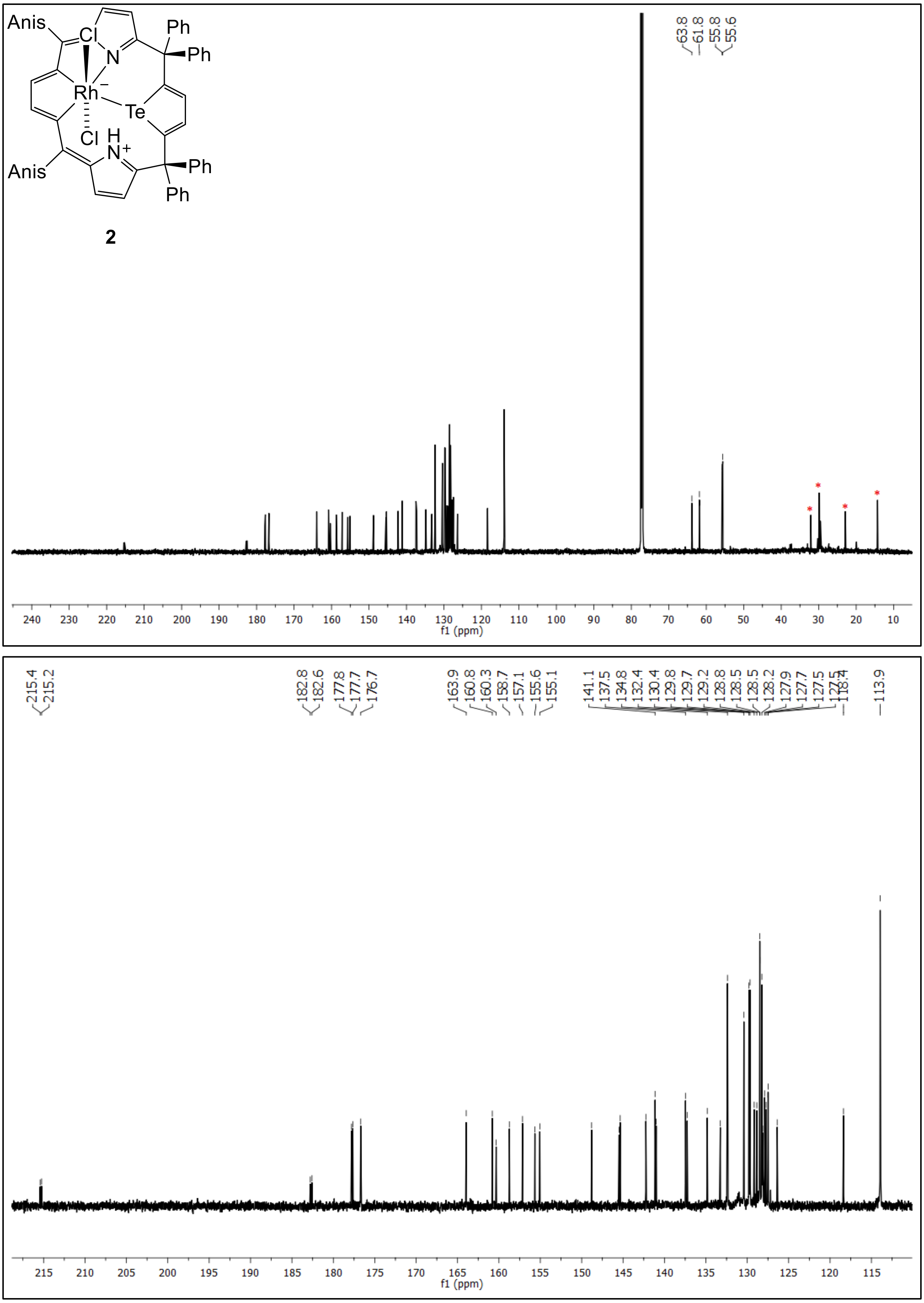

Figure S10. ^{13}C NMR spectrum of **2**; 150 MHz, $CDCl_3$, 300 K (top: the whole spectral range, bottom: the most informative region; * = impurities).

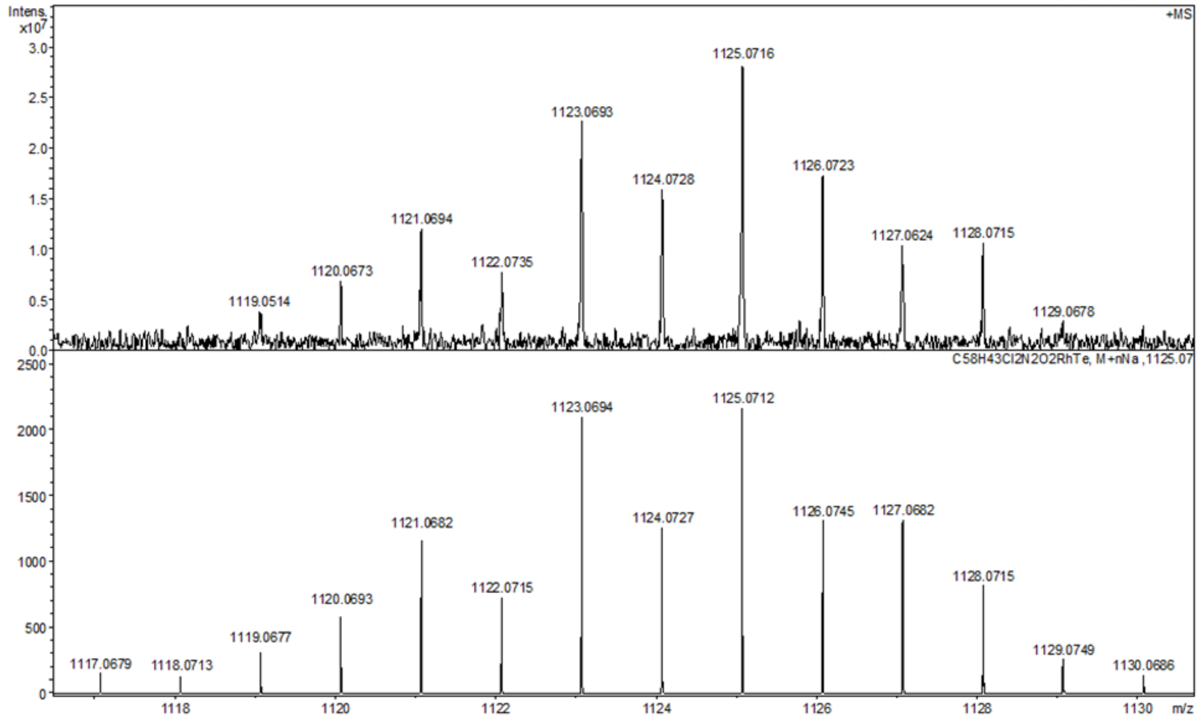

Figure S11. HRMS ESI (+MS) spectra of **2**: measured (top) and simulated (bottom) calc. for $C_{58}H_{43}Cl_2N_2O_2RhTe$, $[M+Na]^+$.

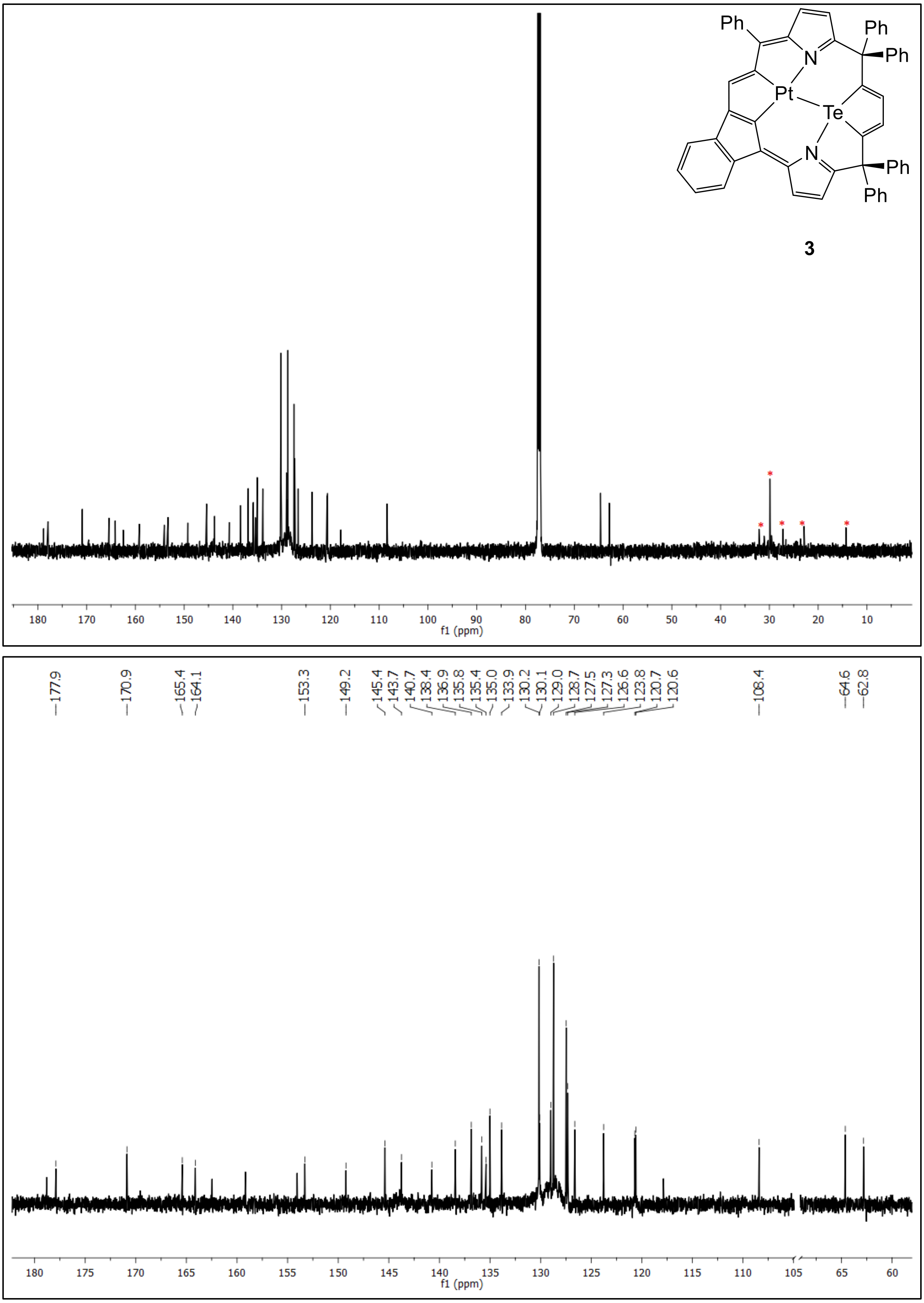

Figure S12. ^{13}C NMR spectrum of **3**; 150 MHz, $CDCl_3$, 300 K (top: the whole spectral range, bottom: the most informative region; * = impurities).

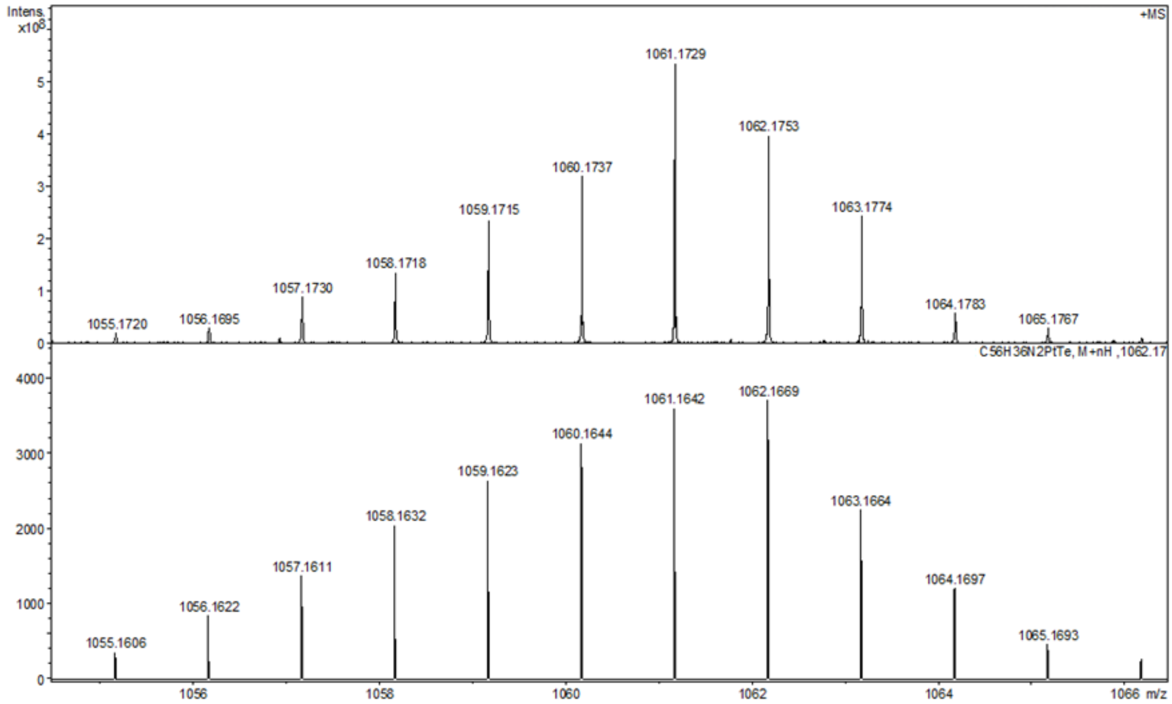

Figure S13. HRMS ESI (+MS) spectra of **3**: measured (top) and simulated (bottom) calc. for $C_{56}H_{36}N_2PtTe$, $[M+H]^+$.

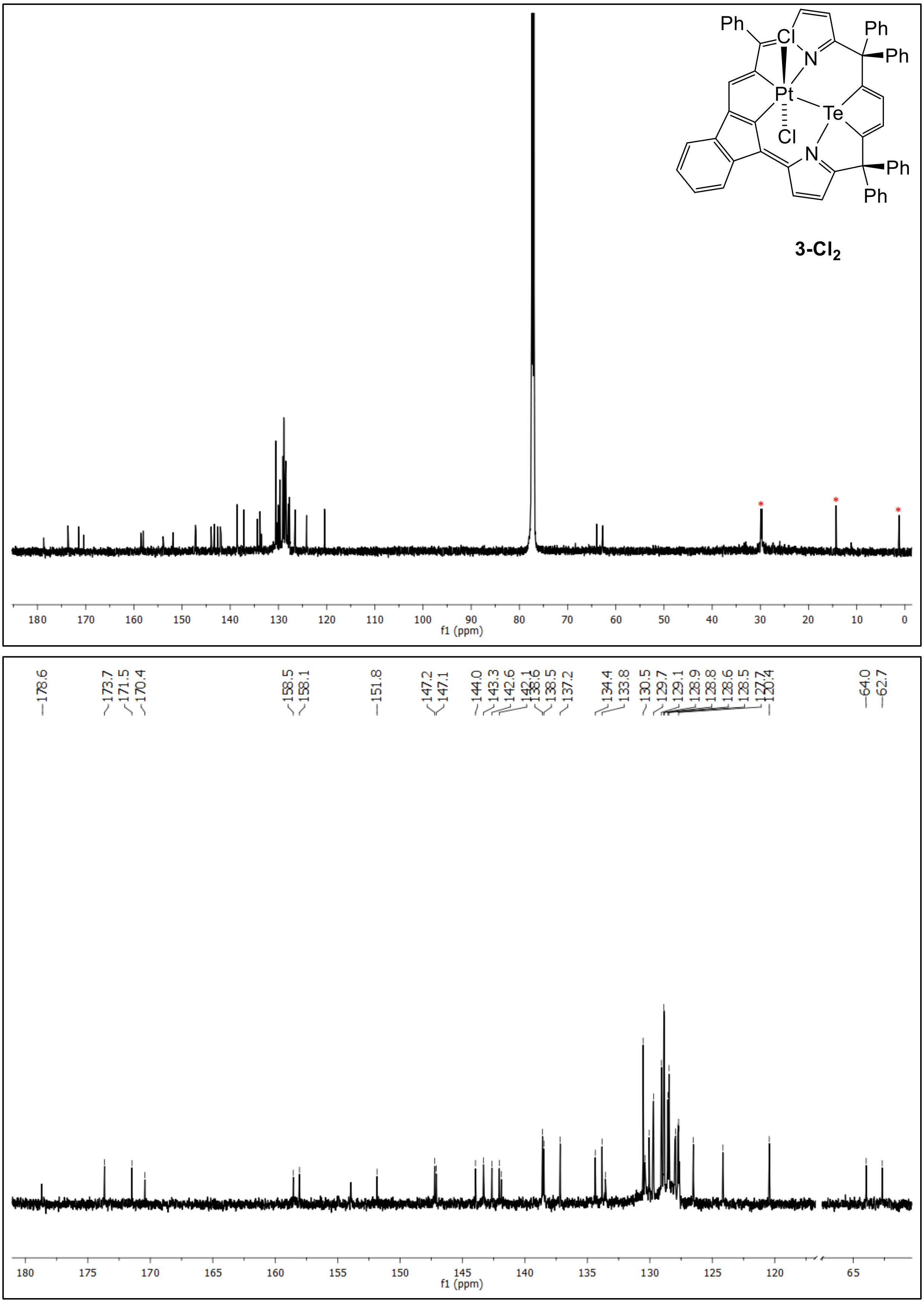

Figure S14. ^{13}C NMR spectrum of **3-Cl$_2$**; 150 MHz, $CDCl_3$, 300 K (top: the whole spectral range, bottom: the most informative region; * = impurities).

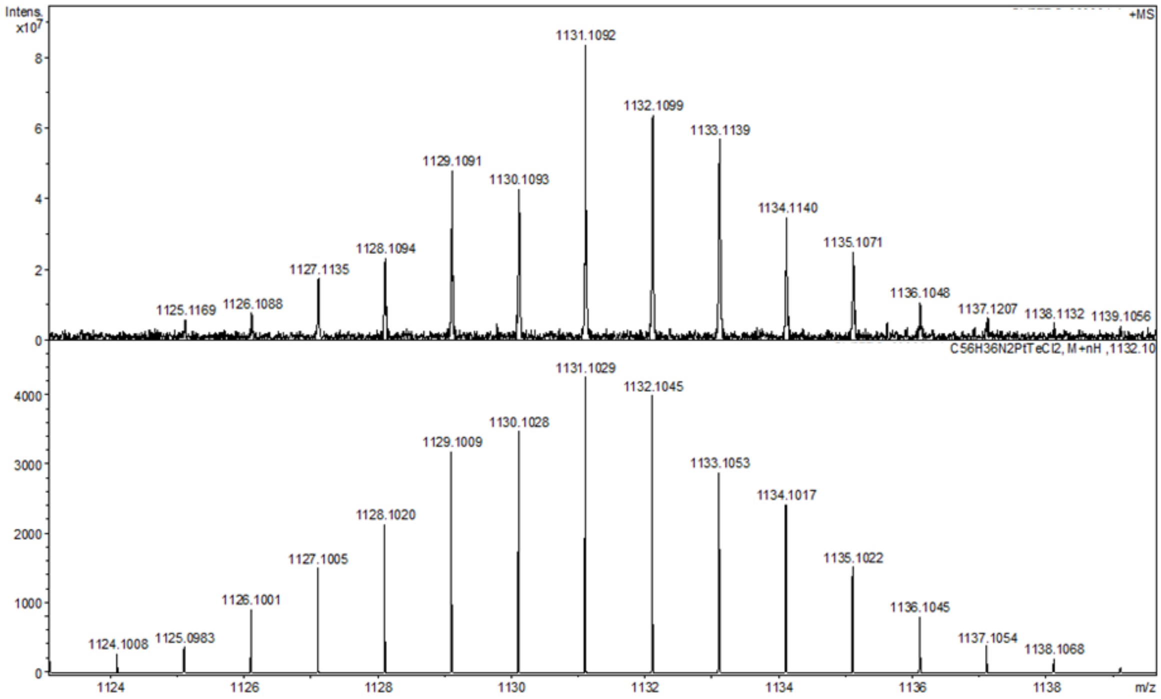

Figure S15. HRMS ESI (+MS) spectra of **3-Cl$_2$**: measured (top) and simulated (bottom) calc. for $C_{56}H_{36}Cl_2N_2PtTe$, $[M+H]^+$.

Table S1. Selected interplanar angles for X-ray structures of **1b**, **1-PdCl$_2$**, **2**, **3**, and **3-Cl$_2$**.

	**1b**	**1-PdCl$_2$**	**2**	**3**	**3-Cl$_2$**
PdL_4 – $C_{4\text{-}meso}$ plane		88.99(6)°			
tellurophene-Te23 plane – $C_{4\text{-}meso}$ plane	110.67(14)°	88.13(15)°			
tellurophene-Te23 plane – MC1C4N22Te plane		89.77° M = Pd	111.31(10)° M = Rh	130.30° M = Pt	117.31° M = Pt

Table S2. Energies of DFT calculated molecular orbitals (eV) for **1a**, **1b**, **1-PdCl$_2$**, **2**, **3**, and **3-Cl$_2$**.

MO	1a	1b	1-PdCl$_2$	2	3	3-Cl$_2$
LUMO+4	-0,53	-0,49	-1,31	-0,94	-0,55	-0,82
LUMO+3	-0,67	-0,63	-1,32	-1,13	-1,06	-1,56
LUMO+2	-0,73	-0,7	-2,25	-1,54	-1,25	-2,42
LUMO+1	-1,94	-1,87	-2,93	-2,13	-2,24	-2,54
LUMO	-3,2	-3,13	-3,56	-3,5	-3,42	-3,66
HOMO	-5,59	-5,53	-5,92	-5,5	-5,1	-5,62
HOMO-1	-5,68	-5,63	-6,42	-6,06	-6,08	-6,23
HOMO-2	-5,86	-5,82	-6,58	-6,08	-6,12	-6,25
HOMO-3	-6,19	-6,12	-6,59	-6,12	-6,18	-6,56
HOMO-4	-6,58	-6,15	-6,66	-6,24	-6,3	-6,72
HOMO-5	-6,61	-6,22	-6,72	-6,35	-6,5	-6,91
HOMO-6	-6,72	-6,57	-6,73	-6,4	-6,6	-6,92
HOMO-7	-6,81	-6,67	-6,77	-6,62	-6,7	-6,93
HOMO-8	-6,88	-6,75	-7,01	-6,72	-6,83	-7
HOMO-9	-6,9	-6,81	-7,03	-6,86	-6,88	-7,05
HOMO-10	-6,93	-6,87	-7,05	-6,97	-6,91	-7,06

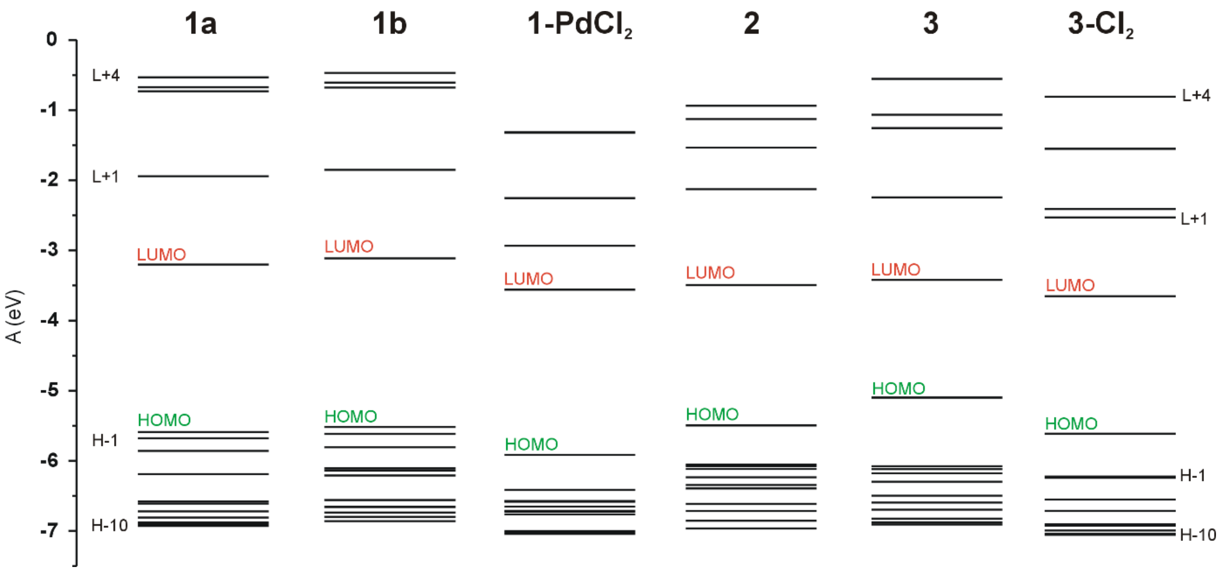

Figure S16. Energy graph of DFT calculated molecular orbitals (HOMO-10 to LUMO+4) for **1a**, **1b**, **1-PdCl$_2$**, **2**, **3**, and **3-Cl$_2$**.

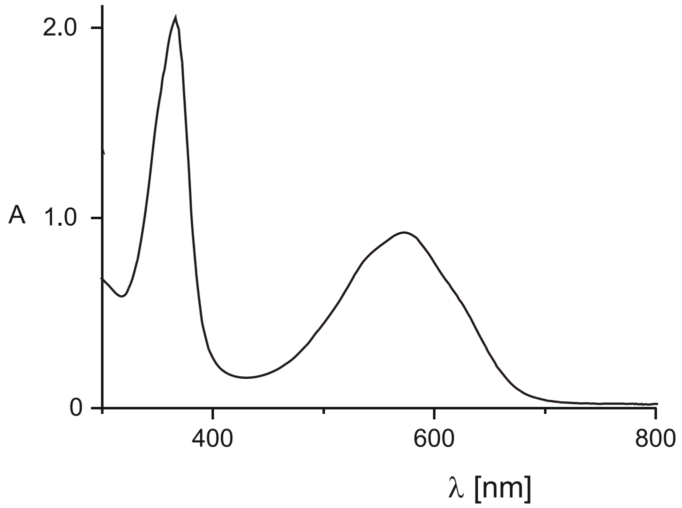

Figure S17. UV-Vis electronic spectrum of **1a** (CH_2Cl_2, 298 K).

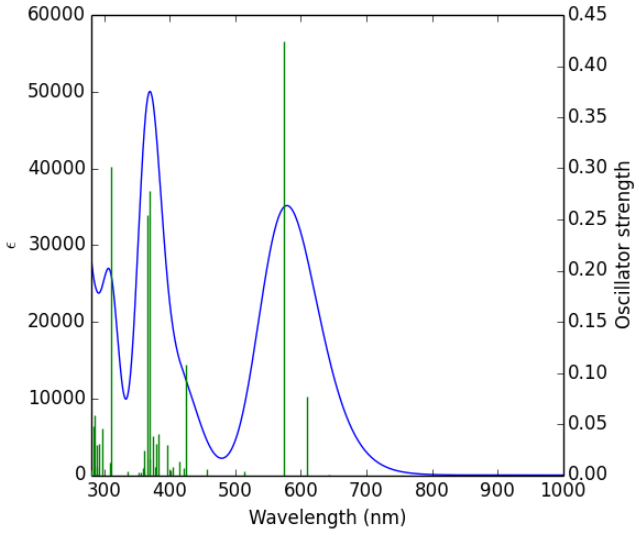

Figure S18. Simulated electronic spectrum (blue trace) and a histogram of electronic transitions (green sticks) for **1a** (num. pts.: 500; FWHM: 3000 cm^{-1}).

Table S3. UV-Vis transitions (oscillator strength > 0.01) calculated with TD-DFT for **1a**.

No.	Energy (cm^{-1})	Wavelength (nm)	Osc. Strength	Major contribs
1	15534.35	643.7349	0.0009	H-1->LUMO (99%)
2	16407.04	609.4943	0.0774	H-2->LUMO (71%), HOMO->LUMO (29%)
3	17397.50	574.7953	0.4244	H-2->LUMO (28%), HOMO->LUMO (70%)
4	19481.65	513.3035	0.0038	H-3->LUMO (98%)
5	21882.78	456.9803	0.0057	H-6->LUMO (49%), H-5->LUMO (13%), H-4->LUMO (20%)
6	23533.00	424.9352	0.1084	H-5->LUMO (25%), H-4->LUMO (52%), HOMO->L+1 (17%)
7	23718.51	421.6116	0.0069	H-7->LUMO (19%), H-6->LUMO (29%), H-5->LUMO (31%), H-4->LUMO (12%)
8	24081.46	415.2572	0.0133	H-7->LUMO (65%)
9	24712.19	404.6586	0.0079	H-8->LUMO (49%)
10	24916.25	401.3445	0.0045	H-1->L+1 (80%)
11	25203.39	396.7721	0.0296	H-19->LUMO (11%), H-13->LUMO (19%), H-11->LUMO (23%), H-10->LUMO (10%), H-9->LUMO (19%)

12	26065.60	383.6474	0.0405	H-12->LUMO (14%), H-10->LUMO (22%), H-9->LUMO (39%)
13	26322.09	379.9091	0.0303	H-13->LUMO (15%), H-12->LUMO (34%), H-8->LUMO (21%)
14	26514.85	377.1471	0.0086	H-12->LUMO (10%), H-11->LUMO (54%), H-10->LUMO (10%), H-9->LUMO (12%)
15	26663.26	375.0479	0.0381	H-12->LUMO (27%), H-11->LUMO (10%), H-10->LUMO (35%)
16	27007.66	370.2653	0.278	H-8->LUMO (10%), H-2->L+1 (29%), HOMO->L+1 (30%)
17	27121.39	368.7127	0.0159	H-19->LUMO (17%), H-14->LUMO (12%), H-13->LUMO (34%), H-2->L+1 (13%)
18	27373.84	365.3123	0.2545	H-2->L+1 (46%), HOMO->L+1 (19%)
19	27631.94	361.9000	0.003	H-17->LUMO (33%), H-16->LUMO (13%), H-14->LUMO (37%)
20	27705.34	360.9413	0.024	H-18->LUMO (14%), H-17->LUMO (25%), H-16->LUMO (24%), H-14->LUMO (26%)
21	27784.38	359.9145	0.0074	H-18->LUMO (27%), H-17->LUMO (19%), H-16->LUMO (46%)
22	28039.25	356.6429	0.0025	H-18->LUMO (24%), H-15->LUMO (65%)
23	28357.04	352.6462	0.0024	H-19->LUMO (31%), H-18->LUMO (22%), H-17->LUMO (15%), H-15->LUMO (10%)
24	29771.74	335.8890	0.0035	H-3->L+1 (95%)
25	32175.29	310.7975	0.3015	H-20->LUMO (77%)
26	32431.78	308.3396	0.0125	H-6->L+1 (48%), H-5->L+1 (10%), H-4->L+1 (21%)
27	33732.76	296.4477	0.0454	H-5->L+1 (44%), H-4->L+1 (42%)
28	34132.81	292.97325	0.031	H-23->LUMO (13%), H-6->L+1 (22%), H-5->L+1 (10%), H-4->L+1 (23%)
29	34482.05	290.0059	0.0005	H-1->L+2 (18%), H-1->L+3 (40%), HOMO->L+2 (13%)
30	34636.91	288.7093	0.0076	HOMO->L+2 (57%)
31	34669.18	288.4407	0.0297	H-23->LUMO (10%), H-21->LUMO (10%), H-5->L+1 (17%), H-2->L+3 (14%)
32	34787.74	287.45767	0.0009	H-2->L+2 (10%), H-2->L+3 (27%), HOMO->L+2 (15%), HOMO->L+3 (14%)
33	35044.23	285.3537	0.0585	H-7->L+1 (15%), H-1->L+2 (48%), H-1->L+3 (25%)
34	35195.05	284.1308	0.0474	H-7->L+1 (72%)
35	35644.31	280.5497	0.0044	H-21->LUMO (13%), H-10->L+1 (13%), H-9->L+1 (19%), H-8->L+1 (29%), H-6->L+1 (12%)
36	35854.01	278.9088	0.0081	H-2->L+3 (13%), HOMO->L+3 (72%)
37	35916.92	278.4203	0.0268	H-21->LUMO (29%), H-13->L+1 (16%), H-11->L+1 (20%), H-9->L+1 (16%)
38	36178.25	276.4092	0.0351	H-21->LUMO (11%), HOMO->L+4 (44%), HOMO->L+5 (12%)
39	36258.90	275.7943	0.0213	H-23->LUMO (17%), H-21->LUMO (15%), HOMO->L+4 (23%)
40	36723.48	272.3053	0.003	H-23->LUMO (13%), H-10->L+1 (34%), H-9->L+1 (24%)
41	36808.17	271.6788	0.0046	H-13->L+1 (17%), H-12->L+1 (30%), H-9->L+1 (14%), H-8->L+1 (25%)
42	36967.06	270.5111	0.004	H-1->L+4 (15%), HOMO->L+4 (14%), HOMO->L+5 (52%)
43	37046.91	269.9280	0.0014	H-12->L+1 (25%), H-11->L+1 (35%), H-10->L+1 (22%)
44	37187.26	268.9093	0.0042	H-13->L+1 (30%), H-12->L+1 (15%), H-11->L+1 (12%), H-10->L+1 (10%)
45	37298.56	268.1069	0.0126	H-2->L+2 (15%), H-1->L+4 (13%), H-1->L+5 (32%)
46	37380.02	267.5226	0.0218	H-1->L+4 (41%), H-1->L+5 (13%), HOMO->L+6 (18%)
47	37529.24	266.4589	0.2365	H-22->LUMO (15%), H-2->L+2 (38%), H-2->L+3 (11%), H-1->L+5 (11%)
48	37684.90	265.3583	0.0011	H-16->L+1 (27%), H-14->L+1 (19%)
49	37760.72	264.8255	0.0345	H-19->L+1 (16%), H-18->L+1 (18%)
50	37848.63	264.2103	0.0332	H-17->L+1 (19%), H-16->L+1 (26%), HOMO->L+6 (19%), HOMO->L+7 (15%)
51	37934.94	263.6092	0.0054	H-22->LUMO (28%), H-18->L+1 (16%)
52	38018.01	263.0332	0.0064	H-2->L+4 (27%), H-2->L+5 (40%)
53	38040.60	262.8771	0.0387	H-22->LUMO (11%), H-18->L+1 (11%), H-17->L+1 (37%), HOMO->L+6 (11%)
54	38138.19	262.2044	0.0048	HOMO->L+7 (14%), HOMO->L+8 (56%)
55	38194.65	261.8168	0.0055	H-16->L+1 (12%), H-14->L+1 (45%), HOMO->L+7 (14%)
56	38338.22	260.8363	0.009	HOMO->L+7 (26%), HOMO->L+8 (13%)
57	38483.40	259.8523	0.0013	H-1->L+6 (43%), H-1->L+7 (21%)
58	38526.95	259.5586	0.0016	H-18->L+1 (17%), H-15->L+1 (49%)
59	38618.09	258.9460	0.0274	HOMO->L+9 (59%)
60	38840.70	257.4619	0.0119	H-19->L+1 (12%), H-3->L+2 (26%), H-3->L+3 (18%)

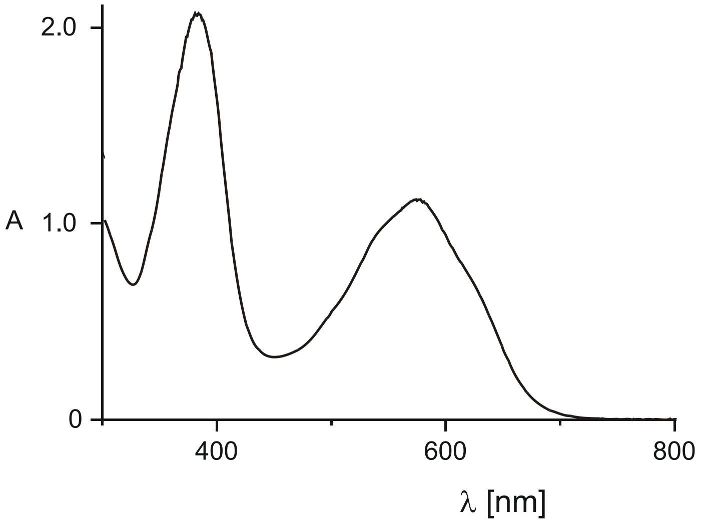

Figure S19. UV-Vis electronic spectrum of **1b** (CH_2Cl_2, 298 K).

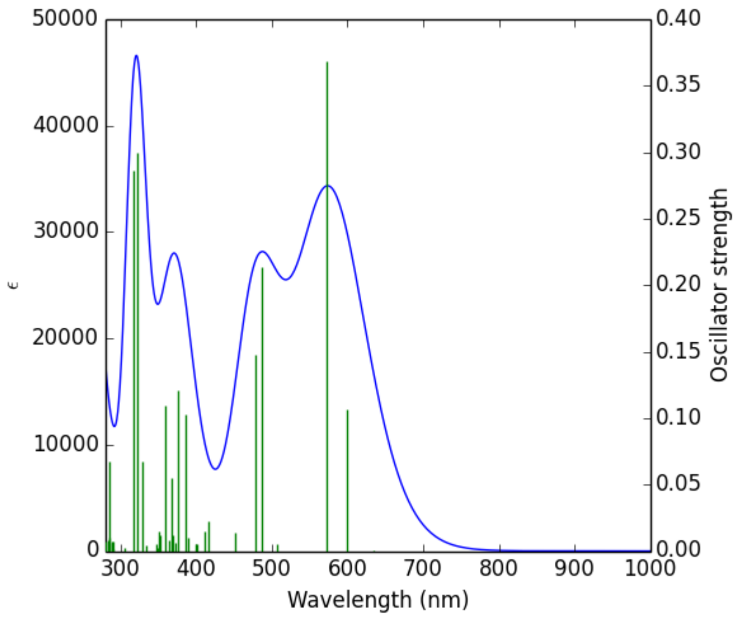

Figure S20. Simulated electronic spectrum (blue trace) and a histogram of electronic transitions (green sticks) for **1b** (num. pts.: 500; FWHM: 3000 cm^{-1}).

Table S4. UV-Vis transitions (oscillator strength > 0.01) calculated with TD-DFT for **1b**.

No.	Energy (cm^{-1})	Wavelength (nm)	Osc. Strength	Major contribs
1	15748.89	634.9654	0.0001	H-1->LUMO (98%)
2	16690.95	599.1270	0.1067	H-2->LUMO (67%), HOMO->LUMO (32%)
3	17470.90	572.3805	0.3683	H-2->LUMO (32%), HOMO->LUMO (66%)
4	19712.33	507.2968	0.0048	H-4->LUMO (75%), H-3->LUMO (24%)
5	20534.21	486.9922	0.2135	H-4->LUMO (20%), H-3->LUMO (70%)
6	20883.45	478.8480	0.1479	H-5->LUMO (94%)
7	22157.82	451.3080	0.0143	H-7->LUMO (44%), H-6->LUMO (37%)
8	24031.46	416.1213	0.0224	H-8->LUMO (40%), H-7->LUMO (28%), H-6->LUMO (24%)
9	24285.52	411.7680	0.0151	H-8->LUMO (40%), H-7->LUMO (16%), H-6->LUMO (25%)
10	24917.86	401.3185	0.0055	H-9->LUMO (38%), H-1->L+1 (15%)
11	25065.47	398.9553	0.0049	H-9->LUMO (12%), H-1->L+1 (64%)
12	25632.48	390.1301	0.0103	H-19->LUMO (21%), H-13->LUMO (14%), H-11->LUMO (25%), H-1->L+1 (10%)

13	25909.93	385.9524	0.1027	H-10->LUMO (19%), HOMO->L+1 (45%)
14	26607.61	375.8324	0.1211	H-10->LUMO (44%), H-9->LUMO (17%), HOMO->L+1 (18%)
15	26780.21	373.4100	0.0061	H-13->LUMO (17%), H-12->LUMO (32%), H-11->LUMO (19%)
16	27043.96	369.7684	0.0122	H-13->LUMO (52%), H-11->LUMO (40%)
17	27237.53	367.1405	0.0546	H-19->LUMO (10%), H-14->LUMO (15%), H-12->LUMO (44%)
18	27432.72	364.5282	0.0084	H-2->L+1 (82%)
19	27886.00	358.6028	0.1097	H-14->LUMO (64%)
20	28403.82	352.0654	0.0119	H-16->LUMO (25%), H-15->LUMO (68%)
21	28507.86	350.7804	0.0145	H-20->LUMO (10%), H-18->LUMO (11%), H-17->LUMO (69%)
22	28661.92	348.8950	0.0027	H-19->LUMO (11%), H-18->LUMO (43%), H-16->LUMO (28%)
23	28744.19	347.8964	0.0052	H-19->LUMO (25%), H-18->LUMO (42%), H-17->LUMO (12%), H-16->LUMO (14%)
24	29990.32	333.4409	0.0041	H-4->L+1 (68%), H-3->L+1 (28%)
25	30333.11	329.6728	0.0678	H-4->L+1 (26%), H-3->L+1 (69%)
26	31009.01	322.4870	0.3001	H-20->LUMO (15%), H-5->L+1 (79%)
27	31509.88	317.3608	0.2861	H-20->LUMO (52%), H-5->L+1 (18%), HOMO->L+1 (10%)
28	32651.16	306.2678	0.0023	H-7->L+1 (44%), H-6->L+1 (36%)
29	34391.72	290.7677	0.0017	H-1->L+2 (15%), H-1->L+3 (15%), HOMO->L+2 (39%)
30	34445.76	290.3115	0.0071	H-23->LUMO (13%), H-7->L+1 (18%), H-6->L+1 (18%)
31	34521.57	289.6739	0.0074	H-1->L+2 (14%), H-1->L+3 (15%), HOMO->L+2 (49%)
32	34696.60	288.2127	0.0062	H-2->L+2 (18%), H-2->L+3 (37%), H-1->L+2 (14%), HOMO->L+3 (16%)
33	34924.85	286.3290	0.0101	H-23->LUMO (16%), H-21->LUMO (12%), H-7->L+1 (15%), H-6->L+1 (30%)
34	34979.70	285.8801	0.0671	H-1->L+2 (42%), H-1->L+3 (35%)
35	35315.23	283.1640	0.0085	H-8->L+1 (84%)
36	35634.63	280.6259	0.0076	H-2->L+3 (11%), HOMO->L+3 (71%)
37	35795.13	279.3676	0.0103	H-9->L+1 (50%)
38	36183.89	276.3660	0.0095	H-21->LUMO (44%)
39	36498.45	273.9842	0.0003	H-22->LUMO (56%)
40	36786.40	271.8396	0.0032	H-10->L+1 (13%), HOMO->L+4 (55%)
41	36833.98	271.4884	0.0088	H-23->LUMO (10%), H-21->LUMO (11%), H-11->L+1 (10%), H-9->L+1 (12%), HOMO->L+4 (17%)
42	36982.39	270.3990	0.0015	H-23->LUMO (20%), H-11->L+1 (28%), H-10->L+1 (38%)
43	37173.54	269.0085	0.0293	H-1->L+4 (34%), HOMO->L+6 (37%)
44	37232.42	268.5831	0.0041	H-1->L+4 (13%), HOMO->L+5 (39%), HOMO->L+6 (21%)
45	37272.75	268.2925	0.0329	H-1->L+4 (13%), HOMO->L+5 (42%), HOMO->L+6 (26%)
46	37300.17	268.0953	0.0051	H-13->L+1 (10%), H-12->L+1 (52%)
47	37424.38	267.2055	0.0063	H-13->L+1 (59%), H-11->L+1 (21%)
48	37551.82	266.2987	0.1852	H-2->L+2 (38%), H-2->L+3 (14%), H-1->L+4 (14%), HOMO->L+7 (15%)
49	37780.88	264.6841	0.0028	H-19->L+1 (19%), H-14->L+1 (18%), H-12->L+1 (20%)
50	37888.16	263.9347	0.0031	H-2->L+4 (70%)
51	37963.17	263.4132	0.0129	H-14->L+1 (10%), HOMO->L+7 (48%)
52	38118.03	262.3431	0.0652	H-1->L+5 (10%), H-1->L+7 (65%)
53	38301.11	261.0890	0.0038	H-16->L+1 (12%), H-14->L+1 (34%), H-1->L+7 (10%), HOMO->L+7 (11%)
54	38480.98	259.8687	0.0024	H-17->L+1 (36%), H-14->L+1 (14%)
55	38549.53	259.4065	0.0034	H-18->L+1 (13%), H-1->L+5 (30%), HOMO->L+9 (18%), HOMO->L+10 (12%)
56	38586.64	259.1571	0.0089	H-1->L+6 (12%), HOMO->L+9 (24%), HOMO->L+10 (13%)
57	38658.42	258.6759	0.0066	H-4->L+2 (16%), H-4->L+3 (14%)
58	38724.56	258.2341	0.0104	H-1->L+5 (26%), H-1->L+6 (31%)
59	38789.08	257.8045	0.0064	H-18->L+1 (30%), H-17->L+1 (11%), H-1->L+6 (24%)
60	38832.64	257.5153	0.0013	HOMO->L+8 (62%)

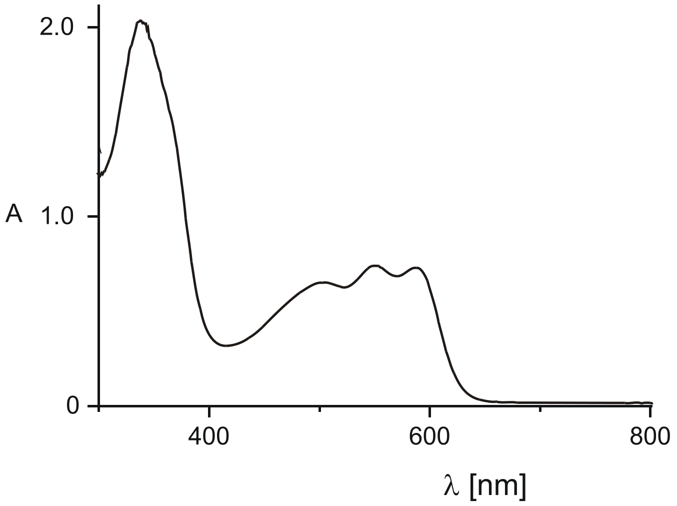

Figure S21. UV-Vis electronic spectrum of **1-PdCl₂** (CH_2Cl_2, 298 K).

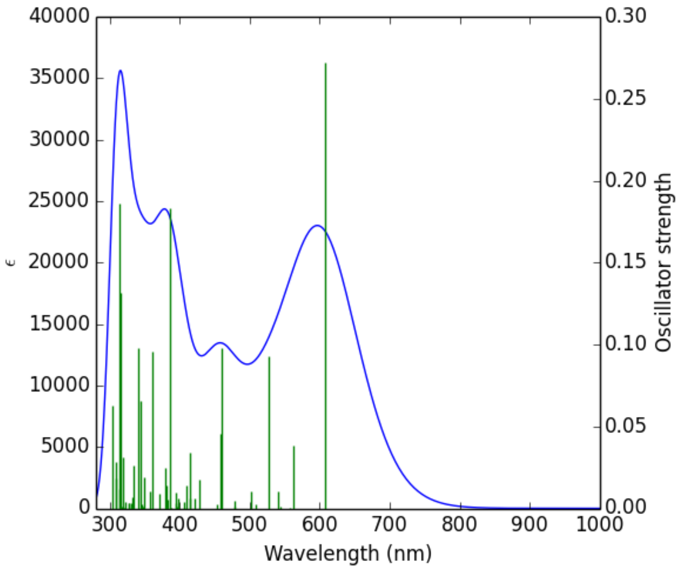

Figure S22. Simulated electronic spectrum (blue trace) and a histogram of electronic transitions (green sticks) for **1-PdCl₂** (num. pts.: 500; FWHM: 3000 cm^{-1}).

Table S5. UV-Vis transitions (oscillator strength > 0.01) calculated with TD-DFT for **1-PdCl₂**.

No.	Energy (cm^{-1})	Wavelength (nm)	Osc. Strength	Major contribs
1	16440.92	608.2385	0.2724	HOMO->LUMO (86%)
2	17783.84	562.3082	0.0384	H-2->LUMO (40%), H-2->L+1 (25%), H-1->LUMO (23%)
3	17912.89	558.2572	0.0007	H-4->LUMO (30%), H-4->L+1 (21%), H-3->LUMO (32%)
4	18378.28	544.1207	0.001	H-5->L+1 (11%), H-4->LUMO (22%), H-4->L+1 (13%), H-3->LUMO (21%)
5	18494.42	540.7036	0.0101	H-2->LUMO (19%), H-1->LUMO (58%)
6	18985.62	526.7145	0.0925	HOMO->L+1 (67%)
7	19678.45	508.1701	0.0024	H-7->LUMO (44%), H-7->L+1 (17%), HOMO->L+1 (23%)
8	19901.87	502.4654	0.0107	H-5->LUMO (34%), H-5->L+1 (13%), H-3->LUMO (32%)
9	20897.16	478.5339	0.0013	H-4->LUMO (30%), H-4->L+1 (34%), H-3->L+1 (12%)
10	20932.65	477.7226	0.0047	H-2->LUMO (31%), H-2->L+1 (41%), H-1->L+1 (11%)
11	21731.95	460.1519	0.0981	H-6->LUMO (32%), H-5->LUMO (25%), H-4->LUMO (10%)
12	21794.86	458.8237	0.0458	H-6->LUMO (49%), H-5->LUMO (20%)
13	22056.19	453.3875	0.0028	H-7->LUMO (37%), H-7->L+1 (27%), H-1->L+1 (21%)
14	22640.95	441.6777	0.0	H-14->LUMO (19%), H-8->LUMO (50%)
15	23324.10	428.7410	0.0177	H-12->LUMO (34%), H-10->LUMO (12%), H-1->L+1 (25%)

16	23520.10	425.1683	0.0	H-13->LUMO (11%), H-12->LUMO (25%), H-7->L+1 (19%), H-1->L+1 (32%)
17	23724.16	421.5113	0.0058	H-14->LUMO (10%), H-8->LUMO (10%), H-5->L+1 (38%), H-3->L+1 (24%)
18	24129.86	414.4244	0.034	H-15->LUMO (11%), H-13->LUMO (36%), H-10->LUMO (37%)
19	24150.02	414.0783	0.0061	H-21->LUMO (12%), H-16->LUMO (16%), H-14->LUMO (10%), H-9->LUMO (15%), H-8->LUMO (22%)
20	24393.60	409.9436	0.0138	H-14->LUMO (17%), H-9->LUMO (68%)
21	24558.14	407.1970	0.004	H-13->LUMO (26%), H-12->LUMO (36%), H-10->LUMO (33%)
22	24655.73	405.5852	0.0003	H-11->LUMO (81%)
23	25081.60	398.6987	0.0061	H-17->LUMO (11%), H-16->LUMO (37%), H-14->LUMO (33%)
24	25184.84	397.0643	0.0008	H-20->LUMO (14%), H-15->LUMO (55%), H-13->LUMO (13%)
25	25373.57	394.1109	0.0035	H-21->LUMO (15%), H-17->LUMO (71%)
26	25392.12	393.8229	0.0099	H-18->LUMO (81%)
27	25760.72	388.1879	0.0002	H-19->LUMO (73%)
28	25914.77	385.8803	0.1833	H-23->LUMO (16%), HOMO->L+2 (55%)
29	26160.77	382.2517	0.0052	H-6->L+1 (73%)
30	26255.14	380.8778	0.0141	H-20->LUMO (61%), H-19->LUMO (11%), H-15->LUMO (16%)
31	26358.38	379.3860	0.0248	H-21->LUMO (50%), H-17->LUMO (13%), H-16->LUMO (25%)
32	26896.36	371.7976	0.0091	H-24->LUMO (78%), H-19->LUMO (11%)
33	27656.14	361.5834	0.0954	H-14->L+1 (14%), H-8->L+1 (56%), H-3->L+1 (10%)
34	27980.37	357.3934	0.0105	H-22->LUMO (86%)
35	28651.43	349.0227	0.0187	H-13->L+1 (29%), H-12->L+1 (17%), H-10->L+1 (49%)
36	28803.87	347.1756	0.0012	H-14->L+1 (46%), H-9->L+1 (21%)
37	28877.27	346.2932	0.0023	H-13->L+1 (24%), H-12->L+1 (61%)
38	28905.50	345.9550	0.0008	H-11->L+1 (16%), H-9->L+1 (50%), H-8->L+1 (12%)
39	29069.23	344.0064	0.0653	H-25->LUMO (23%), H-23->LUMO (14%), H-1->L+2 (42%)
40	29090.20	343.7584	0.0001	H-13->L+1 (37%), H-12->L+1 (12%), H-10->L+1 (41%)
41	29279.74	341.5331	0.098	H-25->LUMO (23%), H-23->LUMO (20%), H-11->L+1 (20%), H-1->L+2 (24%)
42	29359.59	340.6042	0.0	H-11->L+1 (48%), H-9->L+1 (10%), H-1->L+2 (27%)
43	29970.96	333.6563	0.0263	H-15->L+1 (15%), H-3->L+2 (67%)
44	30114.53	332.0656	0.0069	H-21->L+1 (20%), H-16->L+1 (36%), H-14->L+1 (23%)
45	30182.28	331.3202	0.0031	H-20->L+1 (10%), H-15->L+1 (57%), H-3->L+2 (20%)
46	30297.62	330.0589	0.0014	H-2->L+2 (87%)
47	30570.24	327.1156	0.0031	H-19->L+1 (33%), H-18->L+1 (32%), H-4->L+2 (22%)
48	30602.50	326.7707	0.003	H-18->L+1 (12%), H-4->L+2 (68%)
49	31021.10	322.3612	0.004	H-17->L+1 (80%)
50	31100.15	321.5419	0.0001	H-20->L+1 (10%), H-19->L+1 (51%), H-18->L+1 (24%)
51	31290.50	319.5859	0.0309	H-21->L+1 (46%), H-16->L+1 (41%)
52	31386.48	318.6086	0.0008	H-20->L+1 (61%), H-18->L+1 (19%)
53	31563.92	316.8174	0.0014	H-26->LUMO (65%), H-26->L+1 (20%)
54	31693.78	315.5194	0.1315	H-25->LUMO (25%), H-23->LUMO (18%), H-7->L+2 (32%)
55	31881.70	313.6595	0.0119	H-5->L+2 (90%)
56	31909.93	313.3820	0.1857	H-23->LUMO (10%), H-7->L+2 (57%)
57	32278.53	309.8034	0.0285	H-6->L+2 (55%), HOMO->L+3 (23%)
58	32396.29	308.6773	0.018	H-6->L+2 (15%), HOMO->L+3 (69%)
59	32554.37	307.1784	0.0	H-27->LUMO (37%), H-27->L+1 (18%), H-24->L+1 (29%)
60	32815.70	304.7322	0.0626	H-22->L+1 (56%), HOMO->L+4 (26%)

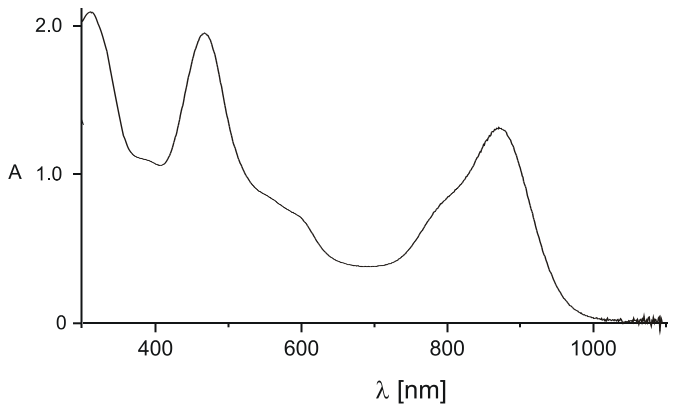

Figure S23. UV-Vis electronic spectrum of **2** (CH_2Cl_2, 298 K).

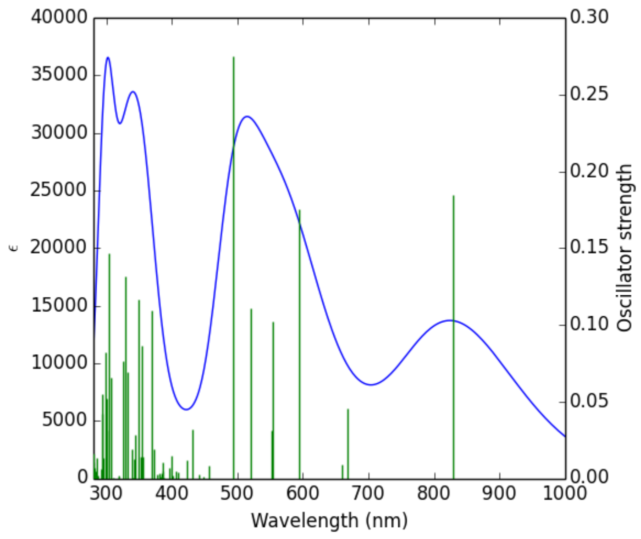

Figure S24. Simulated electronic spectrum (blue trace) and a histogram of electronic transitions (green sticks) for **2** (num. pts.: 500; FWHM: 3000 cm^{-1}).

Table S6. UV-Vis transitions (oscillator strength > 0.01) calculated with TD-DFT for **2**.

No.	Energy (cm^{-1})	Wavelength (nm)	Osc. Strength	Major contribs
1	12054.85	829.5419	0.1848	H-1->LUMO (13%), HOMO->LUMO (82%)
2	14951.20	668.8425	0.0454	H-3->LUMO (24%), H-2->LUMO (43%), H-1->LUMO (26%)
3	15146.39	660.2233	0.0091	H-3->LUMO (27%), H-2->LUMO (50%), H-1->LUMO (18%)
4	16836.13	593.9606	0.1756	H-5->LUMO (10%), H-3->LUMO (36%), H-1->LUMO (31%), HOMO->LUMO (12%)
5	18034.68	554.4872	0.1024	H-7->LUMO (16%), H-5->LUMO (26%), H-4->LUMO (29%), H-1->LUMO (11%)
6	18076.62	553.2007	0.0313	H-5->LUMO (41%), H-4->LUMO (51%)
7	19182.42	521.3108	0.1111	H-7->LUMO (68%), H-5->LUMO (15%)
8	20224.49	494.4500	0.275	H-6->LUMO (83%)
9	21898.91	456.6437	0.0079	HOMO->L+1 (11%), HOMO->L+2 (44%), HOMO->L+3 (12%)
10	22341.71	447.5933	0.0014	H-10->LUMO (15%), H-9->LUMO (50%)
11	22604.65	442.3868	0.0024	H-8->LUMO (90%)
12	23139.40	432.1633	0.0322	HOMO->L+1 (61%)
13	23619.30	423.3825	0.0115	H-19->LUMO (15%), H-14->LUMO (12%), H-10->LUMO (29%), H-9->LUMO (26%)
14	24395.21	409.9165	0.0038	H-19->LUMO (12%), H-14->LUMO (11%), H-11->LUMO (41%), H-10->LUMO (15%)
15	24628.31	406.0368	0.0047	H-11->LUMO (52%), H-10->LUMO (24%)

16	24881.57	401.9039	0.0016	H-3->L+2 (28%), H-1->L+2 (11%)
17	25044.49	399.2894	0.0148	H-13->LUMO (53%), H-12->LUMO (11%)
18	25195.32	396.8991	0.0068	H-13->LUMO (12%), H-12->LUMO (65%)
19	25766.37	388.1029	0.0005	H-15->LUMO (27%), H-14->LUMO (28%), H-1->L+1 (10%)
20	25859.12	386.7108	0.0106	H-14->LUMO (13%), H-3->L+1 (23%), H-1->L+1 (24%)
21	25887.35	386.2891	0.0044	H-20->LUMO (16%), H-17->LUMO (18%), H-16->LUMO (16%), H-15->LUMO (18%), H-2->L+1 (13%)
22	26032.53	384.1348	0.0035	H-20->LUMO (12%), H-17->LUMO (69%)
23	26095.44	383.2087	0.0014	H-19->LUMO (20%), H-14->LUMO (11%), H-3->L+1 (18%), H-2->L+1 (30%)
24	26275.31	380.5855	0.003	H-19->LUMO (10%), H-18->LUMO (30%), H-16->LUMO (11%), H-2->L+1 (28%)
25	26468.88	377.8022	0.0027	H-20->LUMO (16%), H-18->LUMO (31%), H-16->LUMO (11%), H-15->LUMO (20%)
26	26878.61	372.0430	0.0189	H-20->LUMO (20%), H-19->LUMO (15%), H-16->LUMO (41%)
27	27078.64	369.2948	0.1091	H-21->LUMO (27%), H-3->L+1 (15%), H-1->L+1 (21%)
28	28146.52	355.2837	0.0142	HOMO->L+2 (32%), HOMO->L+3 (28%)
29	28273.96	353.6823	0.0861	H-21->LUMO (31%), H-2->L+2 (14%)
30	28328.81	352.9976	0.0143	H-22->LUMO (90%)
31	28565.94	350.0673	0.1162	H-2->L+2 (41%)
32	29005.51	344.7621	0.0282	H-21->LUMO (12%), H-4->L+1 (49%)
33	29152.30	343.0261	0.0122	H-23->LUMO (71%), H-4->L+1 (20%)
34	29262.00	341.7402	0.0005	H-7->L+1 (15%), H-5->L+1 (47%), H-4->L+1 (13%)
35	29512.03	338.8449	0.0192	H-5->L+2 (13%), H-1->L+2 (30%), HOMO->L+3 (34%)
36	30123.40	331.9678	0.069	H-7->L+1 (64%), H-5->L+1 (12%)
37	30433.93	328.5806	0.1318	H-24->LUMO (47%), H-6->L+1 (19%)
38	30671.86	326.0317	0.0765	H-24->LUMO (15%), H-6->L+1 (69%)
39	31345.34	319.0267	0.0011	H-25->LUMO (28%), HOMO->L+4 (57%)
40	31395.35	318.5185	0.002	H-25->LUMO (59%), HOMO->L+4 (26%)
41	32159.16	310.9534	0.0002	H-3->L+3 (20%), H-1->L+2 (10%), H-1->L+3 (29%), HOMO->L+4 (10%)
42	32447.91	308.1863	0.0653	H-26->LUMO (33%), H-2->L+2 (15%)
43	32937.49	303.6054	0.1465	H-26->LUMO (27%), H-7->L+2 (11%), H-3->L+2 (12%)
44	33171.39	301.4646	0.0315	H-26->LUMO (13%), H-4->L+2 (10%), H-2->L+3 (41%)
45	33194.78	301.2522	0.052	H-4->L+2 (66%)
46	33378.68	299.5924	0.0824	HOMO->L+5 (55%)
47	33832.77	295.5714	0.0131	H-9->L+1 (11%), H-8->L+1 (76%)
48	33948.11	294.5672	0.0419	H-9->L+1 (19%), H-7->L+2 (21%)
49	34044.09	293.7367	0.055	H-9->L+1 (35%), H-8->L+1 (19%), H-2->L+4 (12%)
50	34074.74	293.4725	0.0019	H-2->L+4 (65%)
51	34177.98	292.5860	0.0062	H-5->L+2 (27%), H-3->L+3 (20%), H-1->L+3 (13%)
52	34749.02	287.7779	0.0018	H-19->L+1 (16%), H-14->L+1 (11%), H-10->L+1 (24%), H-9->L+1 (15%)
53	35085.36	285.0192	0.0131	H-6->L+2 (21%), H-4->L+4 (32%)
54	35227.31	283.8706	0.0047	H-6->L+2 (68%), H-4->L+4 (10%)
55	35395.08	282.5251	0.0068	H-3->L+4 (20%), H-1->L+4 (23%)
56	35479.77	281.8508	0.0035	H-11->L+1 (13%), H-10->L+1 (10%), H-3->L+4 (16%), H-1->L+4 (27%)
57	35649.95	280.5053	0.016	HOMO->L+6 (39%), HOMO->L+7 (18%)
58	35708.83	280.0428	0.0103	H-27->LUMO (47%), HOMO->L+7 (17%)
59	35804.00	279.2984	0.0008	H-11->L+1 (61%), H-10->L+1 (20%)
60	35918.54	278.4078	0.0032	H-27->LUMO (14%), HOMO->L+7 (42%)

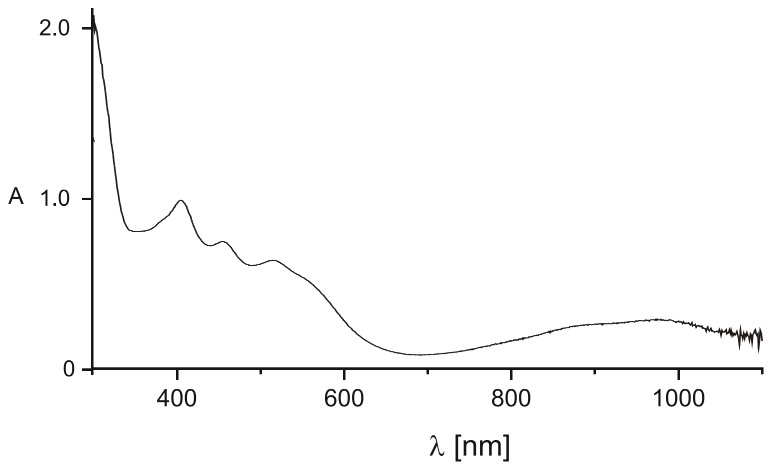

Figure S25. UV-Vis electronic spectrum of **3** (CH_2Cl_2, 298 K).

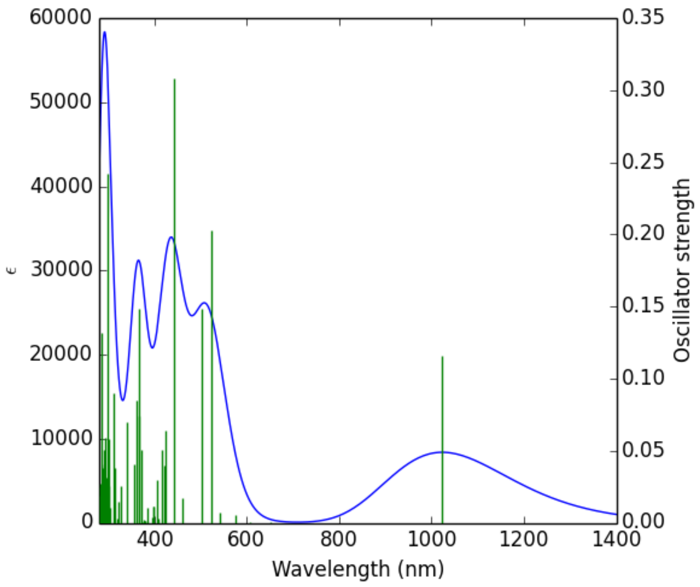

Figure S26. Simulated electronic spectrum (blue trace) and a histogram of electronic transitions (green sticks) for **3** (num. pts.: 500; FWHM: 3000 cm^{-1}).

Table S7. UV-Vis transitions (oscillator strength > 0.01) calculated with TD-DFT for **3**.

No.	Energy (cm^{-1})	Wavelength (nm)	Osc. Strength	Major contribs
1	9773.09	1023.2181	0.1157	HOMO->LUMO (97%)
2	15362.55	650.9337	0.0002	H-4->LUMO (14%), H-2->LUMO (15%), H-1->LUMO (69%)
3	16494.15	606.2755	0.0008	H-4->LUMO (83%), H-1->LUMO (12%)
4	17382.17	575.3020	0.0052	H-3->LUMO (10%), H-2->LUMO (76%), H-1->LUMO (13%)
5	18463.77	541.6012	0.0074	H-3->LUMO (38%), HOMO->L+1 (42%)
6	19086.44	523.9323	0.2031	H-5->LUMO (31%), H-3->LUMO (37%), HOMO->L+1 (18%)
7	19943.00	501.4290	0.1483	H-6->LUMO (36%), H-5->LUMO (25%), H-3->LUMO (11%), HOMO->L+1 (21%)
8	21728.73	460.2203	0.017	H-7->LUMO (85%)
9	22609.49	442.2922	0.3083	H-6->LUMO (37%), H-5->LUMO (27%)
10	23503.16	425.4747	0.0643	H-13->LUMO (11%), H-11->LUMO (10%), H-9->LUMO (22%), H-8->LUMO (17%)
11	23714.48	421.6833	0.0397	H-18->LUMO (16%), H-9->LUMO (42%)
12	23970.96	417.1714	0.0504	H-14->LUMO (13%), H-8->LUMO (52%)
13	24512.16	407.9607	0.0027	H-10->LUMO (57%), H-9->LUMO (14%)

14	24690.41	405.0155	0.0298	H-13->LUMO (14%), H-11->LUMO (21%), H-10->LUMO (25%), H-8->LUMO (14%)
15	25075.14	398.8013	0.011	H-4->L+1 (22%), H-2->L+1 (12%), H-1->L+1 (58%)
16	25161.45	397.4334	0.011	H-13->LUMO (26%), H-12->LUMO (66%)
17	25309.05	395.1156	0.0034	H-13->LUMO (20%), H-12->LUMO (10%), H-11->LUMO (50%)
18	25898.64	386.1206	0.0008	HOMO->L+2 (77%)
19	26031.72	384.1467	0.0101	H-18->LUMO (19%), H-15->LUMO (19%), H-14->LUMO (22%), HOMO->L+2 (20%)
20	26320.47	379.9324	0.0015	H-18->LUMO (20%), H-17->LUMO (13%), H-16->LUMO (27%), H-15->LUMO (13%), H-14->LUMO (12%)
21	26385.80	378.9917	0.0012	H-17->LUMO (15%), H-15->LUMO (59%)
22	26566.47	376.4143	0.0017	H-17->LUMO (16%), H-16->LUMO (60%)
23	26682.62	374.7758	0.0007	H-17->LUMO (21%), H-4->L+1 (47%), H-1->L+1 (13%)
24	26814.89	372.9271	0.0507	H-19->LUMO (24%), H-3->L+1 (15%), H-2->L+1 (16%), HOMO->L+3 (25%)
25	27235.11	367.1731	0.0741	H-2->L+1 (40%), HOMO->L+3 (39%)
26	27252.86	366.9340	0.1486	H-19->LUMO (53%), H-3->L+1 (10%), HOMO->L+3 (16%)
27	27611.78	362.1643	0.0849	H-3->L+1 (63%), H-2->L+1 (12%), HOMO->L+3 (12%)
28	27966.66	357.5686	0.0409	H-20->LUMO (75%)
29	29447.51	339.5873	0.0699	H-6->L+1 (12%), H-5->L+1 (77%)
30	30416.99	328.7636	0.0252	H-22->LUMO (29%), H-7->L+1 (29%), H-6->L+1 (25%)
31	30926.74	323.3448	0.0144	H-22->LUMO (47%), H-6->L+1 (34%), H-5->L+1 (11%)
32	31202.58	320.4863	0.0028	H-23->LUMO (87%)
33	31655.06	315.9053	0.0379	H-22->LUMO (16%), H-7->L+1 (44%), H-6->L+1 (17%)
34	32045.44	312.0569	0.0898	H-21->LUMO (58%), H-7->L+1 (12%)
35	32927.81	303.6946	0.0107	HOMO->L+5 (81%)
36	33207.69	301.1351	0.0258	H-1->L+2 (10%), HOMO->L+4 (52%)
37	33241.56	300.8282	0.0579	H-8->L+1 (16%), H-1->L+2 (17%), HOMO->L+4 (21%), HOMO->L+5 (10%)
38	33424.65	299.1804	0.0014	H-9->L+1 (22%), H-8->L+1 (44%)
39	33571.45	297.8722	0.2418	H-8->L+1 (19%), H-1->L+2 (36%)
40	33820.67	295.6771	0.031	H-4->L+2 (74%)
41	33923.91	294.7773	0.0058	HOMO->L+7 (92%)
42	34015.86	293.9805	0.0083	H-1->L+2 (11%), HOMO->L+6 (50%)
43	34083.61	293.3961	0.0588	H-14->L+1 (13%), H-10->L+1 (19%), H-9->L+1 (23%)
44	34206.21	292.3446	0.0505	H-3->L+2 (18%), H-2->L+2 (27%), H-1->L+3 (28%)
45	34365.10	290.9929	0.002	H-11->L+1 (46%), H-10->L+1 (10%), H-9->L+1 (26%)
46	34401.40	290.6859	0.0405	H-3->L+2 (15%), H-2->L+2 (18%), H-1->L+3 (35%)
47	34579.65	289.1875	0.0384	HOMO->L+8 (29%), HOMO->L+9 (36%)
48	34667.56	288.4541	0.0054	H-13->L+1 (11%), H-10->L+1 (58%)
49	34726.44	287.9650	0.012	HOMO->L+8 (44%), HOMO->L+9 (21%)
50	34804.68	287.3177	0.0148	H-12->L+1 (52%), H-11->L+1 (13%)
51	34851.46	286.9320	0.1317	H-3->L+2 (40%), H-2->L+2 (29%)
52	34983.73	285.8471	0.0044	H-16->L+1 (13%), H-15->L+1 (69%)
53	35303.94	283.2545	0.0273	H-24->LUMO (38%), H-4->L+3 (18%)
54	35323.30	283.0993	0.0187	HOMO->L+10 (62%)
55	35439.44	282.1715	0.0248	H-13->L+1 (49%), H-12->L+1 (11%)
56	35729.80	279.8784	0.0227	H-24->LUMO (10%), H-14->L+1 (12%), H-4->L+3 (22%), H-2->L+3 (11%)
57	35797.55	279.3487	0.101	H-18->L+1 (12%), H-14->L+1 (19%), H-2->L+3 (25%)
58	35944.35	278.2079	0.0139	H-24->LUMO (13%), H-4->L+3 (10%), H-2->L+3 (26%), HOMO->L+11 (18%)
59	36019.36	277.6285	0.0016	HOMO->L+11 (61%)
60	36254.87	275.8250	0.0124	H-19->L+1 (29%), H-17->L+1 (10%), HOMO->L+12 (25%)

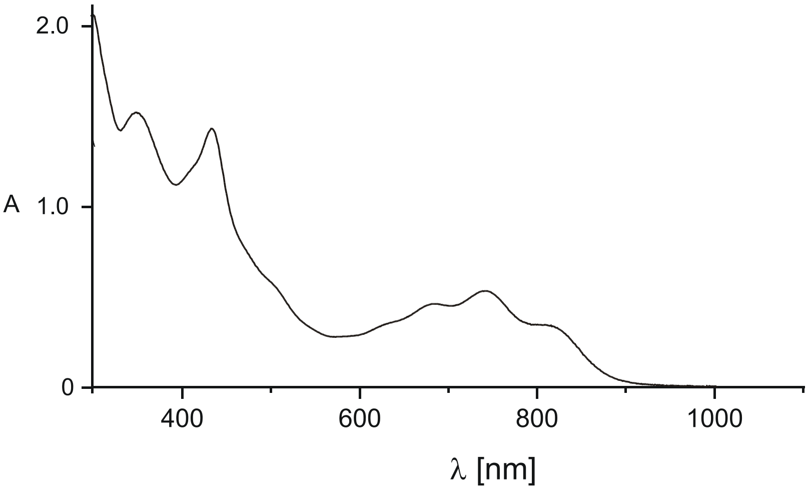

Figure S27. UV-Vis electronic spectrum of **3-Cl$_2$** (CH_2Cl_2, 298 K).

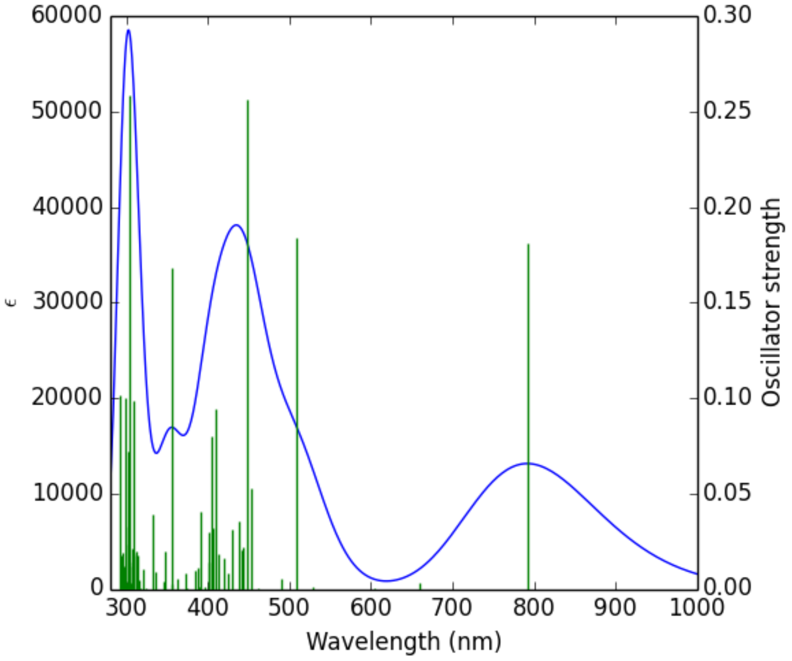

Figure S28. Simulated electronic spectrum (blue trace) and a histogram of electronic transitions (green sticks) for **3-Cl$_2$** (num. pts.: 500; FWHM: 3000 cm^{-1}).

Table S8. UV-Vis transitions (oscillator strength > 0.01) calculated with TD-DFT for **3-Cl$_2$**.

No.	Energy (cm^{-1})	Wavelength (nm)	Osc. Strength	Major contribs
1	12621.86	792.2764	0.1807	HOMO->LUMO (95%)
2	15152.84	659.9422	0.0034	H-2->LUMO (23%), H-1->LUMO (75%)
3	16536.09	604.7378	0.0004	H-2->LUMO (76%), H-1->LUMO (23%)
4	18899.31	529.1197	0.0009	HOMO->L+2 (92%)
5	19623.60	509.5904	0.1842	H-3->LUMO (74%), HOMO->L+1 (17%)
6	20386.61	490.5180	0.0056	H-4->LUMO (43%), H-3->LUMO (15%), HOMO->L+1 (36%)
7	21668.23	461.5051	0.0006	H-11->LUMO (18%), H-10->LUMO (18%), H-7->LUMO (22%), H-5->LUMO (28%)
8	22082.00	452.8575	0.0527	H-12->LUMO (10%), H-8->LUMO (24%), H-7->LUMO (19%)
9	22274.77	448.9385	0.2564	H-9->LUMO (10%), H-4->LUMO (35%), HOMO->L+1 (17%)
10	22544.16	443.5739	0.0217	H-6->LUMO (24%), H-5->LUMO (33%)
11	22668.37	441.1433	0.0205	H-12->LUMO (28%), H-10->LUMO (15%), H-5->LUMO (22%)

12	22802.26	438.5531	0.0352	H-7->LUMO (11%), H-6->LUMO (56%)
13	23269.26	429.7516	0.0311	H-13->LUMO (22%), H-7->LUMO (37%)
14	23579.78	424.0921	0.0081	H-9->LUMO (43%), H-8->LUMO (40%)
15	23819.33	419.8271	0.0159	H-17->LUMO (11%), H-15->LUMO (10%), H-11->LUMO (13%), H-10->LUMO (42%)
16	24183.09	413.5121	0.018	H-15->LUMO (24%), H-14->LUMO (15%), H-13->LUMO (10%), H-9->LUMO (13%)
17	24385.54	410.0792	0.094	H-2->L+1 (10%), H-1->L+1 (39%), H-1->L+2 (23%)
18	24581.53	406.8095	0.032	H-13->LUMO (15%), H-12->LUMO (15%), H-11->LUMO (18%), H-1->L+1 (12%), H-1->L+2 (11%)
19	24664.60	405.4393	0.0797	H-17->LUMO (11%), H-14->LUMO (23%), H-1->L+2 (17%)
20	24870.28	402.0864	0.0299	H-17->LUMO (12%), H-15->LUMO (27%), H-13->LUMO (27%)
21	24932.38	401.0848	0.0141	H-17->LUMO (22%), H-15->LUMO (13%), H-14->LUMO (22%)
22	25285.66	395.4811	0.0008	H-17->LUMO (27%), H-16->LUMO (52%)
23	25509.07	392.0174	0.0405	H-19->LUMO (77%)
24	25667.97	389.5907	0.0012	H-18->LUMO (67%), H-16->LUMO (14%)
25	25748.62	388.3703	0.0114	H-2->L+1 (41%), H-2->L+2 (31%), H-1->L+1 (13%), H-1->L+2 (10%)
26	26029.30	384.1824	0.0096	H-2->L+1 (30%), H-2->L+2 (41%), H-1->L+2 (16%)
27	26715.69	374.3119	0.0003	H-20->LUMO (26%), HOMO->L+3 (53%)
28	26814.89	372.9271	0.008	H-20->LUMO (55%), HOMO->L+3 (23%)
29	27578.71	362.5986	0.0052	H-13->L+2 (13%), H-3->L+2 (25%), HOMO->L+3 (18%)
30	28074.74	356.1921	0.0025	H-12->L+2 (45%), H-11->L+2 (14%)
31	28128.78	355.5078	0.168	H-3->L+1 (82%)
32	28719.99	348.1896	0.0196	H-21->LUMO (86%)
33	28856.30	346.5448	0.0038	H-13->L+2 (13%), H-3->L+2 (56%)
34	29793.52	335.6435	0.0088	H-4->L+1 (14%), H-4->L+2 (77%)
35	30141.15	331.7724	0.0394	H-4->L+1 (78%), H-4->L+2 (13%)
36	30876.73	323.8685	0.0003	H-13->L+1 (10%), H-10->L+1 (17%), H-7->L+1 (29%), H-5->L+1 (24%)
37	31247.75	320.0231	0.0103	H-22->LUMO (37%), H-2->L+3 (13%), H-1->L+3 (36%)
38	31457.45	317.8897	0.0001	H-12->L+1 (38%), H-11->L+1 (10%), H-7->L+1 (10%)
39	31736.52	315.0944	0.0048	H-6->L+1 (20%), H-5->L+1 (29%), H-5->L+2 (16%)
40	31880.90	313.6675	0.0179	H-6->L+1 (26%), H-5->L+2 (32%)
41	31993.82	312.5604	0.02	H-19->L+2 (17%), H-7->L+2 (16%), H-6->L+2 (15%), H-5->L+1 (10%), H-5->L+2 (11%)
42	32041.40	312.0962	0.0178	H-6->L+1 (20%), H-5->L+1 (27%)
43	32309.99	309.5018	0.0191	H-13->L+1 (16%), H-7->L+1 (39%)
44	32372.09	308.9081	0.0984	H-23->LUMO (38%), H-6->L+2 (15%)
45	32476.14	307.9184	0.0143	H-23->LUMO (27%), H-19->L+2 (13%), H-10->L+2 (11%)
46	32589.86	306.8439	0.0215	H-14->L+1 (12%), H-8->L+1 (30%)
47	32695.52	305.8523	0.0029	H-7->L+2 (15%), H-6->L+2 (42%)
48	32816.51	304.7247	0.0119	H-11->L+1 (12%), H-10->L+1 (30%), H-9->L+1 (12%)
49	32944.75	303.5385	0.2585	H-23->LUMO (11%), H-22->LUMO (11%), H-15->L+1 (14%)
50	33017.34	302.8712	0.0328	H-19->L+2 (10%), H-9->L+2 (19%), H-8->L+2 (18%)
51	33061.70	302.4648	0.0233	H-11->L+1 (10%), H-9->L+1 (23%), H-8->L+1 (20%)
52	33117.35	301.9565	0.0717	H-9->L+1 (18%), H-2->L+3 (48%), H-1->L+3 (19%)
53	33291.57	300.3763	0.0029	H-19->L+2 (17%), H-10->L+2 (17%), H-7->L+2 (23%)
54	33488.37	298.6111	0.1001	H-16->L+1 (13%), H-15->L+1 (10%), H-13->L+1 (15%)
55	33688.40	296.8381	0.0121	H-9->L+2 (27%), H-8->L+2 (41%)
56	33785.19	295.9877	0.019	H-17->L+1 (12%), H-16->L+1 (18%), H-15->L+1 (26%), H-13->L+1 (12%), H-12->L+1 (10%)
57	33861.00	295.3250	0.0123	H-15->L+2 (14%), H-11->L+2 (30%), H-9->L+2 (13%)
58	34041.67	293.7576	0.0177	H-17->L+1 (23%), H-14->L+1 (11%)
59	34072.32	293.4934	0.0087	H-17->L+1 (31%)
60	34131.20	292.9871	0.1014	H-24->LUMO (18%), HOMO->L+4 (20%)

Table S9. Cartesian coordinates for **1a**, **1b**, **1-PdCl$_2$**, **2**, **2-1**, **3**, **3-1**, **3-2**, and **3-Cl$_2$** (B3PW91/SDD for Rh, Pt and Te, and 6-31G(d,p) for the rest of atoms):

Cartesian coordinates for **1a**:

Center Number	Atomic Number	Coordinates (Angstroms) X	Y	Z
1	52	0.203983	1.590308	-0.106037
2	52	-0.347031	-2.459407	0.993689
3	6	3.548355	1.403359	-0.399879
4	6	-3.674620	-2.263892	0.767329
5	6	3.390624	-3.200891	-0.894839
6	7	-2.476603	0.916386	-0.249336
7	7	2.640849	0.361865	-0.269906
8	6	-3.125255	2.114905	0.027446
9	6	-3.541333	6.583322	1.633039
10	1	-3.291195	7.210545	2.484288
11	6	1.171042	-2.018893	-0.373094
12	6	-0.796801	-1.379196	-1.608436
13	1	-1.252060	-0.966112	-2.504826
14	6	-3.694451	-1.996454	-1.781609
15	6	-0.979765	3.321906	-0.024332
16	6	-3.343614	-3.266383	-2.268036
17	1	-2.632148	-3.868243	-1.710068
18	6	-4.763439	-2.976645	-4.201353
19	1	-5.174439	-3.352883	-5.134156
20	6	0.625079	-1.519621	-1.519632
21	1	1.256196	-1.166344	-2.331557
22	6	4.927163	3.923928	-1.600450
23	1	4.798293	3.214356	-2.412618
24	6	-3.151433	4.572102	0.329767
25	6	3.325208	-0.760401	-0.300170
26	6	-3.381397	-1.784400	2.055613
27	1	-2.782366	-0.884685	2.169011
28	6	4.885205	0.860637	-0.519904
29	1	5.799661	1.428733	-0.624741
30	6	4.749208	-0.495614	-0.469191
31	1	5.534563	-1.233288	-0.544864
32	6	2.810806	-2.479725	1.454287
33	6	-4.569786	6.956261	0.767635
34	6	2.854248	-3.729890	-2.071468
35	1	1.867407	-3.421615	-2.395908
36	6	-4.992565	-4.027074	1.794433
37	1	-5.632467	-4.897987	1.679735
38	6	-4.532690	1.858872	0.262694
39	1	-5.279991	2.594676	0.527394
40	6	2.660268	-2.123639	-0.047183
41	6	-4.888948	6.141244	-0.317246
42	1	-5.683022	6.429027	-1.000785
43	6	4.117176	3.813308	-0.461079
44	6	-2.413759	3.302891	0.102517
45	6	-0.204843	4.475414	-0.120865
46	1	-0.656711	5.463771	-0.140946
47	6	1.191861	4.327794	-0.227846
48	1	1.829466	5.199936	-0.346288
49	6	-4.576984	-1.224733	-2.542203
50	1	-4.854116	-0.229590	-2.210568
51	6	6.028427	5.860074	-0.664053
52	6	-4.188848	4.956405	-0.533023
53	1	-4.428629	4.326302	-1.384514
54	6	3.089419	-1.514521	2.427393
55	1	3.237881	-0.480881	2.134690
56	6	-3.077423	-1.511363	-0.449516
57	6	4.652400	-3.662485	-0.486065
58	1	5.078138	-3.302307	0.446028
59	6	1.720132	3.038595	-0.203397
60	6	-3.384542	-0.029092	-0.209024
61	6	5.359931	-4.595716	-1.238075
62	1	6.333963	-4.933620	-0.894059
63	6	-4.502492	-3.384767	0.656514
64	1	-4.774814	-3.762466	-0.322498
65	6	-5.104951	-1.709649	-3.741188
66	1	-5.783303	-1.084392	-4.315675
67	6	3.115282	2.720019	-0.352066
68	6	4.276705	4.746916	0.573888
69	1	3.660343	4.663320	1.464393
70	6	-1.563021	-1.718547	-0.532421
71	6	5.872652	4.942293	-1.701499
72	1	6.486647	5.019089	-2.594643
73	6	-2.832438	5.405595	1.413064
74	1	-2.036688	5.112145	2.091447
75	6	-3.876174	-3.754937	-3.456433
76	1	-3.588961	-4.742886	-3.806616
77	6	-4.698124	0.512519	0.126408
78	1	-5.606417	-0.062465	0.252112
79	6	-4.671936	-3.558753	3.065794
80	1	-5.055559	-4.060964	3.949575
81	6	-3.866427	-2.428213	3.191666
82	1	-3.621842	-2.035888	4.175000
83	6	2.601273	-3.804125	1.869949
84	1	2.398266	-4.571567	1.128644
85	6	3.559497	-4.669651	-2.825212

86	1	3.113511	-5.065343	-3.733930
87	6	4.817085	-5.103324	-2.417962
88	1	5.364648	-5.835681	-3.004809
89	6	3.162614	-1.862563	3.776139
90	1	3.383085	-1.095562	4.513641
91	6	5.229299	5.757656	0.474421
92	1	5.349157	6.465649	1.289910
93	6	2.667220	-4.149638	3.218232
94	1	2.502267	-5.181811	3.515295
95	6	2.951530	-3.179201	4.178100
96	1	3.010293	-3.448588	5.229073
97	1	6.767886	6.652171	-0.742241
98	1	-5.118094	7.878733	0.937350

Cartesian coordinates for **1b**:

Center Number	Atomic Number	Coordinates (Angstroms) X	Y	Z
1	52	-1.123357	0.161454	0.046876
2	52	2.953569	-0.410417	-0.961030
3	6	-1.888260	-3.108459	0.228816
4	6	3.699375	2.822615	-0.606831
5	6	2.562784	-4.264379	0.785744
6	7	0.274852	2.542034	0.321008
7	7	-0.632041	-2.524982	0.155827
8	8	-5.862063	7.217844	-0.978279
9	8	-8.109986	-4.809070	0.322322
10	6	-0.684984	3.507367	0.039776
11	6	-4.869700	5.183202	-1.558758
12	1	-5.565870	5.146884	-2.390740
13	6	2.063509	-1.786356	0.335675
14	6	1.968378	0.240259	1.636322
15	1	1.672670	0.764410	2.541391
16	6	3.367960	2.843872	1.933170
17	6	-2.448618	1.788643	0.008406
18	6	4.463575	2.127377	2.441627
19	1	4.850377	1.283167	1.878275
20	6	4.536337	3.529391	4.408077
21	1	4.984680	3.792915	5.362130
22	6	1.705423	-1.159709	1.493637
23	1	1.164511	-1.691677	2.272500
24	6	-4.701394	-3.812954	1.317818
25	1	-3.987249	-3.959104	2.122987
26	6	-3.030288	4.232125	-0.283313
27	6	0.253639	-3.497624	0.160995
28	6	3.196570	2.722865	-1.915546
29	1	2.166313	2.414015	-2.071383
30	6	-1.743535	-4.547296	0.282832
31	1	-2.546438	-5.270601	0.327395
32	6	-5.949436	8.354501	-0.142475
33	1	-6.756200	8.966400	-0.548558
34	1	-6.192852	8.079261	0.891865
35	1	-5.018283	8.935612	-0.150224
36	6	-0.401891	-4.795273	0.253341
37	1	0.084088	-5.759115	0.292764
38	6	2.089452	-3.425015	-1.548792
39	6	-4.918026	6.286226	-0.693834
40	6	3.186900	-3.938783	1.992643
41	1	3.155708	-2.917129	2.352447
42	6	5.799855	3.605004	-1.543609
43	1	6.814592	3.960548	-1.385243
44	6	-0.036409	4.786586	-0.165321
45	1	-0.523716	5.714371	-0.432921
46	6	1.754100	-3.232741	-0.047450
47	6	-4.023263	6.359872	0.378214
48	1	-4.046438	7.193727	1.070779
49	6	-4.361691	-2.976347	0.248858
50	6	-2.026141	3.164024	-0.070482
51	6	-3.773569	1.367376	0.089380
52	1	-4.594138	2.078499	0.138319
53	6	-4.026675	-0.017205	0.146649
54	1	-5.043407	-0.385320	0.255286
55	6	2.862966	3.898182	2.699015
56	1	2.004094	4.461108	2.348961
57	6	-6.875869	-4.246420	0.362770
58	6	-3.090471	5.341594	0.568643
59	1	-2.407875	5.397535	1.411508
60	6	1.107830	-3.381966	-2.544229
61	1	0.068074	-3.241010	-2.269937
62	6	2.769936	2.427285	0.569267
63	6	2.666807	-5.589663	0.332944
64	1	2.228649	-5.865083	-0.621968
65	6	-2.940363	-0.887891	0.091583
66	6	1.440173	3.145459	0.311028
67	6	3.345480	-6.555302	1.070563
68	1	3.408908	-7.572163	0.692022
69	6	5.007899	3.285135	-0.439909
70	1	5.417437	3.403621	0.556866
71	6	3.440455	4.236306	3.925140
72	1	3.021796	5.056199	4.502847
73	6	-3.031945	-2.321931	0.187992
74	6	-5.316310	-2.785169	-0.766602
75	1	-5.072194	-2.150631	-1.613554
76	6	2.541459	0.914185	0.598454
77	6	-5.944125	-4.441766	1.386639
78	1	-6.172722	-5.070896	2.239544

79	6	-3.947374	4.171975	-1.349393
80	1	-3.913637	3.327755	-2.031549
81	6	5.046273	2.468975	3.657650
82	1	5.895301	1.898206	4.024532
83	6	1.299676	4.561938	-0.005404
84	1	2.110432	5.271719	-0.107341
85	6	5.298914	3.475994	-2.836226
86	1	5.917951	3.726305	-3.693341
87	6	3.988386	3.037268	-3.017628
88	1	3.573166	2.948939	-4.017886
89	6	3.428161	-3.585487	-1.939590
90	1	4.204319	-3.635730	-1.181456
91	6	3.872308	-4.904016	2.732286
92	1	4.350809	-4.618224	3.665382
93	6	3.950871	-6.217499	2.280442
94	1	4.485771	-6.967797	2.856259
95	6	-8.487844	-5.661270	1.384764
96	1	-9.498071	-6.002232	1.153630
97	1	-7.823414	-6.531576	1.462208
98	1	-8.498679	-5.130843	2.345734
99	6	1.452039	-3.501869	-3.890549
100	1	0.671373	-3.467815	-4.645686
101	6	-6.549485	-3.413911	-0.717386
102	1	-7.281251	-3.280409	-1.507881
103	6	3.772065	-3.698097	-3.285267
104	1	4.815284	-3.822709	-3.562851
105	6	2.783493	-3.659605	-4.267640
106	1	3.049533	-3.754220	-5.316870

Cartesian coordinates for **1-PdCl$_2$**:

Center Number	Atomic Number	Coordinates (Angstroms) X	Y	Z
1	52	0.048236	1.325549	-0.899761
2	52	-0.070324	-1.983621	0.997396
3	46	0.016831	0.596273	1.621222
4	17	0.095445	2.818475	2.284993
5	17	-0.019108	-0.124625	3.841019
6	7	-2.582711	0.502420	-0.787493
7	7	2.614409	0.323597	-0.789562
8	6	1.478649	2.903886	-0.937854
9	6	0.857613	4.114793	-1.121464
10	1	1.430668	5.030994	-1.237103
11	6	-0.568088	4.163956	-1.119924
12	1	-1.076927	5.117559	-1.233562
13	6	-1.270497	2.998488	-0.935716
14	6	-2.696231	2.909050	-0.792047
15	6	-3.319956	1.680899	-0.696985
16	6	-4.701460	1.368287	-0.391897
17	1	-5.496188	2.085803	-0.240915
18	6	-4.765252	0.012508	-0.287840
19	1	-5.621940	-0.598482	-0.035883
20	6	-3.416216	-0.480387	-0.546335
21	6	-2.985968	-1.940625	-0.458235
22	6	-1.464564	-2.025925	-0.604306
23	6	-0.793756	-2.120828	-1.776204
24	1	-1.330734	-2.173400	-2.720044
25	6	0.650946	-2.170365	-1.773787
26	1	1.186284	-2.259937	-2.715793
27	6	1.322920	-2.120639	-0.599739
28	6	2.846291	-2.140293	-0.450246
29	6	3.377842	-0.714499	-0.547775
30	6	4.758870	-0.316615	-0.294992
31	1	5.571457	-0.985383	-0.043676
32	6	4.789783	1.040036	-0.402680
33	1	5.633257	1.700532	-0.256022
34	6	3.432537	1.447806	-0.704132
35	6	2.895323	2.716258	-0.798920
36	6	-3.498286	4.152731	-0.672698
37	6	-4.538216	4.421899	-1.573473
38	1	-4.735951	3.723814	-2.382133
39	6	-5.297585	5.582997	-1.445927
40	1	-6.094461	5.786108	-2.156004
41	6	-5.034468	6.480906	-0.412481
42	1	-5.630057	7.383879	-0.311020
43	6	-4.005759	6.216191	0.491973
44	1	-3.804253	6.906719	1.306063
45	6	-3.235144	5.063849	0.362266
46	1	-2.444496	4.843874	1.074753
47	6	-3.614211	-2.777905	-1.593499
48	6	-3.250962	-4.127883	-1.730948
49	1	-2.532899	-4.559005	-1.039393
50	6	-3.784018	-4.917596	-2.744055
51	1	-3.488198	-5.960193	-2.823269
52	6	-4.685942	-4.372208	-3.659011
53	1	-5.099798	-4.986016	-4.454120
54	6	-5.039378	-3.032116	-3.547238
55	1	-5.729742	-2.587810	-4.259062
56	6	-4.507789	-2.242291	-2.524923
57	1	-4.793955	-1.197355	-2.466274
58	6	-3.445456	-2.415352	0.947819
59	6	-3.101144	-1.627305	2.058747
60	1	-2.579123	-0.681640	1.925485
61	6	-3.439971	-2.015039	3.352974
62	1	-3.129232	-1.392154	4.185970
63	6	-4.165931	-3.186029	3.561631

64	1	-4.436803	-3.491614	4.568306
65	6	-4.553075	-3.952417	2.465252
66	1	-5.136355	-4.857769	2.611344
67	6	-4.198297	-3.572216	1.169772
68	1	-4.519944	-4.184236	0.335139
69	6	3.415234	-3.028500	-1.577680
70	6	4.338079	-2.562121	-2.517682
71	1	4.692625	-1.537741	-2.470740
72	6	4.811909	-3.395362	-3.533990
73	1	5.527054	-3.004671	-4.252763
74	6	4.370125	-4.710134	-3.631085
75	1	4.739090	-5.357672	-4.421521
76	6	3.437928	-5.185710	-2.707510
77	1	3.073346	-6.207115	-2.775349
78	6	2.962706	-4.352655	-1.700416
79	1	2.220494	-4.728410	-1.002050
80	6	3.270010	-2.633606	0.960660
81	6	3.947785	-3.833966	1.193372
82	1	4.232095	-4.471372	0.364199
83	6	4.274479	-4.225989	2.492740
84	1	4.798939	-5.165283	2.647289
85	6	3.933771	-3.428377	3.582257
86	1	4.182093	-3.742921	4.591982
87	6	3.284104	-2.215303	3.362749
88	1	3.011330	-1.567725	4.190192
89	6	2.973909	-1.816763	2.064667
90	1	2.513543	-0.840787	1.922625
91	6	3.782137	3.901453	-0.684862
92	6	3.584807	4.832525	0.346996
93	1	2.781959	4.670897	1.061417
94	6	4.433783	5.929036	0.471324
95	1	4.282033	6.634867	1.283079
96	6	5.476937	6.118301	-0.435505
97	1	6.133998	6.978065	-0.338291
98	6	5.675310	5.200433	-1.465868
99	1	6.483197	5.345048	-2.177822
100	6	4.836855	4.094494	-1.588039
101	1	4.984173	3.381558	-2.394485

Cartesian coordinates for **2**:

Center Number	Atomic Number	Coordinates (Angstroms) X	Y	Z
1	6	-9.783137	8.532788	18.303057
2	17	-12.249581	9.719153	19.771272
3	6	-8.667792	8.757412	19.126028
4	1	-7.954331	7.967399	19.356790
5	17	-9.454870	10.671313	16.058201
6	6	-8.476995	10.065533	19.602345
7	1	-7.635159	10.346995	20.230180
8	6	-9.472863	10.942798	19.215602
9	6	-9.641032	12.302832	19.651187
10	6	-10.706377	12.976208	19.089698
11	6	-11.247381	14.280806	19.353176
12	1	-10.873471	14.983538	20.084411
13	6	-12.309162	14.422481	18.514163
14	1	-12.972564	15.270923	18.416261
15	6	-12.414552	13.193448	17.740374
16	6	-13.473121	13.015431	16.651376
17	6	-13.135108	11.882808	15.689621
18	6	-12.833770	11.966047	14.369056
19	1	-12.793205	12.924389	13.861182
20	6	-12.533015	10.763638	13.636311
21	1	-12.244822	10.832427	12.591086
22	6	-12.588689	9.567630	14.271315
23	6	-12.315484	8.185183	13.659166
24	6	-11.780069	7.296395	14.770968
25	6	-12.145279	6.001639	15.175411
26	1	-12.861232	5.369258	14.673379
27	6	-11.476829	5.729209	16.367702
28	1	-11.583195	4.850722	16.987742
29	6	-10.715731	6.868383	16.702318
30	6	-10.020083	7.190315	17.896111
31	45	-10.804200	10.269733	17.975351
32	7	-11.479800	12.337057	18.097388
33	52	-13.139295	9.870555	16.295938
34	7	-10.904118	7.777990	15.686032
35	6	-8.756271	12.936495	20.646833
36	6	-8.498939	12.324423	21.880332
37	1	-8.977392	11.377674	22.112663
38	6	-7.676875	12.925091	22.831123
39	1	-7.517263	12.428888	23.781755
40	8	-6.258841	14.828616	23.398655
41	6	-7.079713	14.159226	22.551864
42	6	-7.321695	14.780767	21.318549
43	1	-6.840399	15.731921	21.114249
44	6	-8.150490	14.179740	20.385984
45	1	-8.316517	14.659773	19.425942
46	6	-5.973705	14.243112	24.653560
47	1	-5.301466	14.934826	25.163234
48	1	-6.881871	14.114539	25.256377
49	1	-5.474580	13.271848	24.543151
50	6	-13.462507	14.367421	15.889552
51	6	-12.243100	14.817022	15.354368
52	1	-11.352982	14.203402	15.466844
53	6	-12.158851	16.033837	14.685428

54	1	-11.204743	16.355358	14.276747
55	6	-13.290020	16.839309	14.548260
56	1	-13.225945	17.791756	14.029333
57	6	-14.497725	16.414230	15.092330
58	1	-15.384652	17.036255	15.005534
59	6	-14.583810	15.190040	15.759749
60	1	-15.532848	14.886475	16.186967
61	6	-14.849931	12.700214	17.276519
62	6	-15.941802	12.458509	16.427906
63	1	-15.799685	12.509825	15.351686
64	6	-17.194505	12.146807	16.945429
65	1	-18.026663	11.969120	16.269361
66	6	-17.378438	12.051322	18.326208
67	1	-18.353929	11.798595	18.732407
68	6	-16.298497	12.268245	19.175149
69	1	-16.420862	12.177255	20.250680
70	6	-15.043037	12.591921	18.655600
71	1	-14.210742	12.732568	19.337089
72	6	-13.602232	7.523666	13.111907
73	6	-14.875694	8.043131	13.348977
74	1	-14.985991	8.959297	13.919120
75	6	-16.012631	7.401388	12.853063
76	1	-16.993172	7.826198	13.050215
77	6	-15.893767	6.230856	12.110550
78	1	-16.778699	5.733043	11.723847
79	6	-14.624977	5.706991	11.859569
80	1	-14.514806	4.799187	11.272351
81	6	-13.492892	6.349288	12.351779
82	1	-12.508591	5.943219	12.135185
83	6	-11.299624	8.345111	12.499546
84	6	-9.927965	8.136314	12.669314
85	1	-9.535101	7.818071	13.628218
86	6	-9.039387	8.331030	11.611900
87	1	-7.978203	8.161158	11.771328
88	6	-9.503637	8.740347	10.365195
89	1	-8.809825	8.892072	9.543062
90	6	-10.869706	8.948783	10.182205
91	1	-11.250645	9.262189	9.213854
92	6	-11.757212	8.748093	11.236325
93	1	-12.820967	8.893985	11.073805
94	6	-9.609486	6.067948	18.750913
95	6	-9.044785	4.900009	18.210364
96	1	-8.882833	4.837300	17.138431
97	6	-8.645824	3.843416	19.021010
98	1	-8.186779	2.970959	18.569984
99	8	-8.488500	2.957435	21.286959
100	6	-8.830398	3.927141	20.408058
101	6	-9.408630	5.078587	20.963997
102	1	-9.562392	5.113706	22.037751
103	6	-9.784131	6.132123	20.150649
104	1	-10.261767	7.008717	20.578125
105	6	-7.921342	1.762339	20.784243
106	1	-7.739297	1.127836	21.652543
107	1	-8.606360	1.247801	20.098810
108	1	-6.970190	1.951451	20.270880
109	1	-10.445962	8.699953	15.664343

Cartesian coordinates for **2-1**:

Center Number	Atomic Number	Coordinates (Angstroms) X	Y	Z
1	6	-9.745862	8.050239	18.656578
2	6	-8.704738	8.815220	19.161321
3	1	-7.676070	8.469328	19.094082
4	6	-9.007915	10.099004	19.655684
5	1	-8.222447	10.768293	19.994908
6	6	-10.323073	10.542352	19.600651
7	6	-10.755475	11.875334	19.938630
8	6	-11.760379	12.549748	19.267731
9	6	-12.288852	13.844934	19.650818
10	1	-12.065204	14.358532	20.574800
11	6	-13.121561	14.243653	18.655835
12	1	-13.702857	15.152663	18.596749
13	6	-13.100108	13.191031	17.662131
14	6	-13.722334	13.245875	16.285115
15	6	-13.607955	11.853738	15.660706
16	6	-14.186159	11.435810	14.512847
17	1	-14.779281	12.092547	13.878129
18	6	-13.969301	10.055965	14.174961
19	1	-14.411006	9.646445	13.266484
20	6	-13.232460	9.299736	15.034813
21	6	-13.035428	7.816185	14.610467
22	6	-12.272999	6.973694	15.599097
23	6	-12.378084	5.592474	15.903898
24	1	-13.164563	4.942815	15.548453
25	6	-11.343559	5.265552	16.764024
26	1	-11.168680	4.314544	17.247850
27	6	-10.626039	6.457442	17.044186
28	6	-9.694998	6.753993	18.059032
29	7	-12.345326	12.173860	18.038406
30	7	-11.231213	7.459803	16.288288
31	6	-10.002892	12.558007	21.016547
32	6	-9.773978	11.916703	22.241928
33	1	-10.171532	10.918231	22.400484
34	6	-9.075476	12.539511	23.271791
35	1	-8.940574	12.018505	24.212849

36	8	-7.861267	14.524104	24.005544
37	6	-8.561715	13.828063	23.079258
38	6	-8.756670	14.473070	21.849478
39	1	-8.330504	15.461273	21.709119
40	6	-9.467692	13.848299	20.838697
41	1	-9.594324	14.347609	19.882973
42	6	-7.629089	13.920713	25.263874
43	1	-7.057538	14.646052	25.844327
44	1	-8.568751	13.701590	25.786527
45	1	-7.045262	12.996474	25.167467
46	6	-12.861783	14.293239	15.533455
47	6	-11.766850	13.890014	14.761731
48	1	-11.546203	12.832883	14.657600
49	6	-10.948418	14.833814	14.142443
50	1	-10.104306	14.496060	13.547110
51	6	-11.203137	16.195478	14.288229
52	1	-10.565679	16.929330	13.802059
53	6	-12.283868	16.608980	15.066372
54	1	-12.496119	17.667939	15.189497
55	6	-13.101604	15.667487	15.686108
56	1	-13.949333	16.005839	16.274324
57	6	-15.207337	13.636605	16.348620
58	6	-15.828615	14.303180	15.284222
59	1	-15.240901	14.609851	14.424168
60	6	-17.192071	14.588057	15.318362
61	1	-17.652048	15.105858	14.480615
62	6	-17.961061	14.215810	16.419663
63	1	-19.023670	14.441587	16.448220
64	6	-17.354831	13.544282	17.479217
65	1	-17.943934	13.233456	18.337877
66	6	-15.993075	13.251717	17.441557
67	1	-15.544114	12.685876	18.251967
68	6	-14.439969	7.205135	14.422223
69	6	-15.243613	7.021425	15.557949
70	1	-14.868079	7.320425	16.532783
71	6	-16.534725	6.518424	15.445031
72	1	-17.138391	6.391274	16.339591
73	6	-17.060214	6.199675	14.191431
74	1	-18.070779	5.810152	14.102465
75	6	-16.281578	6.402267	13.056909
76	1	-16.681018	6.175264	12.071766
77	6	-14.981411	6.900415	13.170435
78	1	-14.392969	7.055131	12.272260
79	6	-12.146206	7.689478	13.328252
80	6	-11.970333	6.429835	12.730917
81	1	-12.487061	5.564805	13.135100
82	6	-11.155600	6.268464	11.614191
83	1	-11.051012	5.284193	11.164868
84	6	-10.475709	7.362158	11.079150
85	1	-9.836501	7.239663	10.208849
86	6	-10.623723	8.610161	11.676541
87	1	-10.094110	9.472197	11.279764
88	6	-11.450588	8.775328	12.788926
89	1	-11.546758	9.751456	13.247562
90	6	-8.753155	5.736887	18.514048
91	6	-8.262641	4.762606	17.625045
92	1	-8.565211	4.799575	16.583070
93	6	-7.367632	3.784688	18.039212
94	1	-6.997501	3.064302	17.318829
95	8	-6.074372	2.856968	19.884078
96	6	-6.938688	3.757723	19.373447
97	6	-7.416286	4.723894	20.275909
98	1	-7.081093	4.677132	21.306961
99	6	-8.301242	5.694716	19.851838
100	1	-8.682170	6.421544	20.562299
101	6	-5.566264	1.846146	19.030080
102	1	-4.908563	1.234953	19.648763
103	1	-6.370919	1.218827	18.627849
104	1	-4.988541	2.273718	18.201655
105	52	-11.586341	9.061619	18.889066
106	17	-14.643548	9.951118	17.792157
107	45	-12.629074	10.459400	16.638672
108	17	-10.447175	10.610133	15.701224
109	1	-10.840644	8.397315	16.121804

Cartesian coordinates for **3**:

Center Number	Atomic Number	Coordinates (Angstroms) X	Y	Z
1	78	8.464606	3.366199	3.891917
2	52	5.956793	3.103827	4.750075
3	7	7.858984	4.279484	2.130210
4	7	7.188424	2.768002	7.025992
5	6	9.568643	2.520233	5.318532
6	6	10.933552	2.404548	4.966030
7	6	11.311854	2.915224	3.705286
8	1	12.340040	2.924934	3.348545
9	6	10.240088	3.431670	2.987943
10	6	10.228598	4.039977	1.704906
11	6	8.970344	4.479078	1.274451
12	6	8.514275	5.061264	0.063548
13	1	9.127539	5.314330	-0.789854
14	6	7.152668	5.214281	0.196963
15	1	6.466037	5.619755	-0.533285
16	6	6.774349	4.717095	1.489298
17	6	5.317977	4.596003	1.959990

18	6	5.170383	4.626807	3.484339
19	6	4.561446	5.572898	4.240093
20	1	4.096150	6.450314	3.799698
21	6	4.533456	5.414248	5.683398
22	1	4.011627	6.149530	6.292944
23	6	5.160246	4.357872	6.248868
24	6	5.143492	3.916604	7.708726
25	6	6.479550	3.245815	8.011574
26	6	7.171075	3.088753	9.281377
27	1	6.817615	3.427563	10.246164
28	6	8.359272	2.482183	8.989196
29	1	9.166845	2.275382	9.677094
30	6	8.398497	2.326535	7.543188
31	6	9.476136	2.090175	6.710882
32	6	11.435191	4.207212	0.868024
33	6	12.269705	3.113498	0.587708
34	1	12.002292	2.132035	0.968152
35	6	13.409096	3.274510	-0.196255
36	1	14.037941	2.415427	-0.413615
37	6	13.738828	4.529404	-0.707147
38	1	14.629758	4.653936	-1.316318
39	6	12.918905	5.623634	-0.432935
40	1	13.172946	6.605948	-0.821826
41	6	11.774894	5.464918	0.344428
42	1	11.145488	6.320446	0.571794
43	6	4.559035	5.799119	1.348374
44	6	5.056688	7.093451	1.572572
45	1	5.962984	7.219841	2.158530
46	6	4.413384	8.212141	1.052690
47	1	4.819669	9.201978	1.243046
48	6	3.256970	8.063120	0.285842
49	1	2.754603	8.934610	-0.124915
50	6	2.759883	6.785990	0.047580
51	1	1.865834	6.652065	-0.555631
52	6	3.404888	5.663996	0.573593
53	1	3.003649	4.678016	0.367344
54	6	4.727044	3.250086	1.463918
55	6	5.346678	2.468568	0.484145
56	1	6.285466	2.791218	0.046182
57	6	4.775968	1.266937	0.060869
58	1	5.282922	0.673836	-0.695481
59	6	3.571895	0.827655	0.602930
60	1	3.130393	-0.109102	0.274231
61	6	2.939712	1.601178	1.576513
62	1	1.999433	1.272675	2.011152
63	6	3.514503	2.794835	2.003951
64	1	3.020299	3.386591	2.769438
65	6	5.025629	5.143117	8.637293
66	6	5.962270	6.179713	8.504092
67	1	6.720413	6.115763	7.728343
68	6	5.926743	7.285775	9.346627
69	1	6.660825	8.077598	9.224393
70	6	4.954832	7.377549	10.344250
71	1	4.924845	8.241890	11.001968
72	6	4.026638	6.351280	10.489767
73	1	3.266543	6.408212	11.264404
74	6	4.062716	5.239934	9.644376
75	1	3.333745	4.446981	9.773299
76	6	3.980770	2.915298	7.917626
77	6	1.633712	2.346524	7.638322
78	1	0.649017	2.607695	7.259869
79	6	2.698564	3.221370	7.442286
80	1	2.535610	4.151625	6.907344
81	6	1.829453	1.138972	8.308664
82	1	1.000784	0.451727	8.454853
83	6	3.098542	0.822609	8.783860
84	1	3.268904	-0.115228	9.305609
85	6	4.163583	1.704153	8.592668
86	1	5.144914	1.436629	8.972075
87	6	10.802857	1.576469	7.126385
88	6	11.227333	0.923159	8.275241
89	1	10.536388	0.691373	9.079483
90	6	12.570192	0.529270	8.376706
91	1	12.913389	0.021584	9.273510
92	6	13.460217	0.774563	7.335475
93	1	14.497039	0.463039	7.428772
94	6	13.029752	1.403879	6.157356
95	1	13.726247	1.571073	5.340277
96	6	11.703025	1.792051	6.055828

Cartesian coordinates for **3-1**:

Center Number	Atomic Number	Coordinates (Angstroms) X	Y	Z
1	7	7.766297	4.049921	1.780125
2	7	7.000841	2.719266	6.691553
3	6	9.361805	1.813134	5.063055
4	6	10.721581	1.952966	4.801337
5	6	11.094549	2.611875	3.603187
6	1	12.133233	2.829881	3.367674
7	6	10.064565	3.085230	2.800070
8	6	10.132687	3.992796	1.694966
9	6	8.932775	4.522649	1.226696
10	6	8.598610	5.602319	0.325219
11	1	9.287954	6.153424	-0.299786
12	6	7.247457	5.789862	0.439090

13	1	6.646735	6.524337	-0.077090
14	6	6.752864	4.792208	1.381869
15	6	5.338555	4.502272	1.879193
16	6	5.272203	4.640652	3.439193
17	6	4.264607	5.357994	4.019506
18	1	3.551754	5.957301	3.458343
19	6	4.099990	5.227976	5.460575
20	1	3.274663	5.723330	5.972034
21	6	4.993953	4.417594	6.071529
22	6	5.093351	4.053035	7.555793
23	6	6.234617	3.068739	7.719927
24	6	6.727036	2.490609	8.948569
25	1	6.280995	2.612495	9.925042
26	6	7.862106	1.813558	8.627699
27	1	8.506471	1.280178	9.309314
28	6	8.062945	1.943976	7.200281
29	6	9.169484	1.553780	6.458443
30	6	11.440387	4.449015	1.172435
31	6	12.383552	3.514374	0.717200
32	1	12.139092	2.456155	0.744516
33	6	13.607734	3.936838	0.206646
34	1	14.321815	3.202522	-0.155636
35	6	13.915016	5.296421	0.155634
36	1	14.872682	5.624557	-0.238497
37	6	12.990146	6.232130	0.616762
38	1	13.227900	7.291920	0.591171
39	6	11.760989	5.813870	1.120612
40	1	11.049924	6.540945	1.501317
41	6	4.366051	5.483598	1.191396
42	6	4.545966	6.867775	1.355221
43	1	5.356849	7.226585	1.982638
44	6	3.692057	7.785301	0.750579
45	1	3.857427	8.849647	0.896869
46	6	2.624105	7.341825	-0.030376
47	1	1.954520	8.055795	-0.502306
48	6	2.424263	5.974639	-0.190279
49	1	1.593600	5.610887	-0.789468
50	6	3.285656	5.056427	0.414350
51	1	3.108987	3.995653	0.275361
52	6	4.990638	3.044968	1.498360
53	6	5.431868	2.495881	0.286900
54	1	6.055659	3.088527	-0.377265
55	6	5.076949	1.201513	-0.086447
56	1	5.430648	0.798661	-1.032026
57	6	4.270055	0.427527	0.747740
58	1	3.995388	-0.583888	0.460223
59	6	3.818629	0.965766	1.950467
60	1	3.186626	0.376037	2.609446
61	6	4.174270	2.263114	2.322285
62	1	3.824537	2.674069	3.263699
63	6	5.499960	5.255047	8.442114
64	6	6.278002	6.285706	7.899661
65	1	6.524872	6.260598	6.842607
66	6	6.723714	7.336338	8.698208
67	1	7.321576	8.128284	8.254945
68	6	6.403065	7.377394	10.054349
69	1	6.745691	8.200973	10.674866
70	6	5.636922	6.353053	10.606486
71	1	5.378126	6.372093	11.661953
72	6	5.195037	5.298354	9.809194
73	1	4.590978	4.512197	10.252691
74	6	3.752169	3.419754	7.961336
75	6	1.433164	3.651817	8.647175
76	1	0.612946	4.291308	8.962660
77	6	2.675333	4.217806	8.368783
78	1	2.810023	5.290086	8.473264
79	6	1.240717	2.276816	8.523544
80	1	0.272648	1.835915	8.744452
81	6	2.303116	1.474581	8.112437
82	1	2.168515	0.401237	8.007771
83	6	3.545146	2.040945	7.832385
84	1	4.359196	1.402909	7.499873
85	6	10.514703	1.169008	6.987520
86	6	10.944387	0.622500	8.189171
87	1	10.244424	0.321076	8.960198
88	6	12.315706	0.416550	8.397410
89	1	12.651894	-0.023902	9.331499
90	6	13.244409	0.765190	7.421923
91	1	14.303645	0.605951	7.603129
92	6	12.823439	1.306701	6.199898
93	1	13.550492	1.566329	5.435307
94	6	11.466835	1.487736	5.981059
95	78	6.336836	3.605821	4.868938
96	52	8.166648	2.377423	3.438257

Cartesian coordinates for **3-2**:

Center Number	Atomic Number	Coordinates (Angstroms) X	Y	Z
1	78	8.410073	3.657084	3.700823
2	52	5.936089	3.151256	4.567288
3	7	7.651131	4.535008	1.894272
4	7	7.369659	2.826815	6.718748
5	6	9.812754	2.751538	4.920546
6	6	11.058227	2.566570	4.255971
7	6	11.152240	3.157915	2.971642

8	6	9.975677	3.785320	2.640288
9	6	9.950844	4.270069	1.315146
10	6	8.691837	4.685653	0.910194
11	6	8.105929	5.166296	-0.293774
12	1	8.633243	5.386674	-1.211818
13	6	6.763030	5.292510	-0.052409
14	1	6.000251	5.632512	-0.739699
15	6	6.513283	4.877541	1.311994
16	6	5.098079	4.792860	1.900292
17	6	5.046581	4.722505	3.428021
18	6	4.436711	5.594724	4.268697
19	1	3.929283	6.484229	3.905005
20	6	4.470919	5.342548	5.698864
21	1	3.955691	6.020234	6.376518
22	6	5.158750	4.276594	6.166785
23	6	5.266801	3.743946	7.589474
24	6	6.679704	3.191374	7.773281
25	6	7.448735	3.037261	8.986268
26	1	7.121869	3.285030	9.987430
27	6	8.670052	2.558596	8.593094
28	1	9.532835	2.370601	9.217268
29	6	8.641133	2.480529	7.143711
30	6	9.753805	2.351266	6.306940
31	6	11.306182	4.008820	0.776751
32	6	12.046151	3.313971	1.795557
33	6	13.350645	2.928157	1.554370
34	1	13.921275	2.396209	2.310388
35	6	13.936843	3.230396	0.309101
36	1	14.962821	2.928524	0.117088
37	6	13.223948	3.904429	-0.671485
38	1	13.692406	4.129385	-1.625124
39	6	11.892659	4.299469	-0.440981
40	1	11.340027	4.825544	-1.214250
41	6	4.385664	6.076883	1.407110
42	6	4.961690	7.318400	1.723143
43	1	5.875010	7.346168	2.311564
44	6	4.382536	8.508616	1.295511
45	1	4.846794	9.455842	1.556391
46	6	3.215515	8.485336	0.530469
47	1	2.762489	9.413243	0.192517
48	6	2.643418	7.261291	0.199260
49	1	1.740174	7.226494	-0.404074
50	6	3.223833	6.066457	0.632175
51	1	2.765843	5.123684	0.354293
52	6	4.399250	3.507906	1.381265
53	6	4.969927	2.656162	0.431237
54	1	5.942977	2.882623	0.008316
55	6	4.306107	1.499404	0.016102
56	1	4.776284	0.850493	-0.717760
57	6	3.058098	1.176354	0.538032
58	1	2.543549	0.275567	0.215348
59	6	2.477443	2.018207	1.487625
60	1	1.505958	1.777330	1.910732
61	6	3.144483	3.164163	1.908410
62	1	2.694852	3.802667	2.663323
63	6	5.076500	4.891743	8.602307
64	6	5.923748	6.007760	8.521508
65	1	6.672330	6.051023	7.735198
66	6	5.814976	7.054390	9.430800
67	1	6.481159	7.908990	9.348152
68	6	4.857912	7.006226	10.445927
69	1	4.771617	7.823373	11.156926
70	6	4.018644	5.900797	10.540116
71	1	3.271969	5.847536	11.328019
72	6	4.127789	4.849804	9.626286
73	1	3.467684	3.993802	9.716749
74	6	4.216251	2.623046	7.783012
75	6	1.930088	1.831684	7.541092
76	1	0.913207	2.006239	7.199483
77	6	2.896293	2.816538	7.354163
78	1	2.626400	3.747079	6.864391
79	6	2.265612	0.625543	8.155989
80	1	1.514479	-0.147037	8.295459
81	6	3.573792	0.421230	8.585363
82	1	3.851722	-0.513722	9.064530
83	6	4.539656	1.411885	8.402952
84	1	5.554621	1.234487	8.744892
85	6	10.987630	1.855454	6.993515
86	6	12.112263	2.675720	7.167787
87	1	12.090056	3.693022	6.787272
88	6	13.239096	2.201944	7.835496
89	1	14.097418	2.854261	7.972142
90	6	13.265449	0.897965	8.330370
91	1	14.146915	0.527930	8.846812
92	6	12.154087	0.073529	8.162316
93	1	12.166384	-0.943578	8.544637
94	6	11.020650	0.551357	7.506893
95	1	11.877950	2.016952	4.710552
96	1	10.151628	-0.088017	7.379564

Cartesian coordinates for **3-Cl_2**:

Center Number	Atomic Number	Coordinates (Angstroms) X	Y	Z
1	78	1.217708	-0.563729	-0.151181
2	52	-1.211107	0.497623	-0.823018

3	7	1.995468	1.380863	0.191051	51	1	-0.291058	7.851496	1.026040
4	7	-1.844786	-2.042758	-0.349815	52	6	-0.055480	5.786640	0.477417
5	6	1.041046	-2.555858	-0.381593	53	1	-0.216036	5.986895	-0.576016
6	6	2.259907	-3.213948	-0.220666	54	6	0.133761	3.694057	-1.530297
7	6	3.398033	-2.423729	0.094635	55	6	1.201657	3.679293	-2.430970
8	1	4.384915	-2.846287	0.268547	56	1	2.183360	3.347730	-2.110377
9	6	3.139368	-1.076988	0.174643	57	6	1.019958	4.069398	-3.759112
10	6	3.987705	0.036131	0.429105	58	1	1.863523	4.036659	-4.442870
11	6	3.382440	1.288688	0.419937	59	6	-0.229261	4.485122	-4.207976
12	6	3.909220	2.610060	0.549010	60	1	-0.369151	4.785147	-5.242748
13	1	4.947349	2.863062	0.712324	61	6	-1.304066	4.502792	-3.318263
14	6	2.847279	3.462425	0.412118	62	1	-2.287932	4.818575	-3.654616
15	1	2.858912	4.542706	0.451323	63	6	-1.124358	4.105200	-1.997044
16	6	1.663383	2.661518	0.190637	64	1	-1.968917	4.108197	-1.313619
17	6	0.274932	3.266993	-0.047951	65	6	-4.499576	-1.047987	1.806735
18	6	-0.872905	2.302760	0.257892	66	6	-3.694358	-1.254262	2.938087
19	6	-1.824104	2.432723	1.213534	67	1	-2.612989	-1.199205	2.842036
20	1	-1.841667	3.281542	1.890880	68	6	-4.267318	-1.521417	4.177340
21	6	-2.842040	1.406817	1.349663	69	1	-3.626479	-1.675462	5.041170
22	1	-3.619922	1.521477	2.101621	70	6	-5.654830	-1.593561	4.310131
23	6	-2.796662	0.318050	0.550541	71	1	-6.102337	-1.800469	5.278423
24	6	-3.808909	-0.817531	0.445457	72	6	-6.459897	-1.401386	3.191372
25	6	-3.056101	-2.103251	0.125408	73	1	-7.541498	-1.459178	3.280171
26	6	-3.469205	-3.484534	0.351189	74	6	-5.886810	-1.133677	1.946176
27	1	-4.415323	-3.805829	0.765884	75	1	-6.528089	-0.989856	1.082971
28	6	-2.407922	-4.257684	-0.012093	76	6	-4.815028	-0.478984	-0.679339
29	1	-2.322480	-5.330061	0.089296	77	6	-6.404877	1.078941	-1.655878
30	6	-1.347597	-3.339419	-0.405669	78	1	-6.916276	2.037072	-1.618380
31	6	0.006615	-3.562834	-0.530464	79	6	-5.490069	0.749326	-0.660562
32	6	5.436517	-0.124939	0.677379	80	1	-5.291014	1.455328	0.139779
33	6	6.242600	-0.822471	-0.235477	81	6	-6.658491	0.189196	-2.700473
34	1	5.796883	-1.219272	-1.142953	82	1	-7.366245	0.449102	-3.482646
35	6	7.606716	-0.969834	0.000831	83	6	-5.991694	-1.031440	-2.732442
36	1	8.222895	-1.499576	-0.720313	84	1	-6.174841	-1.733593	-3.541177
37	6	8.181844	-0.434690	1.153169	85	6	-5.079828	-1.363738	-1.728895
38	1	9.245693	-0.555022	1.337536	86	1	-4.570678	-2.321463	-1.775027
39	6	7.387179	0.255331	2.068008	87	6	0.690181	-4.877807	-0.601004
40	1	7.828121	0.667787	2.971211	88	6	0.220183	-6.153216	-0.879806
41	6	6.024176	0.413851	1.832104	89	1	-0.824993	-6.332034	-1.110077
42	1	5.399498	0.934507	2.552036	90	6	1.127137	-7.223546	-0.893650
43	6	0.197090	4.483165	0.909841	91	1	0.767620	-8.226209	-1.105760
44	6	0.425854	4.269303	2.279574	92	6	2.479814	-7.010184	-0.648341
45	1	0.639193	3.264007	2.633763	93	1	3.169194	-7.849752	-0.662966
46	6	0.382968	5.323152	3.186694	94	6	2.967539	-5.718145	-0.399723
47	1	0.560269	5.130510	4.241334	95	1	4.028667	-5.556488	-0.231393
48	6	0.119856	6.620144	2.743593	96	6	2.072282	-4.661465	-0.387104
49	1	0.089094	7.445389	3.449675	97	17	1.744503	-0.355907	-2.457870
50	6	-0.094946	6.845663	1.387789	98	17	0.708372	-0.789328	2.142813
